# Synthesis and Anticancer and Antiviral Activities of C-2′-Branched Arabinonucleosides

**DOI:** 10.3390/ijms232012566

**Published:** 2022-10-19

**Authors:** Miklós Bege, Alexandra Kiss, Ilona Bereczki, Jan Hodek, Lenke Polyák, Gábor Szemán-Nagy, Lieve Naesens, Jan Weber, Anikó Borbás

**Affiliations:** 1 Department of Pharmaceutical Chemistry, University of Debrecen, Egyetem tér 1, 4032 Debrecen, Hungary; 2 Institute of Healthcare Industry, University of Debrecen, Nagyerdei krt 98, 4032 Debrecen, Hungary; 3 MTA-DE Molecular Recognition and Interaction Research Group, University of Debrecen, Egyetem tér 1, 4032 Debrecen, Hungary; 4 Department of Biotechnology and Microbiology, University of Debrecen, Egyetem tér 1, 4032 Debrecen, Hungary; 5 National Laboratory of Virology, University of Pécs, Ifjúság útja 20, 7624 Pécs, Hungary; 6 Institute of Organic Chemistry and Biochemistry, Czech Academy of Sciences, Flemingovo nam. 2, CZ-16000 Prague, Czech Republic; 7 Rega Institute for Medical Research, KU Leuven, B-3000 Leuven, Belgium

**Keywords:** photocatalytic thiol-ene reaction, nucleoside analogue, anti-tumor, time-lapse imaging, antiviral, coronavirus, SARS-CoV-2

## Abstract

d-Arabinofuranosyl-pyrimidine and -purine nucleoside analogues containing alkylthio-, acetylthio- or 1-thiosugar substituents at the C2’ position were prepared from the corresponding 3’,5’-*O*-silylene acetal-protected nucleoside 2’-exomethylenes by photoinitiated, radical-mediated hydrothiolation reactions. Although the stereochemical outcome of the hydrothiolation depended on the structure of both the thiol and the furanoside aglycone, in general, high d-arabino selectivity was obtained. The cytotoxic effect of the arabinonucleosides was studied on tumorous SCC (mouse squamous cell) and immortalized control HaCaT (human keratinocyte) cell lines by MTT assay. Three pyrimidine nucleosides containing C2’-butylsulfanylmethyl or -acetylthiomethyl groups showed promising cytotoxicity at low micromolar concentrations with good selectivity towards tumor cells. SAR analysis using a methyl β-d-arabinofuranoside reference compound showed that the silyl-protecting group, the nucleobase and the corresponding C2’ substituent are crucial for the cell growth inhibitory activity. The effects of the three most active nucleoside analogues on parameters indicative of cytotoxicity, such as cell size, division time and cell generation time, were investigated by near-infrared live cell imaging, which showed that the 2’-acetylthiomethyluridine derivative induced the most significant functional and morphological changes. Some nucleoside analogues also exerted anti-SARS-CoV-2 and/or anti-HCoV-229E activity with low micromolar EC_50_ values; however, the antiviral activity was always accompanied by significant cytotoxicity.

## 1. Introduction

Nucleoside analogues, which are chemically modified derivatives of natural nucleosides, have been key medicines in the treatment of cancer [1,2,3,4] and viral [4,5,6,7] diseases for decades. Antiviral and antiproliferative nucleoside analogues often contain modifications at the sugar unit, the most common of which include inversion of the 2’ configuration or formation of 2′-*C* branches. Several arabinonucleosides with inverted configuration at position 2′ such as cytarabine, fludarabine, clofarabine and nelarabine are approved for the treatment of hematological malignancies [8], and 2’-geminally difluorinated gemcitabine [9] is used to treat various types of cancer. The 2’-methyl-2’-fluorouridine derivative sofosbuvir is an effective medicine against hepatitis C infection [7,10]. In addition, many other 2′-*C*-branched nucleosides have been shown to possess potent anticancer [2] and antiviral [6,7] activities.

**Scheme 1:**
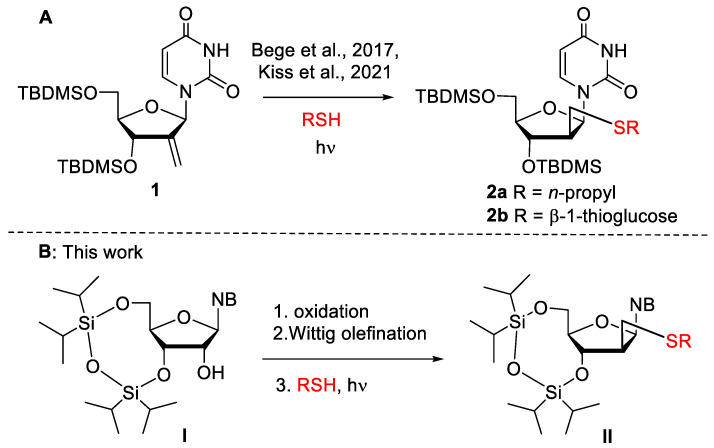
Synthesis of 2′-*C*-branched arabinonucleosides by photoinitiated thiol-ene coupling reaction (**A**): literature results [14,18], (**B**): this work.

We have recently shown that the photoinduced thiol-ene coupling, also known as thio-click reaction [11,12,13], is an efficient, atom economic strategy for the synthesis of sugar-modified nucleosides [14,15,16]. This radical-mediated hydrothiolation method provides rapid access to unique 2′- and 3′-branched nucleoside analogues with non-natural D-*arabino* and D-*xylo* configuration. We demonstrated that the addition of 1-propanethiol and 1-thiosugars to the 2’-exomethylene derivatives of 3’,5’-di-*O*-*tert-*butyldimethylsilyl (TBDMS) pyrimidine nucleosides occurred with high efficiency and almost exclusive D-*arabino* diastereoselectivity [14,17]. Among the 2′-*C*-branched arabinonucleoside derivatives obtained, the propylsulfanylmethyl derivative **2a** (Scheme 1A) showed good cytotoxic activity against tumorous cell lines (SCC and HeLa) but unfortunately with low selectivity [18].

In order to tune cytotoxic activity towards tumorous cell lines, we decided to prepare further 2’-*C*-branched arabinonucleosides and study their anticancer activity. Due to the large number of commercially available or readily preparable thiols, the thiol-ene coupling reaction provides a convenient and easy way to introduce a wide variety of substituents to the sugar ring of nucleosides. However, the silyl ether-protecting group strategy used in our previous work proved to be a serious limiting factor, as the required 3’,5’-di-*O*-TBDMS nucleoside was obtained only in very low yield (the main product of the reaction was the regioisomeric 2’,5’-di-*O*-TBDMS derivative). Therefore, 3’,5’-*O*-silylene acetal protection is used in the present work, which can be effectively formed in one step (Scheme 1B). We first investigated the effect of the new silylene acetal-protecting group on the efficiency and stereochemical outcome of the radical hydrothiolation reactions of pyrimidine and purine nucleoside alkenes. Subsequently, the cytotoxic effect of the sugar-modified nucleoside products was investigated on a healthy and a tumor cell line. The antiviral activity of the derivatives against H-CoV-229E and SARS-CoV-2 viruses was also evaluated. It is worth noting that although the development of anti-coronavirus nucleosides is a hot topic today [19,20,21] and the antiviral potential of sugar-modified nucleosides against RNA viruses is well known [5], the anti-SARS-CoV activity of 2’-modified nucleosides has not been studied.

## 2. Results and Discussion

### 2.1. Synthesis

**Scheme 2:**
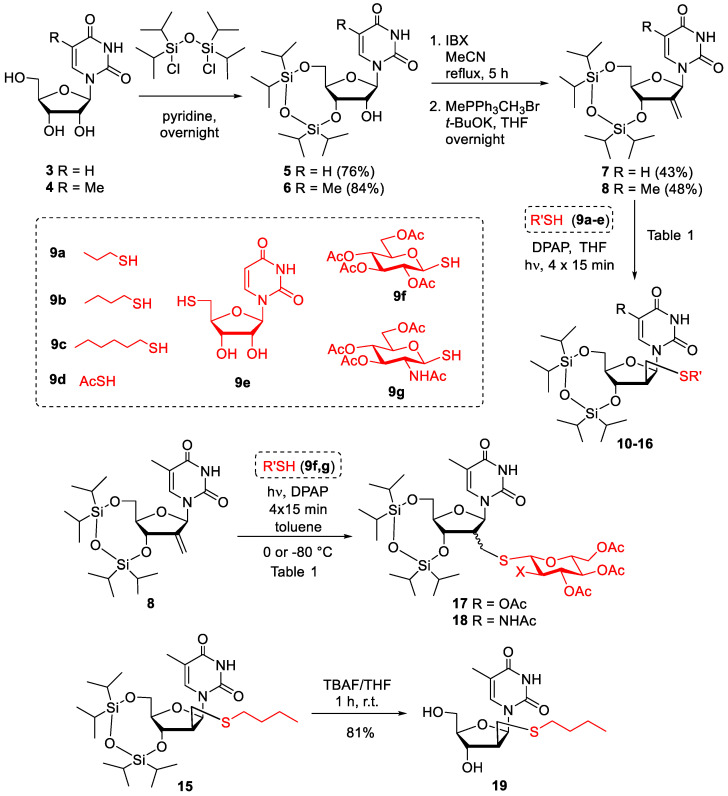
Synthesis of C2′substituted D-*arabino* configured nucleoside analogues **11**–**19**. IBX: 2-iodoxybenzoic acid, DPAP: 2,2-dimethoxy-2-phenylacetophenone, TBAF: tetrabutylammonium fluoride.

The 2′-exomethylene derivatives **7** and **8** were synthesized from uridine **3** and ribothymidine **4** in three steps, respectively, according to literature procedures (Scheme 2) [22,23,24]. First, the pyrimidine nucleosides were reacted with 1,3-dichloro-1,1,3,3-tetraisopropyldisiloxane (TIPDSCl) in pyridine to give the 3’,5’-silylene acetal derivatives **5** and **6** with high yields. Oxidation of the free 2′-OH with 2-iodoxybenzoic acid (IBX) followed by Wittig methylenation provided the required olefins **7** and **8**. The 2′-exomethylene derivatives were subjected to thiol-ene coupling reactions with thiols **9a**–**9g** under our previously optimized conditions at 0 °C using 4 × 15 min UV irradiation (λ_max_ 365 nm) in the presence of the photoinitiator 2,2-dimethoxy-2-phenylacetophenone (DPAP) [14,15,16]. It is known that the addition of thiols to terminal alkenes occurs through a radical chain mechanism resulting in a new C-S bond with complete regioselectivity according to the anti-Markovnikov-rule [11,17]. Furthermore, it is well established that radical hydrothiolation of exocyclic alkene substrates generally shows good to complete stereoselectivity [13,14,15,16,17]. The yield and diastereoisomeric ratio of the hydrothiolated products **10**–**18** are summarized in Table 1.

Addition of *n*-propyl, *n*-butyl and *n*-hexyl thiols **9a**–**9c** and thioacetic acid **9d** onto the exomethylene moiety of uridine **7** proceeded with good yields and high stereoselectivity providing the expected d-*arabino* configured isomers **10**–**13** as the major products. Similarly, reaction of ribothymidine alkene **8** with *n*-propyl and *n*-butyl thiols **9a** and **9b** yielded the 2′-*C*-branched alkylthioether products **14** and **15** with excellent d-*arabino* selectivity. To introduce a bulky and hydrophilic substituent to the 2′-position, compound **8** was reacted with 5′-thiouridine **9e** [25]. The addition reaction gave the expected arabinothymidine derivative **16** as the major product; however, both the yield and stereoselectivity were slightly lower than those observed for additions with simple alkyl thiols. It is important to note that no by-product formation was observed in any of the reactions. Where the yield was moderate, it was caused by the insufficient conversion of the starting alkene. Finally, alkene **8** was reacted with easily available β-1-thiosugars, tetra-*O*-acetyl-1-thioglucose **9f** and the *N*-acetylglucosamine derivative **9g**. Addition of 1-thioglucose across the double bond of **8** at 0 °C occurred with very low stereoselectivity, to give **17** as a stereoisomeric mixture containing almost equal proportions of d-arabinoside and d-riboside products. Running the reaction at −80 °C hardly improved the diastereoselectivity. This stereochemical outcome was very surprising because in our previous work, when the same thiol **9f** was reacted with the 3’,5’-di-*O*-TBDMS-protected alkene **1** at room temperature, a 4:1 mixture of the d-*arabin*o and d-*ribo* isomers was obtained, while at −80 °C, the product ratio was 10:1 in favor of the *arabino* isomer [14]. Addition of 1-thio-GlcNAc **9g** occurred also with low stereoselectivity providing **18** in a 2.3:1 ratio of the d-*arabino* and d-*ribo* isomers.

**Figure 1:**
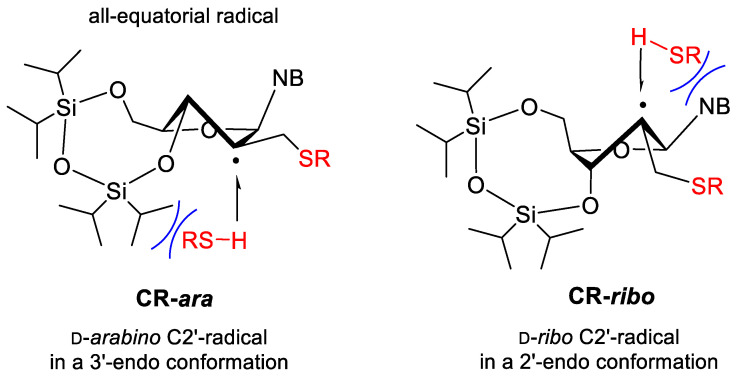
Structure of carbon-centered radical intermediates formed upon addition of thiyl radicals onto 2′-exocyclic double bond of nucleosides and direction of H-abstraction by these radicals.

The radical-mediated thiol-ene coupling is known to proceed via a reversible thiyl addition step followed by an irreversible H-abstraction step, and the stereoselectivity of the reaction of cyclic alkenes is dictated by the preferred axial H-abstraction by the carbon-centered radical intermediate [17,26]. The hydrothiolation reactions of **7** and **8** presumably proceed through the quasi-equatorial *arabino*- and *ribo*-configured radicals (**CR-ara**, **CR-ribo**) existing in 3’-endo or 2’-endo conformations (Figure 1), and the stereochemical outcome of the reactions is controlled by the relative stability of these radicals. Upon addition of sterically less demanding thiols (**9a**–**d**), the reactions preferably go through the more stable radical (**CR-ara**), which has a fully quasi-equatorial substitution pattern, leading to the high d-*arabino* selectivity observed. However, in the case of the bulky 1-thiosugars, the H-abstracion step is sterically hindered by the silylene acetal-protecting group from the bottom side, decreasing the proportion of the *arabino* isomer in the product. It should be noted here that when the silyl ether-protected 2’-exomethylene derivative **1** was reacted with 1-thioglucose **9f** in our previous work, a high degree of d-*arabino* selectivity was observed (10:1 *arabino*:*ribo* ratio at −80 °C) [14], which highlights that alkene-protecting groups can significantly affect the stereochemical outcome of the thiol-ene coupling reaction.

The *n*-butylthiomethyl-substituted pyrimidine nucleoside **15** was desilylated with tetrabutyl ammonium fluoride (TBAF) to give **19** in 81% yield (Scheme 2.). As compound **19** did not show cytotoxic activity (vide infra), deprotection of further derivatives was not performed.

**Scheme 3:**
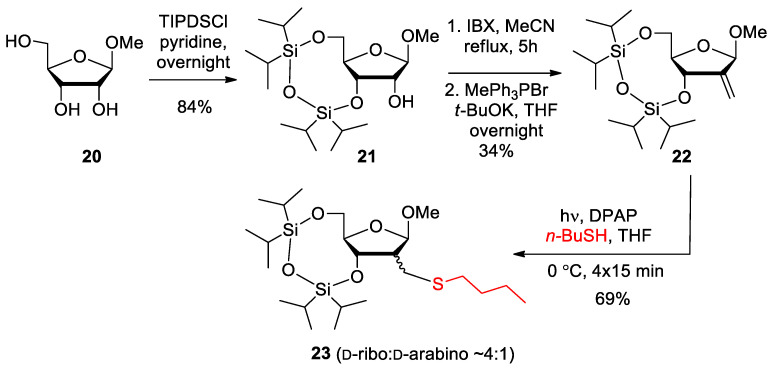
Synthesis of reference compound **23** from methyl β-d-ribofuranoside. TIPDSCl: 1,3-dichloro-1,1,3,3-tetraisopropyldisiloxane.

A 2’-branched derivative was also prepared from methyl β-d-ribofuranoside **20** to be used as a reference compound in biological assays. Compound **20** was treated with TIPDSCl; then, the 3′,5′-*O*-tetraisopropyldisiloxane acetal **21** [27] obtained was converted into 2′-exomethylene derivative **22** via oxidation followed by Wittig olefination (Scheme 3). The addition of *n*-butyl mercaptan (**9b**) to the exocyclic double bond of compound **22** occurred with the opposite stereoselectivity compared to the previous additions of the nucleoside exomethylenes **7** and **8**, providing the 2-C-butylsulfanylmethyl-ribofuranoside **23** as the major product. It is assumed that in the H-abstraction step, the methyl aglycone on the upper side of the furanose ring represents a smaller steric hindrance than the silylene acetal on the lower side; consequently, the reaction proceeds to a greater extent through the *ribo*-configured 2-C-centered radical intermediate than through the *arabino*-configured 2-C radical.

**Scheme 4:**
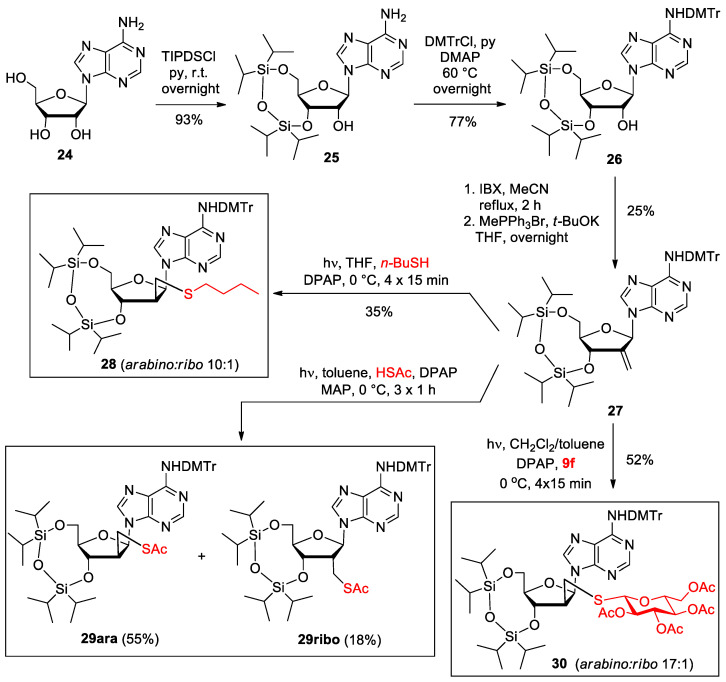
Synthesis of C2′ modified adenosine analogues **28**–**30.** DMTrCl: 4,4′-dimethoxytrityl chloride, MAP: 4-methoxyacetophenone.

Continuing the synthesis towards purine nucleoside analogues, adenosine **24** was 3′,5′-*O*-silylated to obtain **25** [28]; then, protection of the exocyclic amino group of the adenine residue with dimethoxytrityl group gave **26** (Scheme 4). Oxidation of **26** followed by Wittig olefination of the intermediate 2′-ulose afforded **27** which was subjected to thiol-ene reactions with *n*-butyl thiol **9b**, thioacetic acid **9d** and 1-thioglucose **9f**, respectively. The reactions with *n*-butyl thiol and 1-thiosugar proceeded with high d-*arabino* selectivity, although the conversion of the nucleoside alkene and thereby the yields of the thioether products **28** and **30** were only moderate. Interestingly, upon the addition of thioacetic acid onto **27** under the optimized conditions, no reaction was observed. Although we have previously found that thioacetic acid occasionally shows moderate reactivity in the thiol-ene coupling [29,30], the complete lack of reaction was surprising. We and others have demonstrated that the addition of HSAc can be promoted by using the MAP (4-methoxyacetophenone) photosensitizer in combination with the photoinitiator DPAP [30,31]. Applying this synergistic initiator system in the reaction of HSAc with **27** led to the efficient formation of the 2′-acetylthiomethyl product **29** with a 3:1 *arabino*:*ribo* ratio.

### 2.2. Cytotoxic Activity

**Figure 2:**
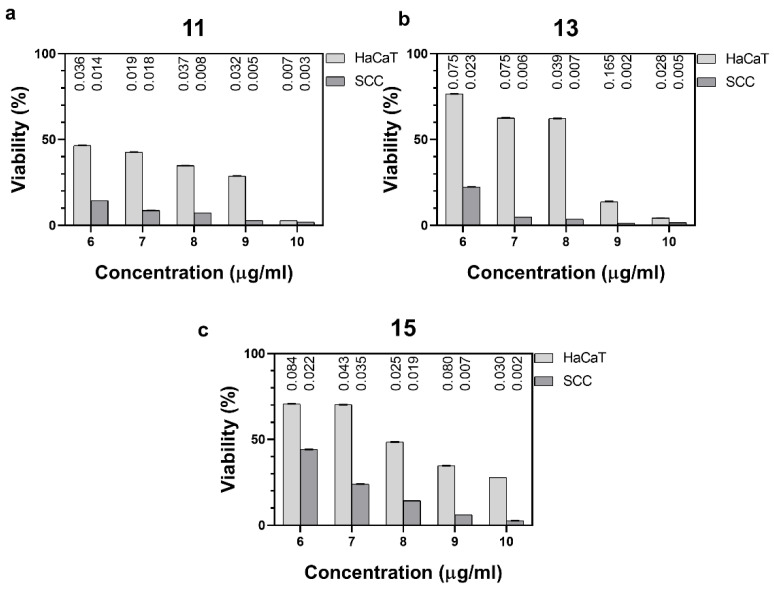
Effect of compounds **11** (**a**), **13** (**b**) and **15** (**c**) on viability of HaCaT and SCC cells by MTT assay.

The cytotoxic effect of the new nucleoside analogues was studied on tumorous SCC (mouse squamous carcinoma cell) and immortalized control HaCaT (human keratinocyte) cell lines. With the exception of compound **29**, the d-ribosyl and d-arabinosyl diastereomers could not be separated by column chromatography; therefore, the cytotoxicity tests were performed with the isomeric mixtures. The half-maximal inhibitory concentration (IC_50_) values of compounds are summarized in Table 2. The silylene acetal-protected ribothymidine derivatives **6** and **8** and the 2′-thiosubstituted uridine and ribothymidine analogues **10**–**16** inhibited cell growth in a concentration-dependent manner (Figure 2 and Figures S1 and S2) with IC_50_ values of 3.7–45.7 µM towards SCC cells and 8–20.3 µM towards HaCaT cells. The 1-thiosugar conjugates **17** and **18** (not shown), as well as the unprotected **19**, obtained by the desilylation of **15**, exerted no cytotoxicity to either cell line. The inactivity of **19** highlights the important role of silyl groups in the cell growth inhibitory activity of the nucleoside analogues which is supported by many literature results. For example, the *t*-butyldimethylsilyl (TBDMS)-protecting group has been shown to play a crucial role in the observed cytotoxic/antiproliferative activity of leinamycin-nucleosides [32], ureidoadenosines [33,34], 3′-modified xylofuranosyl nucelosides [15] and the ribothymidine-derived reverse transcriptase inhibitor TSAO-T [35]. Moreover, it has been reported that the introduction of silyl groups into nucleosides in itself endows the parent compounds with cytotoxic effects [36,37], which is consistent with the activity seen with silylated nucleosides **6** and **8**. At the same time, the cytotoxic effect and the selectivity towards the tumorous cell line can be significantly increased with appropriate synthetic modifications, as shown by the thiobutyl- and thioacetyl-modified compounds **11**, **13** and **15**, which showed high and selective cytotoxicity towards tumorous cells. At the same time, compound **16** bearing a thiouridine substituent at the C2’ position exerted opposite selectivity showing 2.5 times higher cell growth inhibition in the healthy cells over tumorous cells. The lack of activity of methyl riboside **23**, which carries the same *n*-butylsulfanylmethyl substituent as **11** and **15**, clearly indicates that the nucleobase is essential for the cell growth inhibitory effect.

The purine nucleoside analogues **28** and **29ara**, carrying a thiobutyl- and thioacetyl-substituent at position C2′, showed lower activity compared to their pyrimidine nucleoside congeners **11**, **13** and **15**. Nevertheless, the selective cytotoxicity of **29ara** to tumorous cells is remarkable. Moreover, the inactivity of the d-*ribo*-configured isomer, **29ribo**, pointed out the high impact of the C2′-configuration on the biological activity. The thiosugar-modified arabinoadenosine **30** was inactive, similarly to the arabinothymidine congeners **16** and **17**.

In order to better understand the mode of action of our nucleoside analogues, compounds **11**, **13** and **15** having the most promising activity profile were selected for further studies.

### 2.3. Live-Cell Imaging via Time-Lapse Microscopy

Dynamic functional and morphological effects of compounds **11**, **13** and **15** were investigated via time-lapse (1 frame/min) video microscopy under standard cell-breeding conditions. Low-intensity, near-infrared (NIR) 940 nm illumination was used for reduced phototoxicity. Parallel experiments were performed in the same CO_2_ incubator, using two custom-built inverted microscopes equipped with sensitive charge-coupled device (CCD) sensors. Cells were treated with a concentration of IC_50_ (half-maximal inhibitory concentration) specific for the SCC VII cell line (3.7 µM for **11**, 4.7 µM for **13** and 7.5 µM for **15**). Image sequences were processed and quantitatively analyzed using the NIH ImageJ open-source software bundle. Effect of the nucleoside analogues on the cell size, cellular generation time and cell growth of HaCaT and SCC VII cells was analyzed.

#### 2.3.1. Changes in the Size of Mother Cells

**Figure 3:**
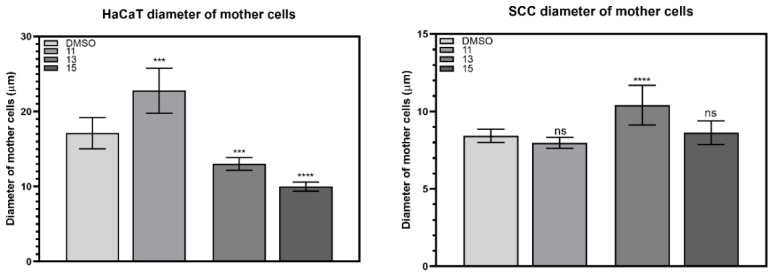
Effect of DMSO and compounds **11**, **13** and **15** on the mother cell sizes of HaCaT and SCC cells. (Asterisks and “ns” refer to the levels of significance of the data based on the statistical significance test; ns: not significant (*p* > 0.05), *** *p* < 0.001, **** *p* < 0.001; *p* = probability level).

Each cell line was treated with compounds **11** (3.7 µM), **13** (4.7 µM) and **15** (7.5 µM), dissolved in 1% (*v*/*v*) DMSO and diluted with cell culture media before application. Then, 1% (*v*/*v*) DMSO was used as control. Changes in cellular size, detached and rounded just prior to division, were measured (Figure 3) and given in percentage, relative to the DMSO control (Table 3). The increased size of the mother cells prior to division is a common indicator of cytotoxicity. Upon treatment with **11**, the size of HaCaT mother cells significantly increased while the size of SCC VII cells slightly decreased. Compound **15** changed the size of the mother cells in the opposite way, inducing a significant 41% size reduction in the HaCaT cell line and only a negligible increase in the size of the SCC VII cell line. At the same time, compound **13** significantly changed the mother cell size of both cell lines, increasing the size of tumorous mother cells by 24% while decreasing the size of HaCaT cells by the same extent.

#### 2.3.2. Duration of Cell Division

**Figure 4:**
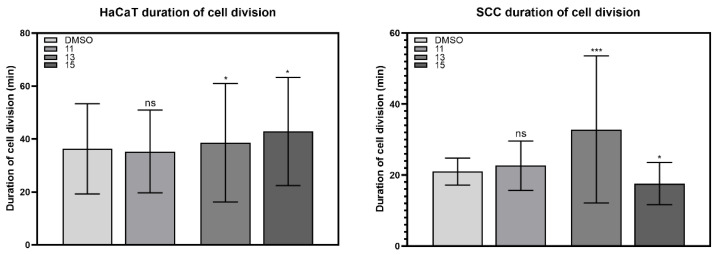
Effect of DMSO and compounds **11**, **13** and **15** on the duration of cell division of HaCaT and SCC cells. (Asterisks and “ns” refer to the levels of significance of the data based on the statistical significance test; ns: not significant (*p* > 0.05), * *p* < 0.05; *** *p* < 0.001; *p* = probability level.)

Compound **11** had only a small effect on the duration of cell division, slightly decreasing it for HaCaT and slightly increasing it for SCC VII cells. Compound **13** significantly increased the division time of SCC VII cells by 56%, while it had little effect on the HaCaT cell line. In the presence of compound **15**, smaller but significant changes were observed in both cell lines, the duration of cell division increased by 18% in HaCaT cells while decreasing by 16% in SCC cells (Table 4, Figure 4).

#### 2.3.3. Generation Time

For the investigation of the effect of compounds **11**, **13** and **15** on the cell cycle, cellular generation times were measured from time-lapse image sequences.

**Figure 5:**
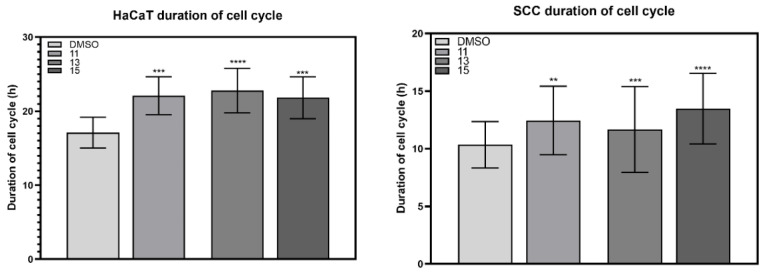
Effect of DMSO and compounds **11**, **13** and **15** on the generation time of HaCaT and SCC cells. (Asterisks refer to the levels of significance based on the statistical significance test; ** *p* < 0.01; *** *p* < 0.001; **** *p* < 0.0001; *p* = probability level.)

A change in the duration of the cell cycle also indicates cell toxicity. Treatment with compounds **11** and **15** significantly increased the cell cycle of both cell lines. After treatment with **11**, the generation time of HaCaT cells increased by 29% and that of SCC cells by 20%, and in the presence of **15**, this increase was 27% in HaCaT cells and 30% in SCC cells. Compound **13** greatly increased the generation time of the HaCaT cell line by 33% but caused a less significant increase of 13% in the generation time of the SCC VII cell line (Table 5, Figure 5).

### 2.4. Antiviral Evaluation

The anti-SARS-CoV-2 effect of the nucleoside was tested in VerE6 cells, and the anti-HCoV-229E evaluation was performed in HEL cells (Table 6). Unfortunately, only a few cytotoxic compounds exerted antiviral effect; the non-cytotoxic 1-thiosugar conjugates **16**, **17** and **30** and the deprotected **19** did not show activity against either of the two virus strains.

## 3. Methods and Materials

### 3.1. Chemistry

Compounds **5** [22], **6** [23], **7** [24], **8** [24], **9e** [12], **9f** [12], **9g** [25], **21** [27] and **25** [28] were prepared according to literature procedures (see SI for details). 2,2-Dimethoxy-2-phenylacetophenone (DPAP), 4-methoxyacetophenone (MAP), thioacetic acid, *n*-propyl mercaptan, *n*-butyl mercaptan and *n*-hexyl mercaptan were purchased from Sigma-Aldrich Chemical Co. and used without further purification. Optical rotations were measured at room temperature with a Perkin-Elmer 241 automatic polarimeter. TLC was performed on Kieselgel 60 F_254_ (Merck) with detection by UV-light (254 nm) and immersing into sulfuric acidic ammonium molybdate solution or 5% ethanolic sulfuric acid followed by heating. Flash column chromatography was performed on silica gel 60 (Merck, 0.040–0.063 mm). Organic solutions were dried over anhydrous Na_2_SO_4_ and concentrated in vacuum. ^1^H NMR (360 and 400 MHz) and ^13^C NMR (90 and 100 MHz) spectra were recorded with Bruker DRX-360 and Bruker DRX-400 spectrometers at 25 °C. Chemical shifts are referenced to Me_4_Si (0.00 ppm for ^1^H) and to the residual solvent signals (CDCl_3_: 77.2, DMSO-d_6_: 39.5, CD_3_OD: 49.0 for ^13^C). Two-dimensional COSY and ^1^H−^13^C HSQC experiments were used to assist NMR assignments. Characteristic NMR signals of the new nucleoside derivatives are summarized in Table S1. MALDI-TOF MS analyses of the compounds were carried out in the positive reflectron mode (20 kV) using a BIFLEX III mass spectrometer (Bruker, Germany) equipped with delayed-ion extraction or a Bruker Autoflex Speed mass spectrometer (Bruker, Germany). 2,5-Dihydroxybenzoic acid (DHB) was used as matrix and F_3_CCOONa as cationizing agent in DMF. 

The photoinitiated reactions were carried out in a borosilicate vessel by irradiation with a low-pressure Hg-lamp (Osram Supratec UV, HTC 150–211, 150 W, 230 V, R7s) giving maximum emission at 365 nm, without any caution to exclude air or moisture. The diastereomeric ratio of the products of the thiol-ene reactions was determined on the basis of their ^1^H NMR spectra.

#### 3.1.1. 2′-Deoxy-2′-C-propylsulfanylmethyl-3′,5′-O-(1,1,3,3-tetraisopropyldisiloxane-1,3-diyl)-β-D-arabinofuranosyl-uracil (**10**)

Exomethylene uridine **7** (200 mg, 0.41 mmol), *n*PrSH **9a** (308 µL, 3.3 mmol, 8.0 equiv.) and DPAP (10.6 mg, 0.041 mmol, 0.1 equiv.) were dissolved in THF (1 mL) and irradiated for 4 × 15 min at 0 °C. The reaction mixture was concentrated under reduced pressure. The crude product was purified by flash column chromatography (hexane/acetone 9/1) to give **10** (150 mg, 65% 14:1 *arabino:ribo* ratio) as a colorless syrup.

[α]_D_ = +18.9 (*c* = 0.18, CHCl_3_), Rf = 0.20 (hexane/acetone 8/2), ^1^H NMR (400 MHz, CDCl_3_) *δ* (ppm) 9.79 (s, 1H, N*H*), 7.62 (d, *J* = 8.1 Hz, 1H, H-6), 6.23 (d, *J* = 4.7 Hz, 1H, H-1′), 5.70 (d, *J* = 8.1 Hz, 1H, H-5), 4.35 (t, *J* = 8.8 Hz, 1H, H-3′), 4.11 (dd, *J* = 13.4, 1.8 Hz, 1H, H-5′a), 4.01 (dd, *J* = 13.1, 2.6 Hz, 1H, H-5′b), 3.75 (dt, *J* = 8.1, 2.4 Hz, 1H, H-4′), 2.88–2.84 (m, 2H, H-2′ &SCH_2a_), 2.59–2.50 (m, 1H, SCH_2b_), 2.39 (td, *J* = 7.1, 2.7 Hz, 2H, SC*H*_2_CH_2_CH_3_), 1.52 (dd, *J* = 14.6, 7.3 Hz, 2H, CH_2_C*H*_2_CH_3_), 1.11–0.99 (m, 28H 8 x *i*-PrC*H*_3_ & 4 x *i*-PrC*H*), 0.91 (t, *J* = 7.3 Hz, 3H, CH_2_CH_2_C*H*_3_). ^13^C NMR (100 MHz, CDCl_3_) *δ* (ppm) 163.9, 150.9 (2C, 2 x *C*O), 102.0 (1C, C-5), 84.2 (2C, C-1′, C-4′), 60.4 (1C, C-5′), 49.3 (1C, C-2′), 35.6 (1C, S*C*H_2_CH_2_CH_3_), 28.9 (1C, S*C*H_2_), 22.6 (1C, CH_2_*C*H_2_CH_3_), 17.5, 17.4, 17.3, 17.2, 17.1, 17.1, 17.0, 17.0 (8C, 8 x *i*-Pr*C*H_3_), 14.1, 13.4, 13.0, 12.8, 12.6 (5C, 4 x *i*-Pr*C*H & CH_2_CH_2_*C*H_3_). MALDI-ToF MS: *m*/*z* calcd for C_25_H_46_N_2_O_6_SSi_2_K^+^: 597.246 [M+K]^+^, found 597.303.

#### 3.1.2. 2′-Deoxy-2′-C-butylsulfanylmethyl-3′,5′-O-(1,1,3,3-tetraisopropyldisiloxane-1,3-diyl)-β-D-arabinofuranosyl-uracil (**11**)

Exomethylene uridine **7** (200 mg, 0.41 mmol), *n*BuSH **9b** (360 µL, 3.3 mmol, 8.0 equiv.) and DPAP (10.6 mg, 0.041 mmol, 0.1 equiv.) were dissolved in THF (1 mL) and irradiated for 4 × 15 min at 0 °C. The reaction mixture was concentrated under reduced pressure. The crude product was purified by flash column chromatography (hexane/acetone 9/1) to give **11** (164 mg, 69% with 20:1 *arabino:ribo* ratio) as a colorless syrup. 

[α]_D_ = +16.2 (*c* = 0.26, CHCl_3_), Rf = 0.32 (hexane/acetone 8/2), ^1^H NMR (400 MHz, CDCl_3_) *δ* (ppm) 9.77 (s, 1H, N*H*), 7.62 (d, *J* = 8.1 Hz, 1H, H-6), 6.23 (d, *J* = 4.4 Hz, 1H, H-1′), 5.69 (d, *J* = 8.1 Hz, 1H, H-5), 4.35 (t, *J* = 8.8 Hz, 1H, H-3′), 4.11 (dd, *J* = 13.3, 1.8 Hz, 1H, H-5′a), 4.01 (dd, *J* = 13.1, 2.7 Hz, 1H, H-5′b), 3.75 (dt, *J* = 8.1, 2.4 Hz, 1H, H-4′), 2.89–2.81 (m, 2H, H-2′ & SC*H*_2a_), 2.57–2.50 (m, 1H, SC*H*_2b_), 2.41 (td, *J* = 7.2, 2.0 Hz, 2H, SC*H*_2_CH_2_CH_2_CH_3_), 1.47 (dt, *J* = 14.6, 7.4 Hz, 2H, CH_2_C*H*_2_CH_2_CH_3_), 1.35–1.29 (m, 2H, CH_2_CH_2_C*H*_2_CH_3_), 1.11–0.98 (m, 28H, 8 x *i*-PrC*H*_3_ & 4 x *i*-PrC*H*), 0.86 (t, *J* = 7.3 Hz, 3H, CH_2_CH_2_CH_2_C*H*_3_). ^13^C NMR (100 MHz, CDCl_3_) *δ* (ppm) 163.9, 150.8 (2C, 2 x *C*O), 102.0 (1C, C-5), 84.2 (2C, C-1′, C-4′), 60.4 (1C, C-5′), 49.3 (1C, C-2′), 33.2 (1C, S*C*H_2_CH_2_CH_2_CH_3_), 31.4 (1C, SCH_2_*C*H_2_CH_2_CH_3_), 29.0 (1C, S*C*H_2_), 21.9 (1C, CH_2_CH_2_*C*H_2_CH_3_), 17.5, 17.4, 17.3, 17.2, 17.1, 17.1 (8C, 8 x *i*-Pr*C*H_3_), 14.1, 13.7, 13.0, 12.8, 12.6 (5C, 4 x *i*-PrCH & CH_2_CH_2_CH_2_*C*H_3_). MALDI-ToF MS: *m*/*z* calcd for C_26_H_48_N_2_O_6_SSi_2_K^+^: 611.262 [M+K]^+^, found 611.317.

#### 3.1.3. 2′-Deoxy-2′-C-hexylsulfanylmethyl-3′,5′-O-(1,1,3,3-tetraisopropyldisiloxane-1,3-diyl)-β-D-arabinofuranosyl-uracil (**12**)

Exomethylene uridine **7** (90 mg, 0.186 mmol), HexSH **9c** (211 µL, 1.49 mmol, 8.0 equiv.) and DPAP (4.8 mg, 0.019 mmol, 0.1 equiv.) were dissolved in THF (1 mL) and irradiated for 4 × 15 min at 0 °C. The reaction mixture was concentrated under reduced pressure. The crude product was purified by flash column chromatography (hexane/acetone 9/1) to give **12** (63 mg, 58%, 9:1 *arabino:ribo* ratio) as a colorless syrup.

[α]_D_ = +17.8 (*c* = 0.18, CHCl_3_), Rf = 0.25 (hexane/acetone 8/2), ^1^H NMR (400 MHz, CDCl_3_) *δ* (ppm) 7.64 (d, *J* = 8.0 Hz, 1H, H-6), 6.24 (d, *J* = 5.2 Hz, 1H, H-1′), 5.70 (dd, *J* = 8.1 Hz, 1H, H-5), 4.36 (t, *J* = 8.8 Hz, 1H, H-3′), 4.13 (dd, *J* = 13.2, 2.0 Hz, 1H, H-5′a), 4.03 (dd, *J* = 13.2, 2.8 Hz, 1H, H-5′b), 3.77 (dt, *J* = 8.1, 2.2 Hz, 1H, H-4′), 2.91–2.83 (m, 2H, H-2′ & SC*H*_2_a), 2.58–2.51 (m, 1H, SC*H*_2_b), 2.43 (td, *J* = 7.2, 3.6 Hz, 2H, Hexyl SC*H*_2_), 1.49 (dd, *J* = 14.7, 7.2 Hz, 2H, Hexyl C*H*_2_CH_3_), 1.35–1.21 (m, 6H, 3 x Hexyl C*H*_2_), 1.13–1.05 (m, 28H, 8 x *i*-PrC*H*_3_ & 4 x *i*-PrC*H*), 0.87 (t, *J* = 6.9 Hz, 3H, Hexyl C*H*_3_). ^13^C NMR (100 MHz, CDCl_3_) *δ* (ppm)163.7, 150.8 (2C, C-2, C-4), 102.1 (1C, C-5), 84.3 (2C, C-1′, C-4′), 49.4 (1C, C-2′), 33.7, 31.5, 29.4, 29.0, 28.6, 22.6 (6C, 6 x *C*H_2_), 17.6, 17.5, 17.4, 17.3, 17.3, 17.2 (8C, 8 x *i*-Pr*C*H_3_), 14.1, 13.1, 12.9, 12.6 (5C, 4 x *i*-PrCH & Hexyl *C*H_3_). MALDI-ToF MS: *m/z* calcd for C_28_H_52_N_2_O_6_SSi_2_Na^+^: 623.298 [M+Na]^+^, found 623.362.

#### 3.1.4. 2′-Deoxy-2′-C-acetylsulfanylmethyl-3′,5′-O-(1,1,3,3-tetraisopropyldisiloxane-1,3-diyl)-β-D-arabinofuranosyl-uracil (**13**)

Exomethylene uridine **7** (90 mg, 0.186 mmol), HSAc **9d** (104 µL, 1.49 mmol, 8.0 equiv.) and DPAP (4.8 mg, 0.019 mmol, 0.1 equiv.) were dissolved in THF (1 mL) and irradiated for 4 × 15 min at 0 °C. The reaction mixture was concentrated under reduced pressure. The crude product was purified by flash column chromatography (hexane/acetone 9/1→8/2) to give **13** (63 mg, 57%, 8:1 *arabino:ribo* ratio) as a colorless syrup.

[α]_D_ = +14.7 (*c* = 0.15, CHCl_3_), Rf = 0.25 (hexane/acetone 8/2), ^1^H NMR (400 MHz, CDCl_3_) *δ* (ppm) 7.67 (d, *J* = 8.1 Hz, 1H, H-6), 6.26 (d, *J* = 6.0 Hz, 1H, H-1′), 5.74 (d, *J* = 7.9 Hz, 1H, H-5), 4.24 (t, *J* = 8.9 Hz, 1H, H-3′), 4.14 (d, *J* = 13.3 Hz, 1H, H-5′a), 4.03 (d, *J* = 12.7 Hz, 1H, H-5′b), 3.77 (d, *J* = 8.2 Hz, 1H, H-4′), 3.32 (d, *J* = 11.0 Hz, 1H, SC*H*_2_a), 2.90–2.78 (m, 2H, H-2′ & SC*H*_2_b), 2.30 (s, 3H, AcC*H*_3_), 1.10–1.01 (m, 28H, 8 x *i*-PrC*H*_3_ & 4 x *i*-PrC*H*). ^13^C NMR (100 MHz, CDCl_3_) *δ* (ppm) 194.4 (1C, Ac*C*O), 163.4, 150.6 (2C, C-2, C-4), 140.7 (1C, C-6), 102.5 (1C, C-5), 84.2 (2C, C-1′, C-4′), 71.0 (1C, C-3′), 60.2 (1C, C-5′), 49.7 (1C, C-2′), 30.5 (1C, Ac*C*H_3_), 25.3 (1C, S*C*H_2_), 17.5, 17.5, 17.4, 17.2, 17.1 (8C, 8 x *i*-Pr*C*H_3_), 14.1, 13.1, 12.9, 12.7 (4C, 4 x *i*-PrCH). MALDI-ToF MS: *m*/*z* calcd for C_24_H_42_N_2_O_7_SSi_2_Na^+^: 581.215 [M+Na]^+^, found 581.264.

#### 3.1.5. 2′-Deoxy-2′-C-propylsulfanylmethyl-3′,5′-O-(1,1,3,3-tetraisopropyldisiloxane-1,3-diyl)-β-D-arabinofuranosyl-thymine (**14**)

Exomethylene thymidine **8** (200 mg, 0.40 mmol), *n*PrSH **9a** (300 µL, 3.2 mmol, 8.0 equiv.) and DPAP (10.3 mg, 0.040 mmol, 0.1 equiv.) were dissolved in THF (1 mL) and irradiated for 4 × 15 min at 0 °C. The reaction mixture was concentrated under reduced pressure. The crude product was purified by flash column chromatography (gradient elution hexane/acetone 9/1→85/15) to give **14** (169 mg, 74% with 20:1 *arabino:ribo* ratio) as a colorless syrup.

[α]_D_ = −17.0 (*c* = 0.10, CHCl_3_), Rf = 0.25 (hexane/acetone 8/2), ^1^H NMR (400 MHz, CDCl_3_) *δ* (ppm) 9.60 (s, 1H, N*H*), 7.33 (s, 1H, H-6), 6.24 (d, *J* = 4.1 Hz, 1H, H-1′), 4.38 (t, *J* = 8.6 Hz, 1H, H-3′), 4.13 (dd, *J* = 13.3, 1.2 Hz, 1H, H-5′a), 4.04 (dd, *J* = 13.0, 2.2 Hz, 1H, H-5′b), 3.76 (dt, *J* = 7.9, 2.4 Hz, 1H, H-4′), 2.82 (d, *J* = 4.1 Hz, 1H, H-2′), 2.51 (dd, *J* = 13.4, 8.7 Hz, 1H, SC*H*_2a_), 2.41 (t, *J* = 7.2 Hz, 2H, CH_3_CH_2_C*H*_2_S), 1.93 (s, 3H, thymineC*H*_3_), 1.54 (dd, *J* = 14.6, 7.3 Hz, 2H, CH_3_C*H*_2_), 1.16–0.99 (m, 28H, 4 x *i*-PrC*H*_3_ & 4 x *i*-PrC*H*), 0.93 (t, *J* = 7.3 Hz, 3H, PrC*H*_3_).^13^C NMR (100 MHz, CDCl_3_) *δ* (ppm) 164.1, 150.7 (2C, 2 x *C*O), 110.4 (1C, C-5), 84.1 (2C, C-1′, C-4′), 71.3 (1C, C-3′)*, 60.5 (1C, C-5′), 49.4 (1C, C-2′), 35.5 (1C, CH_3_CH_2_*C*H_2_S), 29.3 (1C, S*C*H_2_), 22.7 (1C, CH_3_*C*H_2_), 17.5, 17.4, 17.3, 17.2, 17.2 (8C, 8 x *i*-Pr*C*H_3_), 14.1, 13.4, 13.1, 12.7, 12.7 (6C, 4 x *i*-Pr*C*H & thymine *C*H_3_ & Pr*C*H_3_). *Can only be seen in HSQC. MALDI-ToF MS: *m*/*z* calcd for C_26_H_48_N_2_O_6_Si_2_Na^+^: 611.262 [M+Na]^+^, found 611.217.

#### 3.1.6. 2′-Deoxy-2′-C-butylsulfanylmethyl-3′,5′-O-(1,1,3,3-tetraisopropyldisiloxane-1,3-diyl)-β-D-arabinofuranosyl-thymine (**15**)

Exomethylene thymidine **8** (200 mg, 0.40 mmol), *n*BuSH **9b** (350 µL, 3.2 mmol, 8.0 equiv.) and DPAP (10.3 mg, 0.040 mmol, 0.1 equiv.) were dissolved in THF (1 mL) and irradiated for 4 × 15 min at 0 °C. The reaction mixture was concentrated under reduced pressure. The crude product was purified by flash column chromatography (hexane/acetone 9/1) to give **15** (138 mg, 59% 13:1 *arabino:ribo* ratio) as a colorless syrup.

[α]_D_ = -13.2 (*c* = 0.25, CHCl_3_), Rf = 0.34 (hexane/acetone 8/2), ^1^H NMR (400 MHz, CDCl_3_) *δ* (ppm) 9.61 (s, 1H, N*H*), 7.31 (s, 1H, H-6), 6.22 (s, 1H, H-1′), 4.35 (t, *J* = 8.3 Hz, 1H, H-3′), 4.11 (dd, *J* = 13.2, 1.7 Hz, 1H, H-5′a), 4.01 (dd, *J* = 13.1, 2.7 Hz, 1H, H-5′b), 3.73 (dt, *J* = 7.9, 2.2 Hz, 1H, H-4′), 2.84–2.79 (m, 2H, H-2′ & SC*H*_2a_), 2.49 (dd, *J* = 13.7, 8.7 Hz, 1H, SCH_2b_), 2.40 (t, *J* = 7.3 Hz, 2H, SC*H*_2_CH_2_CH_2_CH_3_), 1.90 (s, 3H, thymineC*H*_3_), 1.45 (dd, *J* = 14.8, 7.5 Hz, 2H, SCH_2_C*H*_2_CH_2_CH_3_), 1.33–1.28 (m, 2H, SCH_2_CH_2_C*H*_2_CH_3_), 1.14–0.99 (m, 28H, 8 x *i*-PrC*H*_3_ & 4 x *i*-PrC*H*), 0.85 (t, *J* = 7.3 Hz, 3H, SCH_2_CH_2_CH_2_C*H*_3_). ^13^C NMR (100 MHz, CDCl_3_) *δ* (ppm) 164.3, 150.8 (2C, 2 x *C*O), 110.3 (1C, C-5), 84.3, 84.0 (2C, C-1′, C-4′), 60.4 (1C, C-5′), 49.3 (1C, C-2′), 33.0 (1C, S*C*H_2_CH_2_CH_2_CH_3_), 31.4 (1C, SCH_2_*C*H_2_CH_2_CH_3_), 29.3 (1C, S*C*H_2_), 21.9 (1C, SCH_2_CH_2_*C*H_2_CH_3_), 17.5, 17.3, 17.2, 17.2, 17.1 (8C, 8 x *i*-Pr*C*H_3_), 14.1, 13.7, 13.0, 12.7, 12.6, 12.5 (6C, 4 x *i*-Pr*C*H, thymine *C*H_3_ & *n*-Pr*C*H_3_). MALDI-ToF MS: *m*/*z* calcd for C_27_H_50_N_2_O_6_SSi_2_K^+^: 625.278 [M+K]^+^, found 625.331.

#### 3.1.7. 2′-Deoxy-2′-C-(5′-deoxyuridine-5′-yl)-sulfanylmethyl-3′,5′-O-(1,1,3,3-tetraisopropyldisiloxane-1,3-diyl)-β-D-arabinofuranosyl-thymine (**16**)

Exomethylene thymidine **8** (130 mg, 0.261 mmol) was dissolved in a mixture of DMF and toluene. 5′-Thiouridine **9e** (88 mg, 0.314 mmol, 1.2 equiv.) and DPAP (7 mg, 0.026 mmol, 0.1 equiv.) were added and irradiated at 0 °C for 6 × 15 min. The solvent was evaporated under reduced pressure, and the crude product was purified by flash chromatography (CH_2_Cl_2_/MeOH 95/5→9/1) to give **16** (77 mg, 40%, with 7:1 *arabino:ribo* ratio) as a white solid.

[α]_D_ = +8.0 (*c* = 0.1, DMSO), Rf= 0.42 (CH_2_Cl_2_/MeOH 9/1), ^1^H NMR (400 MHz, MeOD) *δ* (ppm) 7.68 (d, *J* = 8.1 Hz, 1H, uridine H-6), 7.40 (s, 1H, ribothymidine H-6), 6.23 (s, 1H, ribothymidine H-1′), 5.86 (d, *J* = 4.2 Hz, 1H, uridine H-1′), 5.79 (d, *J* = 8.0 Hz, 1H, uridine H-5), 4.47 (s, 1H, ribothymidine H-3′), 4.22–4.15 (m, 2H, ribothymidine H-5′a & uridine H-2′), 4.12–4.04 (m, 2H, ribothymidine H-5′b & uridine H-3′), 4.03–3.97 (m, 2H, uridine H-4′), 3.89–3.81 (m, 1H, ribothymidine H-4′), 3.05 (dd, *J* = 12.8, 4.1 Hz, 1H, ribothymidine SC*H*_2_a), 3.00–2.91 (m, 1H, ribothymidine H-2′), 2.89 (d, *J* = 4.3 Hz, 2H, uridine H-5′ab), 2.67 (d, *J* = 7.4 Hz, 1H, ribothymidine SC*H*_2_b), 1.91 (s, 3H, thymine C*H*_3_), 1.22–1.09 (m, 28 H, 8 x *i*-PrC*H*_3_ & 4 x *i*-PrC*H*). ^13^C NMR (100 MHz, MeOD) *δ* (ppm) 165.7, 165.3, 152.3, 152.1 (4C, 4 x *C*O), 142.4 (1C, uridine C-6), 138.6* (1C, ribothymidine C-6) 110.9 (1C, ribothymidine C-5), 103.4 (1C, uridine C-5), 90.9 (1C, uridine C-1′), 85.2 (2C, ribothymidine C-4′, uridine C-4′), 74.5 (2C, ribothymidine C-3′, uridine C-2′), 73.4 (1C, uridine C-3′), 62.2* (1C, ribothymidine C-5′), 50.7* (1C, ribothymidine C-2′) 35.9 (1C, uridine C-5′), 31.7 (1C, ribothymidine S*C*H_2_), 18.2, 18.1, 17.9, 17.8, 17.8 (8C, 8 x *i*-Pr*C*H_3_), 15.0, 14.2, 13.8, 13.7 (4C, 4 x *i*-Pr*C*H), 12.9 (1C, thymine *C*H_3_). *Peaks can only be seen in HSQC. MALDI-ToF MS: *m*/*z* calcd for C_32_H_52_N_4_O_11_SSi_2_Na^+^: 779.2790 [M+Na]^+^, found 779.2782.

#### 3.1.8. 2′-Deoxy-2′-C-[(2,3,4,6-tetra-O-acetyl-β-D-glucopyranosylthio)methyl]-3′,5′-O-(1,1,3,3-tetraisopropyldisiloxane-1,3-diyl)-β-D-arabinofuranosyl-thymine (**17ara**) and 2′-deoxy-2′-C-[(2,3,4,6-tetra-O-acetyl-β-D-glucopyranosylthio)methyl]-3′,5′-O-(1,1,3,3-tetraisopropyldisiloxane-1,3-diyl)-β-D-ribofuranosyl-thymine (**17ribo**)

Method A: Compound **8** (100 mg, 0.20 mmol), 2,3,4,6-tera-*O*-acetyl-1-thio-β-D-glucopyranose **9f** (87.9 mg, 0.24 mmol, 1.2 equiv.) and DPAP (5.2 mg, 0.020 mmol, 0.1 equiv.) were dissolved in toluene (1 mL) and irradiated for 4 × 15 min at 0 °C. The reaction mixture was concentrated under reduced pressure. The crude product was purified by flash column chromatography (hexane/acetone 8/2→7/3) to give **17** (116 mg, 67%, inseparable mixture of the d-*arabino:* d-*ribo* products ~1.1:1) as white foam.

Method B: Compound **8** (100 mg, 0.20 mmol), 1-thiosugar **9f** (87.9 mg, 0.24 mmol, 1.2 equiv.) and DPAP (5.2 mg, 0.020 mmol, 0.1 equiv.) were dissolved in toluene (1 mL) and irradiated for 4 × 15 min at −80 °C. The reaction mixture was concentrated under reduced pressure. The crude product was purified by flash column chromatography (hexane/acetone 8/2→7/3) to give **17** (119 mg, 69%, inseparable mixture of the d-*arabino:*d-*ribo* products ~1.4:1) as white foam.

Rf = 0.49 (hexane/acetone 6/4), MALDI-ToF MS: *m*/*z* calcd for C_37_H_60_N_2_O_15_SSi_2_Na^+^: 883.3151 [M+Na]^+^, found 883.3215.

#### 3.1.9. 2′-Deoxy-2′-C-[(2-acetamido-3,4,6-tri-O-acetyl-2deoxy-β-D-glucopyranosylthio)methyl]-3′,5′-O-(1,1,3,3-tetraisopropyldisiloxane-1,3-diyl)-β-D-arabinofuranosyl-thymine (**18ara**) and 2′-deoxy-2′-C-[(2-acetamido-3,4,6-tri-O-acetyl-2deoxy-β-D-glucopyranosylthio)methyl]-3′,5′-O-(1,1,3,3-tetraisopropyldisiloxane-1,3-diyl)-β-D-ribofuranosyl-thymine (**18ribo**)

Compound **8** (100 mg, 0.20 mmol), 2-acetamido-2deoxy-3,4,6-tri-*O*-acetyl-1-thio-β-D-glucopyranose **9g** (87.9 mg, 0.24 mmol, 1.2 equiv.) and DPAP (5.2 mg, 0.020 mmol, 0.1 equiv.) were dissolved in a 2:1 mixture of toluene and MeOH (1 mL) and irradiated for 4 × 15 min at 0 °C. The reaction mixture was concentrated under reduced pressure. The crude product was purified by flash column chromatography (hexane/acetone 7/3→65/15) to give **18** (119 mg, 60%, inseparable mixture of the d-*arabino:*d-*ribo* products ~2.3:1) as a white solid.

Rf = 0.19 (hexane/acetone 6/4), NMR data of the major product: ^1^H NMR (400 MHz, CDCl_3_) *δ* (ppm) 7.42 (s, 1H, N*H*), 7.06 (t, *J* = 8.8 Hz, 1H, H-6), 6.36 (d, *J* = 7.2 Hz, 1H, H-1′), 5.41 (t, *J* = 9.7 Hz, 1H), 5.24–5.11 (m, 2H), 5.06 (ddd, *J* = 19.5, 13.0, 5.8 Hz, 2H), 4.26–4.09 (m, 7H), 3.87–3.70 (m, 4H), 3.11 (td, *J* = 11.2, 4.0 Hz, 1H), 2.98 (dd, *J* = 14.0, 3.5 Hz, 1H), 2.62–2.52 (m, 1H), 2.07 (s, 3H, AcC*H*_3_), 2.03 (s, 3H, AcC*H*_3_), 2.02 (s, 3H, AcC*H*_3_), 1.95 (s, 3H, AcC*H*_3_), 1.15–0.99 (m, 45H). ^13^C NMR (100 MHz, CDCl_3_) *δ* (ppm) 170.8, 170.8, 170.7, 169.6 (4C, 4 x Ac*C*O), 164.1, 151.9 (2C, 2 x thymine *C*O), 135.3 (1C, C-6), 111.6 (1C, C-5), 84.1, 83.5, 75.7, 73.4, 68.7, 62.6 (7C, skeletal carbons), 53.9, 50.1 (2C, C-2′, C-2′’), 27.6 (1C, SCH_2_), 23.2, 20.7 (4C, 4 x Ac*C*H_3_), 17.4, 17.3, 17.2, 17.1, 17.0 (8C, 8 x *i*-Pr*C*H_3_), 14.0, 12.9, 12.7, 12.6, 12.4 (5C, 4 x *i*-Pr*C*H & thymine *C*H_3_). MALDI-ToF MS: *m*/*z* calcd for C_37_H_61_N_3_O_14_SSi_2_Na^+^: 882.3310 [M+Na]^+^, found 883.3351.

#### 3.1.10. 1-[2′-Deoxy-2′-C-(n-butylsulfanylmethyl)-β-D-arabinofuranosyl]-thymine (**19**)

Compound **15** (165 mg, 0.28 mmol) was dissolved in dry THF (1 mL), and TBAF (0.56 mL of 1 M solution in THF) was added. The reaction mixture was stirred for 1 h at room temperature. The crude product was purified by flash column chromatography (EtOAc/MeOH 100/1) to give **19** (80 mg, 81%) as white foam.

[α]_D_ = +42.0 (*c* = 0.25, DMSO), Rf = 0.54 (EtOAc/MeOH 95/5), ^1^H NMR (400 MHz, MeOD) *δ* (ppm) 7.75 (s, 1H, H-6), 6.22 (d, *J* = 6.4 Hz, 1H, H-1′), 4.20 (t, *J* = 7.5 Hz, 1H, H-3′), 3.91 (dd, *J* = 12.2, 2.0 Hz, 1H, H-5′a), 3.77 (dd, *J* = 15.4, 3.1 Hz, 2H, H-5′b), 2.76–2.66 (m, 2H, H-2′), 2.48 (t, *J* = 7.2 Hz, 2H), 1.88 (s, 3H, thymine C*H*_3_), 1.53–1.45 (m, 2H, CH_3_CH_2_C*H*_2_CH_2_), 1.41–1.33 (m, 2H, CH_3_C*H*_2_CH_2_CH_2_), 0.89 (t, *J* = 7.3 Hz, 3H, BuC*H*_3_). ^13^C NMR (100 MHz, MeOD) *δ* (ppm) 166.4, 152.5 (2C, 2 x *C*O), 110.8 (1C, C-5), 86.3, 73.8 (3C, C-1′, C-3′, C-4′), 61.0 (1C, C-5′), 51.1 (1C, C-2′), 33.2, 32.7, 30.3, 22.8 (4C, 3 x Bu*C*H_2_ & S*C*H_2_), 14.0 (1C, Bu*C*H_3_), 12.4 (1C, thymine *C*H_3_), MALDI-ToF MS: *m*/*z* calcd for C_15_H_24_N_2_O_6_SNa^+^: 383.1253 [M+Na]^+^, found 383.1208.

#### 3.1.11. Methyl 2-deoxy-2-C-methylene-3,5-O-(1,1,3,3-tetraisopropyl-l,3-disiloxanediyl)-β-D-erythro-pentofuranoside (**22**)

Compound **21** (1.0 g, 2.45 mmol) was dissolved in dry MeCN (10 mL) and IBX (2.06 g, 7.37 mmol, 3.0 equiv.) was added and stirred at 100 °C for 5 h. The reaction mixture was diluted with EtOAc, filtered through celite and evaporated under reduced pressure. 

MePh_3_PBr (2.63 g, 7.37 mmol, 3.0 equiv.) was suspended in dry THF (20 mL), and *t*-BuOK (0.827 g, 7.37 mmol, 3.0 equiv.) was added and stirred at r.t. for 2 h. Then, the reaction mixture was cooled to −78 °C, and the ketone crude product (dissolved in dry THF) was added to it. The reaction mixture was allowed to warm to −10 °C over 1 h and stored at 0–4 °C overnight. The reaction mixture was diluted with CH_2_Cl_2_ and washed with saturated aq. NH_4_Cl solution and brine. The organic phase was dried over anhydrous Na_2_SO_4_ and concentrated in *vacuo*. The residue was purified by flash column chromatography (hexane/acetone 98/2) to yield **22** (337 mg, 34% over 2 steps).

Rf = 0.63 (hexane/acetone 14.5/0.5), ^1^H NMR (400 MHz, CDCl_3_) *δ* (ppm) 5.35 (d, *J* = 1.6 Hz, 1H, exomethylene H_a_), 5.33 (d, *J* = 2.6 Hz, 1H, exomethylene H_b_), 5.21 (s, 1H, H-1), 4.86–4.81 (m, 1H, H-3), 4.07 (dd, *J* = 11.7, 3.4 Hz, 1H, H-5a), 3.90 (dd, *J* = 11.6, 7.7 Hz, 1H, H-5b), 3.78 (td, *J* = 7.2, 3.4 Hz, 1H, H-4), 3.36 (s, 3H, OMe), 1.13–1.02 (m, 28H, 8 x *i*-PrC*H*_3_ & 4 x *i*-PrC*H*). ^13^C NMR (100 MHz, CDCl_3_) *δ* (ppm) 149.2 (1C, C-2), 110.1 (1C, exomethylene *C*H_2_), 103.5 (1C, C-1), 83.6, 73.4 (2C, C-3, C-4), 65.1 (1C, C-5), 54.2 (1C, OMe), 17.7, 17.5, 17.3, 17.1 (8C, 8 x *i*-Pr*C*H_3_), 13.6, 13.4, 12.9, 12.8 (4C, 4 x *i*-Pr*C*H).

#### 3.1.12. Methyl 2-C-(n-butylsulfanylmethyl)-2-deoxy-3,5-O-(1,1,3,3-tetraisopropyl-l,3-disiloxanediyl)-β-D-ribofuranoside (**23**)

Compound **22** (100 mg, 0.25 mmol), *n*BuSH (214 µL, 2.0 mmol, 8.0 equiv.) and DPAP (6.3 mg, 0.025 mmol, 0.1 equiv.) were dissolved in THF (1 mL) and irradiated for 4 × 15 min at 0 °C. The reaction mixture was concentrated under reduced pressure. The crude product was purified by flash column chromatography (hexane/Et_2_O 100/4) to give **23** (84 mg, 69% with 4.5:1 *ribo:arabino* ratio) as a colorless solid.

[α]_D_ = −100.8 (*c* = 0.12, CHCl_3_), Rf = 0.76 (hexane/acetone 14.5/5), ^1^H NMR (400 MHz, CDCl_3_) *δ* (ppm) 4.88 (d, *J* = 3.9 Hz, 1H, H-1), 4.19 (dd, *J* = 8.8, 5.5 Hz, 1H, H-3), 4.00 (dd, *J* = 11.1, 3.3 Hz, 1H, H-5a), 3.96–3.89 (m, 1H, H-4), 3.78–3.68 (m, 1, H-5bH), 3.30 (s, 3H, OMe), 2.74 (dd, *J* = 12.8, 4.3 Hz, 1H, SC*H*_2_a), 2.71–2.63 (m, 1H, SC*H*_2_b), 2.53 (q, *J* = 7.0 Hz, 2H, SC*H*_2_CH_2_CH_2_CH_3_), 2.44 (td, *J* = 9.4, 4.6 Hz, 1H, H-2), 1.58 (dt, *J* = 14.9, 7.3 Hz, 2H, SCH_2_C*H*_2_CH_2_CH_3_), 1.41 (dq, *J* = 14.3, 7.1 Hz, 2H, SCH_2_CH_2_C*H*_2_CH_3_), 1.10–1.00 (m, 28H, 8 x *i*-PrC*H*_3_ & 4 x *i*-PrC*H*), 0.92 (t, *J* = 7.3 Hz, 3H, SCH_2_CH_2_CH_2_C*H*_3_). ^13^C NMR (101 MHz, CDCl_3_) *δ* (ppm) 103.8 (1C, C-1), 85.0, 78.8 (2C, C-3, C-4), 66.7 (1C, C-5), 54.9, 53.6 (2C, C-2, OMe), 32.4, 31.8 (2C, 2 x S*C*H_2_), 28.9, 22.1 (2C, 2 x Bu*C*H_2_), 17.7, 17.6, 17.4, 17.1 (8C, 8 x *i*-Pr*C*H_3_), 13.8, 13.6, 13.5, 13.1, 12.7 (5C, 4 x *i*-Pr*C*H & Bu*C*H_3_). MALDI-ToF MS: *m*/*z* calcd for C_23_H_48_O_5_SSi_2_Na^+^: 515.266 [M+Na]^+^, found 515.326.

#### 3.1.13. 3′,5′-O-(1,1,3,3-Tetraisopropyldisiloxyl)-N-dimethoxytrityl-adenosine (**26**) [38]

Compound **25** (410 mg, 0.80 mmol) was dissolved in dry pyridine (5 mL), DMTrCl (553 mg, 1.6 mmol, 2.0 equiv.) was added, and the reaction mixture was stirred at r.t. overnight. Next day, the mixture was diluted with CH_2_Cl_2_ (200 mL) and washed with 10% aq. NaHSO_4_ solution. The organic phase was dried over Na_2_SO_4_, filtered and evaporated under reduced pressure. The crude product was purified by flash column chromatography (hexane/acetone 9/1→8/2) to give **26** (498 mg, 77%) as white foam.

[α]_D_= −35.7 (*c* = 0.21, CHCl_3_), Rf = 0.54 (hexane/acetone 6/4), ^1^H NMR (400 MHz, CDCl_3_) *δ* (ppm) 7.99, 7.86 (2 s, 2 x 1H, H-2, H-8), 7.36–7.31 (m, 2H, arom), 7.28–7.20 (m, 7H, arom), 6.91 (s, 1H, N*H*), 6.82–6.75 (m, 4H, arom), 5.89 (d, *J* = 1.0 Hz, 1H, H-1′), 5.15 (dd, *J* = 7.6, 5.6 Hz, 1H, H-3′), 4.61 (dt, *J* = 5.5, 1.3 Hz, 1H, H-2′), 4.12 (dd, *J* = 11.6, 4.1 Hz, 1H, H-5′a), 4.07 (dd, *J* = 7.7, 3.0 Hz, 1H, H-4′), 4.05–4.00 (m, 1H, H-5′b), 3.77 (s, 6H, 2 x OMe), 3.52 (s, 1H, O*H*) 1.12–1.03 (m, 28H, 4 x *i*-PrC*H*, 8 x *i*-PrC*H*_3_). ^13^C NMR (100 MHz, CDCl_3_) *δ* (ppm) 158.4 (2C, 2 x arom *C*_q_), 152.4 (1C, adenine *C*H), 154.3, 148.1 (2C, 2 x adenine *C*_q_), 145.5 (1C, Trt *C*_q_), 137.6 (2C, 2 x Trt *C*_q_), 130.2, 128.9, 127.9, 126.9, 113.2 (13C, arom.), 121.7 (1C, adenine *C*_q_), 89.9, 82.2, 75.0, 70.9 (4C, C-1′, C-2′, C-3′, C-4′), 70.7 (1C, Trt *C*_q_), 61.8 (1C, C-5′), 55.3 (2C, 2 x OMe), 17.6, 17.4, 17.2, 17.1 (8C, 8 x *i*-Pr*C*H_3_), 13.4, 13.2, 12.8, 12.7 (4C, 4 x *i*-Pr*C*H). MALDI-ToF MS: *m*/*z* calcd for C_43_H_57_N_5_O_7_Si_2_Na^+^: 834.369 [M+Na]^+^, found 834.464.

#### 3.1.14. 2′-Deoxy-N-dimethoxytrityl-3′,5′-O-(1,1,3,3-tetraisopropyldisiloxyl)--2′-C-methylene-adenosine (**27**)

Compound **26** (426 mg, 0.526 mmol) was dissolved in MeCN (10 mL), IBX (440 mg, 1.57 mmol, 3.0 equiv.) was added, and the mixture was stirred at 100 °C for 2.5 h. The suspension was diluted with EtOAc (50 mL) and filtered through celite. The filtrate was evaporated under reduced pressure to give a yellowish syrup.

Methyltriphenylphosphonium bromide (563 mg, 1.578 mmol, 3.0 equiv.) was suspended in dry THF (5 mL), and *t*-BuOK (177 mg, 1.578 mmol, 3.0 equiv.) was added under Ar. The suspension was stirred for 2 h at r.t. The reaction mixture was cooled to -78 °C, the ketone crude product (dissolved in 5 mL of dry THF) was added, and the reaction mixture was allowed to warm up to −10 °C over 1 h and stored at 0−4 °C overnight. The reaction mixture was diluted with CH_2_Cl_2_ (300 mL) and extracted with NH_4_Cl (3×) and brine (1×). The organic phase was dried over Na_2_SO_4_, filtered and evaporated under reduced pressure. The residue was purified by flash chromatography (hexane/acetone 9/1) to give **27** (102 mg, 25%) as a yellowish syrup.

[α]_D_= −41.9 (*c* = 0.21, CHCl_3_), Rf = 0.5 (hexane/acetone 7/3), ^1^H NMR (400 MHz, CDCl_3_) *δ* (ppm) 8.05, 7.83 (2 s, 2H, H-2, H-8), 7.46–7.38 (m, 1H), 7.35–7.17 (m, 11H, arom), 6.92 (s, 1H, N*H*), 6.82–6.73 (m, 4H, arom.), 6.57 (s, 1H, H-1′), 5.52 (s, 1H, H-exomethylene a), 5.42 (s, 1H, H-exomethylene b), 5.32 (d, *J* = 8.4 Hz, 1H), 4.13 (dd, *J* = 12.7, 3.6 Hz, 1H, H-5′a), 4.05 (dd, *J* = 12.9, 2.4 Hz, 1H, H-5′b), 3.77 (s, 6H, 2 x OMe), 1.13–1.04 (m, 28H, 4 x *i*-PrC*H*, 8 x *i*-PrC*H*_3_). ^13^C NMR (100 MHz, CDCl_3_) *δ* (ppm) 158.4, (2C, 2 x arom *C*_q_), 152.7 (1C, adenine *C*H), 154.2, 148.6, 147.6 (3C, 2 x adenine *C*_q_, C-2′), 145.5, 137.6 (3C, 3 x Trt *C*_q_), 130.2, 128.9, 127.9, 126.9, 113.2 (13C, arom.), 121.3 (1C, adenine *C*_q_), 111.9 (1C, exomethylene *C*H_2_), 83.4, 82.8, 71.6 (3C, C-1′, C-3′, C-4′), 70.7 (1C, Trt *C*_q_), 61.4 (1C, C-5′), 55.3 (2C, 2 x OMe), 17.5, 17.5, 17.4, 17.2, 17.1, 17.0 (8C, 8 x *i*-Pr*C*H_3_), 13.7, 13.2, 12.9, 12.7 (4C, 4 x *i*-Pr*C*H). MALDI-ToF MS: *m*/*z* calcd for C_44_H_57_N_5_O_6_Si_2_(-C_21_H_18_O_2_)Na^+^: 528.243 [M-DMTr+Na]^+^ (cleavage of the dimethoxytrityl group occurred), found 528.288.

#### 3.1.15. [2′-Deoxy-2′-C-butylsulfanylmethyl-3′,5′-O-(1,1,3,3-tetraisopropyldisiloxane-1,3-diyl)]-D-arabinofuranosyl-N-dimethoxytrityl-adenine (**28**)

Adenosine exomethylene **27** (200 mg, 0.248 mmol) was dissolved in THF (1 mL), and *n*-BuSH (215 µL, 2.0 mmol, 8.0 equiv.) and DPAP (6.4 mg, 0.025 mmol, 0.1 equiv.) were added and irradiated at 0 °C for 4 × 15 min. The solvent was evaporated under reduced pressure, and the crude product was purified by flash column chromatography (hexane/acetone 9/1) to give **28** (78 mg, 35% with 10:1 *arabino:ribo* ratio) as white foam.

[α]_D_= −40.0 (*c* = 0.19, CHCl_3_), Rf = 0.36 (hexane/acetone 8/2), ^1^H NMR (400 MHz, CDCl_3_) *δ* (ppm) 7.99, 7.83 (2 x s, 2 x 1H, H-2, H-8), 7.37-7-32 (m, 2H, arom.), 7.29–7.22 (m, 9H, arom.), 7.16 (d, *J* = 8.8 Hz, 2H, arom.), 6.92 (s, 1H, N*H*), 6.80 (dd, *J* = 12.4, 8.9 Hz, 6H, arom.), 6.25 (d, *J* = 7.6 Hz, 1H, H-1′), 5.14–5.05 (m, 1H, H-3′), 4.28 (dd, *J* = 12.0, 6.5 Hz, 1H, H-5′a), 3.98 (dd, *J* = 12.0, 3.1 Hz, 1H, H-5′b), 3.89 (td, *J* = 6.7, 3.0 Hz, 1H, H-4′), 3.77 (s, 6H, 2 x OMe), 2.96 (tdd, *J* = 11.2, 7.7, 4.0 Hz, 1H, H-2′), 2.76 (dd, *J* = 13.2, 3.9 Hz, 1H, SCH_2_a), 2.29 (t, *J* = 7.2 Hz, 2H), 2.13 (dd, *J* = 13.0, 11.2 Hz, 1H, SCH_2_b), 1.40–1.33 (m, 2H), 1.30–1.25 (m, 4H), 1.10–1.02 (m, 28H, 4 x *i*-PrC*H*, 8 x *i*-PrC*H*_3_), 0.84 (t, *J* = 7.2 Hz, 3H, BuMe). ^13^C NMR (100 MHz, CDCl_3_) *δ* (ppm) 158.4, 154.3 (2C, 2 x arom *C*_q_), 152.1, 141.0* (2C, C-2, C-8), 148.2, 145.6, 139.7, 137.6 (6C, 6 x arom C_q_), 130.2, 129.3, 128.9, 127.9, 127.1, 126.9, 121.6, 113.3 (13C, arom.), 85.7 (1C, C-1′), 85.0 (1C, C-4′), 74.9 (1C, C-3′), 70.7 (1C, DMTr *C*_q_), 62.9 (1C, C-5′), 55.3 (2C, 2 x OMe), 51.4 (1C, C-2′), 32.3, 31.4, 29.8, 29.1, 21.9 (4C, 4 x *C*H_2_), 17.7, 17.5, 17.5, 17.4, 17.2 (8C, 8 x *i*-Pr*C*H_3_), 13.7, 13.6, 13.2, 12.9, 12.8 (5C, 4 x *i*-Pr*C*H & Bu*C*H_3_). MALDI-ToF MS: *m*/*z* calcd for C_48_H_64_N_5_O_6_SSi_2_K^+^: 936.399 [M+K]^+^, found 936.536.

#### 3.1.16. [2′-Deoxy-2′-C-acetylthiomethyl-3′,5′-O-(1,1,3,3-tetraisopropyldisiloxane-1,3-diyl)]-D-arabinofuranosyl-N-dimethoxytrityl-adenine (**29ara**) and [2′-deoxy-2′-C-acetylthiomethyl-3′,5′-O-(1,1,3,3-tetraisopropyldisiloxane-1,3-diyl)]-adenosine (**29ribo**)

Adenosine exomethylene **27** (100 mg, 0.123 mmol) was dissolved in toluene and HSAc (52 µL, 0.738 mmol, 6.0 equiv.), MAP (9 mg, 0.0615 mmol, 0.5 equiv.) and DPAP (3 mg, 0.0123 mmol, 0.1 equiv.) were added, and the reaction mixture was irradiated at 0 °C for 3 × 1 h. Before the second irradiation, another 6 equiv. of HSAc was added. The solvent was evaporated under reduced pressure, and the crude product was purified by flash chromatography (hexane/acetone 95/5→9/1→8/2) to give **29ribo** (*ribo*) (20 mg, 18%) and **29ara** (*arabino*) (60 mg, 55%) as colorless syrups. 

Data of **29ribo** (*ribo* product): [α]_D_= −40.5 (*c* = 0.2, CHCl_3_), Rf = 0.34 (hexane/acetone 8/2), ^1^H NMR (400 MHz, CDCl_3_) *δ* (ppm) 8.01, 7.83 (2 s, 2 x 1H, H-2, H-8), 7.37-7-32 (m, 2H, arom.), 7.29–7.19 (m, 8H, arom.), 6.91 (s, 1H, N*H*), 6.80 (d, *J* = 8.9 Hz, 4H, arom.), 5.89 (s, 1H, H-1′), 5.12 (d, *J* = 7.6 Hz, 1H, H-3′), 4.29 (dd, *J* = 12.1, 5.9 Hz, 1H, H-5′a), 4.09 (ddd, *J* = 7.7, 6.0, 3.1 Hz, 1H, H-4′), 4.01 (dd, *J* = 12.1, 3.1 Hz, 1H, H-5′b), 3.78 (s, 6H, 2 x OMe), 3.40–3.29 (m, 2H, SC*H*_2_), 2.78 (d, *J* = 14.3 Hz, 1H, H-2′), 2.22 (s, 3H, AcC*H*_3_), 1.1–1.06 (m, 28H, 4 x *i*-PrC*H*, 8 x *i*-PrC*H*_3_). ^13^C NMR (100 MHz, CDCl_3_) *δ* (ppm) 194.6 (1C, Ac*C*O), 152.5 (1C, adenine *C*H), 158.4, 154.4, 147.9, 145.5, 137.6, 121.7 (8C, 8 x arom *C*_q_), 130.2, 128.9, 128.0, 126.9, 113.3 (13C, arom.), 90.8 (1C, C-1′), 82.6 (1C, C-4′), 81.4, 75.3 (1C, C-3′), 70.8 (1C, Trt*C*_q_), 62.5 (1C, C-5′), 55.4 (3C, 2 x OMe, C-2′), 33.5 (1C, S*C*H_2_), 30.5 (1C, Ac*C*H_3_), 17.6, 17.5, 17.2 (8C, 8 x *i*-Pr*C*H_3_), 13.7, 13.2, 12.8 (4C, 4 x *i*-Pr*C*H).

Data of **29ara** (*arabino* product): [α]_D_= −39.5 (*c* = 0.19, CHCl_3_), Rf = 0.4 (hexane/acetone 8/2), ^1^H NMR (400 MHz, CDCl_3_) *δ* (ppm) 8.02, 7.93 (2 s, 2 x 1H, H-2, H-8), 7.37-7-32 (m, 2H, arom.), 7.29–7.19 (m, 9H, arom.), 6.96 (s, 1H, N*H*), 6.83–6.75 (m, 5H, arom.), 6.69 (dd, *J* = 8.9, 1.9 Hz, 1H, arom.), 6.25 (d, *J* = 7.5 Hz, 1H, H-1′), 4.94 (t, *J* = 8.0 Hz, 1H, H-3′), 4.22 (dd, *J* = 12.5, 5.0 Hz, 1H, H-5′a), 4.00 (dd, 12.5, 3.1 Hz, 1H, H-5′b), 3.87 (ddd, *J* = 7.9, 4.9, 3.3 Hz, 1H, H-4′), 3.77 (s, 6H, 2 x OMe), 3.28 (dd, *J* = 13.7, 4.4 Hz, 1H, SC*H*_2_a), 2.96 (tdd, J = 10.2, 7.7, 4.5 Hz, 1H, H-2′), 2.51 (dd, *J* = 13.7, 10.5 Hz, 1H, SC*H*_2_b), 2.17 (s, 3H, AcC*H*_3_), 1.13–1.02 (m, 28H, 4 x *i*-PrC*H*, 8 x *i*-PrC*H*_3_). ^13^C NMR (100 MHz, CDCl_3_) *δ* (ppm) 194.1 (1C, Ac*C*O), 152.3, 139.2 (2C, C-2, C-8), 158.4, 154.3, 148.3, 145.5, 137.6, 121.5 (8C, 8 x arom *C*_q_), 130.3, 128.9, 127.9, 126.9, 113.2 (13C, arom.), 84.9, 84.7 (2C, C-1′, C-4′), 74.2 (1C, C-3′), 70.8 (1C, DMTr*C*_q_), 62.1 (1C, C-5′), 55.3 (2C, 2 x OMe), 51.1 (1C, C-2′), 30.5 (1C, Ac*C*H_3_), 25.9 (1C, S*C*H_2_), 17.6, 17.5, 17.5, 17.4, 17.2, 17.1 (8C, 8 x *i*-Pr*C*H_3_), 13.7, 13.2, 13.0, 12.7 (4C, 4 x *i*-Pr*C*H). MALDI-ToF MS: *m*/*z* calcd for C_46_H_61_N_5_O_7_SSi_2_Na^+^: 906.373 [M+Na]^+^, found 906.379.

#### 3.1.17. [2′-Deoxy-2′-C-[(2,3,4,6-tetra-O-acetyl-β-D-glucopyranosylthio)methyl]-3′,5′-O-(1,1,3,3-tetraisopropyldisiloxane-1,3-diyl)]-D-arabinofuranosyl-N-dimethoxytrityl-adenine (**30**)

Adenosine exomethylene **27** (200 mg, 0.248 mmol) was dissolved in a mixture of CH_2_Cl_2_ and toluene (2 mL), and 1-thioglucose-peracetate **9f** (108 mg, 0.296 mmol, 1.2 equiv.) and DPAP (6.4 mg, 0.025 mmol, 0.1 equiv.) were added and irradiated at 0 °C for 4 × 15 min. The solvent was evaporated under reduced pressure, and the crude product was purified by flash column chromatography (hexane/acetone 9/1→8/2) to give **30** (149 mg, 52% with 17:1 *arabino:ribo* ratio) as white foam.

[α]_D_ = −56.4 (*c* = 0.14, CHCl_3_), Rf = 0.37 (hexane/acetone 8/2), ^1^H NMR (400 MHz, CDCl_3_) *δ* (ppm) 8.00, 7.87 (2 s, 2 x 1H, H-2, H-8), 7.38–7.32 (m, 2H, arom.), 7.29–7.20 (m, 7H, arom.), 6.94 (s, 1H, N*H*), 6.80 (d, *J* = 8.8 Hz, 4H, arom.), 6.25 (d, *J* = 7.5 Hz, 1H, H-1′), 5.20 (t, *J* = 9.3 Hz, 1H, H-3′’), 5.16–5.08 (m, 1H, H-3′), 5.02 (dt, *J* = 19.1, 9.7 Hz, 2H, H-4′’ & H-2′’), 4.43 (d, *J* = 10.0 Hz, 1H, H-1′’), 4.27 (dd, *J* = 11.7, 5.8 Hz, 1H, H-5′a), 4.24 (dd, *J* = 12.4, 4.7 Hz, 1H, H-6′’a), 4.13 (dd, *J* = 12.3, 1.8 Hz, 1H, H-6′’b), 3.97 (dd, *J* = 12.1, 2.9 Hz, 1H, H-5′b), 3.89 (td, *J* = 6.7, 3.0 Hz, 1H, H-4′), 3.77 (s, 6H, 2 x OMe), 3.70 (ddd, *J* = 9.8, 4.1, 2.1 Hz, 1H, H-5′’), 3.23–3.12 (m, 1H, H-2′), 3.08 (dd, *J* = 13.9, 3.9 Hz, 1H, SC*H*_2_a), 2.27 (dd, *J* = 13.7, 11.1 Hz, 1H, SC*H*_2_b), 2.08 (s, 3H, AcC*H*_3_), 2.02 (s, 3H, AcC*H*_3_), 1.99 (s, 3H, AcC*H*_3_), 1.98 (s, 3H, AcC*H*_3_), 1.14–1.06 (m, 28H, 4 x *i*-PrC*H*, 8 x *i*-PrC*H*_3_). ^13^C NMR (100 MHz, CDCl_3_) *δ* (ppm) 170.6, 170.1, 169.4, 169.1 (4C, 4 x Ac*C*O), 158.3, 154.3 (2C, 2 x arom *C*_q_), 152.1, 140.7* (2C, C-2, C-8), 148.1, 145.5, 137.5 (5C, 5 x arom C_q_), 130.2, 128.9, 127.9, 126.8, 113.2 (13C, arom.), 121.6 (1C, adenine *C*_q_), 85.5 (1C, C-1′), 84.9 (1C, C-4′), 82.9 (1C, C-1′’), 76.2 (1C, C-5′’), 75.3 (1C, C-3′), 73.8 (1C, C-3′’), 70.7 (1C, Trt*C*_q_), 68.9, 68.1 (2C, C-2′’ & C-4′’), 62.8 (1C, C-5′), 61.9 (1C, C-6′’), 55.2 (2C, 2 x OMe), 52.3 (1C, C-2′), 26.2 (1C, S*C*H_2_), 20.8, 20.6 (4C, 4 x Ac*C*H_3_), 17.6, 17.4, 17.4, 17.1, 17.1 (8C, 8 x *i*-Pr*C*H_3_), 13.5, 13.2, 12.9, 12.8 (4C, 4 x *i*-Pr*C*H). *Peak can only be seen in HSQC. MALDI-ToF MS: *m/z* calcd for [(C_58_H_77_N_5_O_15_SSi_2_-C_21_H_18_O_2_)+Na]^+^ 892.326 [M-DMTr+Na]^+^ (cleavage of the dimethoxytrityl group occurred), found 892.412.

### 3.2. Cell Viability Studies

Spontaneously transformed, aneuploid keratinocytic, adherent cell type was derived from the epithelium of human Caucasian adult male skin. The cell line was immortalized in 1986 under low-calcium and high-temperature conditions [39]. Cells were cultured in DMEM medium (iBiotech, Szigetszentmiklós, Hungary) containing 10% fetal bovine serum (FBS, iBiotech, Szigetszentmiklós, Hungary) and 1% antibiotic-antimycotic mix (Penicillin–Streptomycin–Neomycin) [40]. SCC-VII is a carcinoma tumor that originally arose in the abdominal wall of C3H mice in Dr. Herman Suit’s laboratory at Harvard University, Boston, Massachusetts. SCC VII cells were grown in Dulbecco’s Modified Eagle’s Medium Nutrient Mixture (DMEM-HAM’S F12) (iBiotech, Szigetszentmiklós, Hungary) supplemented with 2 mM L-glutamine, 23 mM NaHCO_3_, 100 U/mL penicillin, 100 U/mL streptomycin, 1% non-essential amino acids and 10% fetal bovine serum [41]. In LTS video-microscopy studies, ~150,000/75,000 starting cell numbers (glass bottom dish) had been used. Cell cultures had been treated with compounds **11, 13** and **15** using the IC_50_ concentration corresponding to the SCC VII.

#### MTT Assay

Cell cultures were kept under the same conditions during the experiments. Cells were grown at 37 °C, 95% humidity and 5% CO_2_ in the incubator. After three days of incubation in cell culture flasks, cells were trypsinized, and homogeneous cell suspensions were formed. Aliquots (500 μL) of each cell suspension containing the inhibitor at the final concentration were placed in wells of 24-well-plates. During the MTT assay, different starting cell numbers were used due to cell cycle differences; HaCaT were started at 50,000 cells/well, while SCC VII were started at 25,000 cells/well. The different starting conditions were applied to compensate for the faster generation time and consequently increased protein mass of the tumorous cell line [42]. After subculturing, 24 h was allowed for the cells to adhere to the bottom of the wells. In both cases, the cells were treated with the compounds at ~50% confluence [43]. According to our protocol, an aliquot of the desired final volume of medium is given first and pre-warmed in a water bath (typically for 15 min to equilibrate to 37 °C) before adding the agent to be tested. Commonly, 500 μL of the medium is used per well of a 24-well plate. The medium and agent were mixed by suspension or vortexing. The agent was dissolved in 1% DMSO and diluted with prewarmed DMEM/DMEM-F12 in 1% (*v*/*v*) DMSO concentration and placed into the wells of plates [44]. The cells were incubated for a further 48 h under the breeding conditions. The control sample contained only DMSO in a prewarmed medium. The following analogue concentrations were tested: 6, 7, 8, 9 and 10 μg/mL. 

The IC_50_ value of a compound was defined as the concentration that inhibits cell growth by 50% relative to the control. After the MTT test, the medium containing the inhibitor was removed, and 100 μL MTT solution was added to the cells. The plates were then incubated for 2 h at 37 °C. The medium from the wells was then carefully aspirated and MTT formazan dissolved with 100 μL of DMSO aided by gentle agitation on a shaker. After 10 min at room temperature, the absorbances were read at 570 nm by an automatic plate reader. The percentage of viability reflecting the respiratory potential of the cell population in each well was expressed as absorbance of treated cells/absorbance of control cells) ×100. IC_50_ values of tested agents were determined by Graphpad (Prism) semi-log line fitting (graphpad.com (8)). The MTT test was performed after treatment and removal of inhibitors followed by the addition of 100 μL of MTT solution to the cells.

### 3.3. Time-Lapse Image Video-Microscopy and Image Analysis

A SANYO MCO18-AC (Wood Dale, IL, US) CO2 incubator was used with a back-side instrument port. Its chamber was modified to host four microscopes. Olympus (Tokyo, Japan) upright microscopes were modified for inverted usage, and revolver turrets were installed to replace the original illumination. The charge-coupled device (CCD) camera boards were placed under the turrets, using the monocular adapter lower parts of Olympus Tokyo as housing. Specimen tables were unmodified, but the slide orientation mechanisms were removed. Ocular sockets were used for illuminators. Microscope objectives: Carl Zeiss (Jena, Germany) plan achromatic objectives (×10: 0.25 NA) that were used to enable a broad field of view to be imaged. Cameras: High-sensitivity digital (SSC) camera. KPC EX-20H high-resolution camera (KT&C, Seoul, Korea) with a Sony ExView CCD sensor. Illumination intensity at F 1.2:0.003 lux High 900–1000 nM near-infrared range sensitivity. Native frame rate: 25 frames/s [45].

Ten images were collected, each within a 5 s interval and averaged to minimize noise. The collection of images within 5 s is regarded as high time resolution. Long-term scan (LTS) system: Illumination was provided by diodes emitting light at 940 nm (LED: 5 mm diameter; 1.2 V and 50 mA, driven at 5 V using a serial 82 Ohm resistor) used to illuminate cells while minimizing heat and phototoxicity. The longer wavelength offered deeper penetration (up to 3 mm thickness) and less light dispersion through the culture medium and the wall of the T-flask. The theoretical limit of resolution under our conditions using a 940 nm wavelength at 1.25 numerical aperture was 1.88 nm based on the Abbe equation. The original 5 mm spherical light-emitting diode (LED) head was used as a condenser for better reproducibility of the setup. Illuminators were centered and fixed with glue in ocular microscope tubes. The distance between the upper surface of the T-25 culture flask and the spherical ball head of the LED was 120 mm. Illumination of cells lasted only during image acquisition periods (~5 s per time point) [46]. National Health Institute’s ImageJ software was used to analyze the image sequences of the time-lapse video microscopy [47].

For each study, cells were grown in a medium containing 1% DMSO at. Cells were treated with compounds **11**, **13** and **15** at their corresponding IC_50_ concentrations specific to SCC cells (3.7 µM of **11,** 4.7 µM of **13** and 7.5 µM of **15**). Cellular changes were registered and given in %. Results are given as differences in percentages after treatment relative to the DMSO control (100%). Characteristic data of parameters are given by the average values of n = 30 samples.

#### 3.3.1. Cell Size Measurements

Divided cells were selected from the binary image sequence based on their circularity determined by area/perimeter ratio. Larger pre-division mother cells were separated from smaller post-division daughter cells. Pixel size was calibrated with a Burker chamber. Diameter calculation implied pixel to μm transformation.

#### 3.3.2. Determination of the Time of Cell Division

Cell division starts with the lifting up of mitotic mother cells and dividing. It lasts until daughter cells settle down and attach to the growth surface. The time of cell division was determined manually using the Orthogonal Views function of the Fiji program.

#### 3.3.3. Determination of Generation Time

Images were acquired at a frame rate of 1 frame per minute. Two daughter cells of a dividing mother cell were followed in time until their next division, resulting in 2 pairs of new daughter cells.

### 3.4. Antiviral Assays

#### 3.4.1. SARS-CoV-2 Infection, Cytopathic Effect (CPE) Detection, Compound Screening

Anti-SARS-CoV-2 activity of all compounds was screened at four concentrations in the first step to choose active compounds for EC_50_ and CC_50_ measurement. Compounds (100-40-16-6.4 µM concentration) were tested in VERO E6 cells (5000 cells/25 µL) in 384-well plate format. Compound was added to the cells, and after one hour, cells were infected with SARS-CoV-2 (MOI 0.05). Cells were incubated for 72 h in CO_2_ incubator set to 37 °C, and after incubation, cytopathic effect (CPE) was analyzed by XTT colorimetric assay. Briefly, 50 μL of 50:1 mixture of XTT labeling reagent (1 mg/mL) and PMS electron-coupling reagent (0.383 mg/mL) was added to the wells and incubated for 4 h in 37 °C in 5 % CO_2_. Formation of orange formazan dye was measured in EnVision plate reader.

Anti-SARS-CoV-2 EC_50_ of selected compounds was tested in VERO E6 cells (15,000 cells/100 µL) in 96-well plate format (Figure S3). Compounds were tested from 100 µM concentration using 2-fold dilution. Compound was added to the cells, and after one hour, cells were infected with SARS-CoV-2 (MOI 0.05). Cells were incubated for 72 h in CO_2_ incubator set to 37 °C, and after incubation, cytopathic effect (CPE) was analyzed by XTT colorimetric assay.

#### 3.4.2. Cytotoxicity in VERO E6 cells (XTT Assay CC_50_ Measurement)

Cytotoxity was measured in VERO E6 cells (15,000 cells/100 µL) in 96-well plate format (Figure S4). Compound cytotoxicity was tested at the same concentration range as EC_50_ measurement. Cytotoxicity was detected after 72 h using XTT colorimetric assay.

## 4. Conclusions

d-*Arabino*-configured thymidine, uridine and adenosine analogues were efficiently prepared by photoinduced thiol-ene reactions between 3′,5′-*O*-silylene-acetal-protected nucleoside 2′-exomethylene derivatives and various thiols including alkylthiols, thioacetic acid and 1-thiosugars. The cytotoxic effect of these compounds was studied on tumorous SCC and healthy HaCaT cell lines by MTT assay. Among the sixteen tested nucleoside analogues, eleven derivatives showed concentration-dependent cytotoxicity. In the case of compounds containing the same C2’ modifying group, pyrimidine nucleosides showed higher activity than adenosine derivatives, while all 1-thiosugar-containing derivatives were inactive, independently of the type of nucleobase. The silyl-protecting group, the nucleobase and an appropriate C2’ substituent were shown to be crucial for cell growth inhibitory activity.

The pyrimidine nucleoside analogues **11**, **13** and **15**, bearing a butylsulfanylmethyl or an acetylthiomethyl substituent, showed the best cytotoxicity profiles, significantly reducing the viability of tumorous SCC cells at low micromolar concentrations while barely affecting the viability of healthy HaCaT cells. The effect of the best three compounds, **11**, **13** and **15**, on cell size, time of division and cellular generation time was studied by time-lapse microscopy. The most significant increase in the size of the mother cells of the tumorous cell line was caused by compound **13**. The duration of cell division was significantly changed in both cell lines by treatment with **15**. Remarkably, **13** selectively modified the division time only in SCC cells, significantly increasing it by 65%, but at the same time insignificantly changed the cell division time in HaCaT cells. Generation time was significantly increased by all three compounds. Although, according to the MTT assay, compound **11** inhibited tumor cells somewhat more strongly and with greater selectivity than compound **13**, in the time-lapse microscopy studies, compound **13** caused the most significant changes in the cellular parameters indicating cytotoxicity. Therefore, the 2’-acetylthiomethyl uridine derivative **13** can be considered a leading structure in the design of new, more effective antitumor compounds.

Only a few compounds showed anti-SARS-CoV-2 and/or anti-HCoV-229E effect, and, unfortunately, the antiviral activity was always accompanied by significant cytotoxicity.

## Figures and Tables

**Table 1:** Photoinitiated hydrothiolation reactions of 2′-exomethylene nucleosides **7** and **8** ^a^.

Alkene	R’	Temperature	Product	*arabino:ribo* ^b^	Yield
7	*n*-propyl	0 °C	10	14:1	65%
7	*n*-butyl	0 °C	11	20:1	69%
7	*n*-hexyl	0 °C	12	9:1	58%
7	acetyl	0 °C	13	8:1	57%
8	*n*-propyl	0 °C	14	20:1	74%
8	*n*-butyl	0 °C	15	13:1	67%
8	uridine-5′-yl ^c^	0 °C	16	7:1	42% ^c^
8	Glc (OAc) _4_	0 °C	17	1.1:1	67%
8	Glc (OAc) _4_	−80 °C	17	1.4:1	69%
8	GlcNAc(OAc) _3_	0 °C	18	2.3:1	60%

^a^ The reaction was carried out in a borosilicate vessel without any caution to exclude air or moisture ^b^ The diastereoisomeric ratio was determined by ^1^ H NMR. ^c^ The reaction was performed in toluene: DMF 2:1.

**Table 2:** Cytotoxic activity of the compounds on healthy (HaCaT) and tumorous (SCC) cell lines.

Compound	HaCaT IC_50_ (µM)	SCC-VII IC_50_ (µM)	SI ^a^
**2a ^b^**	17.5	9.8	1.8
**2b ^b^**	n.a.	n.a.	
**6**	20.3 ± 0.94	15.4 ± 0.53	1.3
**8**	18.8 ± 0.68	13.3 ± 0.20	1.4
**10**	8.0 ± 0.25	10.9 ± 0.03	0.7
**11**	12.1 ± 0.19	3.7 ± 0.39	3.3
**12**	15.5 ± 0.25	12.4 ± 0.21	1.3
**13**	13.5 ± 0.11	4.7 ± 0.24	2.9
**14**	18.8 ± 0.09	16.0 ± 0.37	1.2
**15**	13.7 ± 0.16	7.5 ± 0.12	1.8
**16**	17.7 ± 2.04	45.7 ± 0.54	0.4
**19**	n.a.^c^	n.a.	
**23**	n.a.	n.a.	
**27**	n.a.	n.a.	
**28**	29.4 ± 5.91	45.3 ± 6.21	0.6
**29ara**	n.a.	28.2 ± 8.40	
**29ribo**	n.a.	n.a.	
**30**	n.a.	n.a.	
5-FU**^d^**	-	60.7 ± 8.45	

^a^ selectivity index = HaCaT IC_50_/ SCC IC_50_; ^b^ data used from [18]; ^c^ n.a. = no cytotoxicity at the highest test concentration of 20 µg/mL; ^d^ **5-FU** (5-fluorouracil) used as a positive control.

**Table 3:** The effect of compounds **11**, **13** and **15** on the size of mother cells ^a^.

	Change in Cell Sizes before Division (%) ^b^		
	11	13	15
HaCaT n = 30	29.23 ± 0.60	−23.90 ± 0.83	−41.57 ± 0.61
SCC VII n = 30	−5.34 ± 0.35	23.36 ± 1.27	2.37 ± 0.76

^a^ Cells were treated with 3.7 µM of **11**, 4.7 µM of **13** and 7.5 µM of **15**, respectively; ^b^ (treated cell size/DMSO control cell size) × 100; cell size was calculated by the average values of n = 30 samples.

**Table 4:** The effect of compounds **11**, **13** and **15** on the duration of cell division of HaCaT and SCC cells ^a^.

	Difference in the Duration of Cell Division (min) Relative to the Control in (%) ^b^		
	11	13	15
HaCaT n = 30	−2.75 ± 15.62	6.25 ± 22.36	18.04 ± 20.45
SCC VII n = 30	8.00 ± 3.82	56.55 ± 20.70	−16.06 ± 5.92

^a^ Cells were treated with 3.7 µM of **11**, 4.7 µM of **13** and 7.5 µM of **15**, respectively; ^b^ (duration of cell division of treated cells/cell division duration of DMSO control cell division duration) × 100.

**Table 5:** The effect of compounds **11**, **13** and **15** on the generation times of HaCaT and SCC cells ^a^.

	Difference in the Duration of Cell Cycle (h) Change in % ^b^		
	11	13	15
HaCaT n = 30	29.10 ± 2.57	32.96 ± 3.00	27.41 ± 2.83
SCC VII n = 30	20.40 ± 2.97	12.86 ± 3.72	30.00 ± 3.06

^a^ Cells were treated with 3.7 µM of **11**, 4.7 µM of **13** and 7.5 µM of **15**, respectively; ^b^ (generation time of treated cells/generation time of DMSO control) × 100.

**Table 6:** In vitro inhibitory activity on coronavirus replication.

Compound	SARS-CoV-2 in VERO E6 Cells			HCoV-229E in HEL Cells	
	EC_50_ CPE (µM)	95% CI of EC_50_ CPE	CC_50_ (µM)	EC_50_ MTS (µM)	CC_50_ (µM)
**2a**	>35	n.a.	~35	n.d.	n.d.
**2b**	n.d.	n.d.	n.d.	7.4	14
**8**	n.d.	n.d.	n.d.	4.0	10
**10**	>16	n.a.	15	n.d.	n.d.
**11**	>6.4	n.a.	~6.4–16	4.1	10
**12**	~13	very wide	~18	n.d.	n.d.
**13**	>100	n.a.	>100	5.7	10
**15**	17	9.7–29	16.6	n.d.	n.d.
**16**	>100	n.a.	>100	>100	11
**17**	>100	n.a.	>100	4.9	9.2
**18**	n.d.	n.d.	n.d.	>100	10
**19**	>100	n.a.	>100	>100	>100
**28**	>100	n.a.	>100	>100	>100
**29ara**	n.d.	n.d.	n.d.	>100	>100
**29ribo**	n.d.	n.d.	n.d.	>100	>100
**30**	>100	n.a.	>100	>100	>100
Remdesivir	2.2	1.7 to 2.8	>100	n.d.	n.d.

n.d. not determined, n.a. not applicable.

## Data Availability

The datasets used and/or analyzed during the current study are available from the corresponding author (A.B.) on reasonable request.

## Supplementary Materials

The supporting information can be downloaded at: https://www.mdpi.com/article/10.3390/ijms232012566/s1.
