# Supplementary material for: Synthesis and Anticancer and Antiviral Activities of C-2′-Branched Arabinonucleosides
Source: Int J Mol Sci. 2022 Oct 19;23(20):12566. doi: 10.3390/ijms232012566 (PMC9603951; doi:10.3390/ijms232012566)

Supplementary Materials

Synthesis and anticancer and antiviral activities of C-2'-branched arabinonucleosides

Miklós Bege, Alexandra Kiss, Ilona Bereczki, Jan Hodek, Lenke Polyák, Gábor Szemán-Nagy, Lieve Naesens, Jan Weber, Anikó Borbás

Table of contents

General information S2

Synthesis of furanosyl exomethylenes S2

Synthesis of 5'-thiouridine S9

Characteristic NMR signals of the compounds S10

Cytotoxic activity of compounds **6, 8, 10, 12, 14, 16, 28** and **29ara** S12

Anti-SARS-CoV-2 activity determination in VERO E6 cells S14

Cytotoxicity in VERO E6 cells S15

References S15

NMR spectra of the compounds S17

General information

Compounds **9f**[1] and **9g**[1] were prepared according to literature procedures. 2,2-Dimethoxy-2-phenylacetophenone (DPAP), 4-methoxyacetophenone (MAP), thioacetic acid, *n*-propyl-mercaptane, *n*-butyl-mercaptane and *n*-hexyl-mercaptane were purchased from Sigma-Aldrich Chemical Co. and used without further purification. Optical rotations were measured at room temperature with a Perkin-Elmer 241 automatic polarimeter. TLC was performed on Kieselgel 60 F_{254} (Merck) with detection by UV-light (254 nm) and immersing into sulfuric acidic ammonium molybdate solution or 5% ethanolic sulfuric acid followed by heating. Flash column chromatography was performed on silica gel 60 (Merck, 0.040-0.063 mm). Organic solutions were dried over anhydrous Na_2SO_4 and concentrated in vacuum. The ^{1}H NMR (360, 400 and 500 MHz) and ^{13}C NMR (90, 100 and 125 MHz) spectra were recorded with Bruker DRX-360, Bruker DRX-400 and Bruker Avance II 500 spectrometers at 25 °C. Chemical shifts are referenced to Me_4Si (0.00 ppm for ^{1}H) and to the residual solvent signals ($CDCl_3$: 77.2, DMSO-d_6: 39.5, CD_3OD: 49.0 for ^{13}C). Two-dimensional COSY and ^{1}H–^{13}C HSQC experiments were used to assist NMR assignments. MALDI-TOF MS analyses of the compounds were carried out in the positive reflectron mode using a BIFLEX III mass spectrometer or with a Bruker Autoflex Speed mass spectrometer equipped with a time-of-flight (TOF) mass analyzer. 2,5-Dihydroxybenzoic acid (DHB) was used as matrix and $F_3CCOONa$ as cationising agent in DMF.

The photoinitiated reactions were carried out in a borosilicate vessel by irradiation with a low-pressure Hg-lamp (Osram Supratec UV, HTC 150-211, 150 W, 230 V, R7s) giving maximum emission at 365 nm, without any caution to exclude air or moisture.

Synthesis of furanosyl exomethylenes

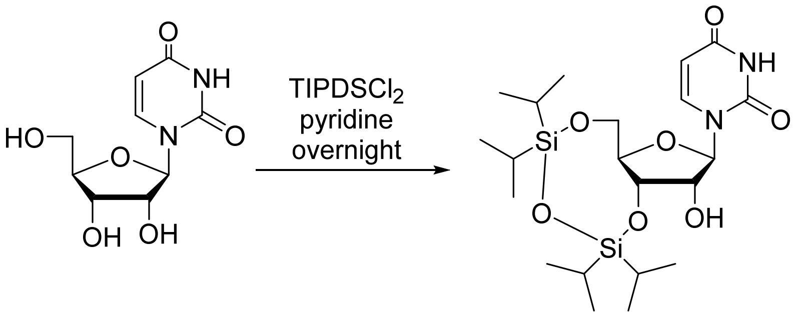

3 **5**

3’,5’-*O*-(1,1,3,3-Tetraisopropyldisiloxane-1,3-diyl)-β-D-ribofuranosyl-uracil (5)[2]

Uridine (1.68 g, 6.87 mmol) was dissolved in dry pyridine (20 ml). 1,3-dichloro-1,1,3,3-tetraisopropyldisiloxane (2.3 ml, 7.2 mmol, 1.05 equiv.) was added and the reaction mixture was stirred overnight. Next day, water (10 ml) was added and the solvent was evaporated under reduced pressure. The residue was dissolved in EtOAc (100 ml) and extracted with H_2O (2x), 10% aq. solution of $NaHSO_4$ (2x) H_2O (1x) and brine (1x). The organic phase was dried over Na_2SO_4, filtered and concentrated *in vacuo*. The crude product was purified by flash column chromatography (hexane/acetone 8/2→7/3) to give compound **5** (2.54 g, 76%) as a white solid. $[\alpha]_D$ = +10.8 (c = 0.12, $CHCl_3$), Rf = 0.15 (hexane/acetone 8/2), ^1H NMR (500 MHz, $CDCl_3$) δ (ppm) 9.98 (s, 1H, N*H*), 7.80 (d, *J* = 8.1 Hz, 1H, H-6), 5.75 (s, 1H, H-1'), 5.71 (d, *J* = 8.1 Hz, 1H, H-5), 4.31 (dd, *J* = 8.8, 4.6 Hz, 1H), 4.20 (dd, *J* = 16.4, 12.1 Hz, 3H), 4.01 (dd, *J* = 13.2, 2.2 Hz, 1H), 3.87 (br s, 1H), 1.12 – 1.00 (m, 28H, 8x *i*-PrCH_3 and 4x *i*-PrC*H*). ^{13}C NMR (125 MHz, $CDCl_3$) δ (ppm) 163.8, 150.5 (2C, 2x*C*O), 140.0 (1C, C-6), 102.1 (1C, C-5), 91.1, 82.0, 75.2, 68.8 (4C, C-1', C-2', C-3', C-4'), 60.2 (1C, C-5'), 17.6, 17.5, 17.4, 17.3, 17.1, 17.0, 17.0, 16.9 (8C, 8x *i*-Pr*C*H_3), 13.5, 13.1, 13.0, 12.6 (4C, 4x *i*-Pr*C*H). MALDI-ToF MS *m/z* calcd for $C_{21}H_{38}N_2O_7Si_2Na^+$: 509.212 $[M+Na]^+$, found 509.167.

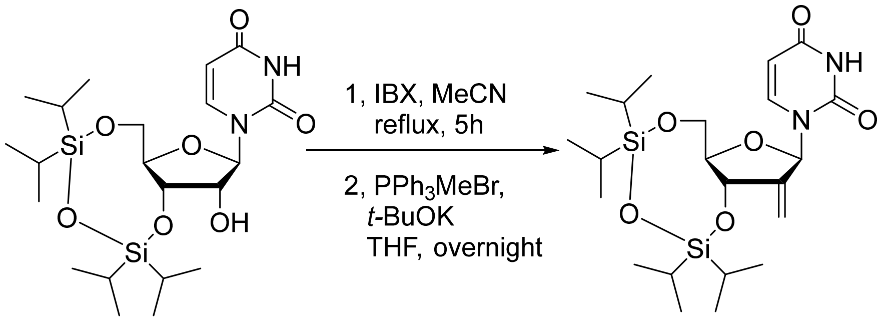

5 **7**

2'-Deoxy-2'-methylene-3',5'-*O*-(1,1,3,3-tetraisopropyldisiloxane-1,3-diyl)-β-D-ribofuranosyl-uracil (7)[3]

Compound **5** (1.54 g, 3.17 mmol) and IBX (2.67 g, 9.52 mmol, 3.0 equiv.) were suspended in MeCN (25 ml) and stirred at 100 °C for 5 h. After 5 h, the reaction mixture was diluted with EtOAc (100 ml), filtered and evaporated under reduced pressure.

Methyltriphenylphosphonium-bromide (2.8 g, 7.92 mmol, 2.5 equiv) was suspended in dry THF (20 ml) under Ar. T-BuOK (0.89 g, 7.92 mmol, 2.5 equiv) was added and stirred for 2 h at room temperature. After 2 h, the reaction mixture was cooled to -78 °C, and the keton crude product was added to this suspension. The reaction mixture was was allowed to warm to -10 °C

over 1 h and stored at 0−4 °C overnight. The reaction mixture was diluted with CH_2Cl_2 (200 ml) and washed with saturated aq. NH_4Cl solution (3 x 50 ml) and brine (1 x 50ml). The organic phase was dried over anhydrous Na_2SO_4 and concentrated in *vacuo*. The residue was purified by flash column chromatography (gradient elution hexane/acetone 9/1→85/15) to yield compound **7** (641 mg, 43%) as a yellow syrup.

$[\alpha]_D$ = -9.6 (c = 0.30, $CHCl_3$), Rf = 0.13 (hexane/acetone 8/1), ^{1}H NMR (360 MHz, $CDCl_3$) δ (ppm) 10.18 (s, 1H, N*H*), 7.49 (d, *J* = 8.1 Hz, 1H, H-6), 6.53 (s, 1H, H-1'), 5.74 (d, *J* = 8.1 Hz, 1H, H-5), 5.56 (s, 1H, exomethylene H_a), 5.45 (s, 1H, exomethylene H_b), 4.82 (d, *J* = 8.6 Hz, 1H, H-3'), 4.16 (dd, *J* = 13.1, 1.4 Hz, 1H, H-5'a), 4.05 (dd, *J* = 13.2, 2.4 Hz, 1H, H-5'b), 3.70 (d, *J* = 8.8 Hz, 1H, H-4'), 1.07 (m, 28H, 8x *i*-PrCH_3 and 4x *i*-PrC*H*). ^{13}C NMR (90 MHz, $CDCl_3$) δ (ppm) 164.0 (1C, C-4), 150.8 (1C, C-2), 146.6 (1C, C-2'), 139.9 (1C, C-6), 112.2 (1C, exomethylene*C*H_2), 102.7 (1C, C-5), 83.9 (1C, C-1'), 83.1 (1C, C-4'), 69.8 (1C, C-3',), 60.4 (1C, C-5'), 17.5, 17.4, 17.3, 17.0, 17.0, 16.9, 16.9 (8C, 8x *i*-Pr*C*H_3), 13.6, 13.0, 12.9, 12.5 (4C, 4x *i*-Pr*C*H). MALDI-ToF MS: *m/z* calcd for $C_{22}H_{38}N_2O_6Si_2Na^+$: 505.217 $[M+Na]^+$, found 505.182.

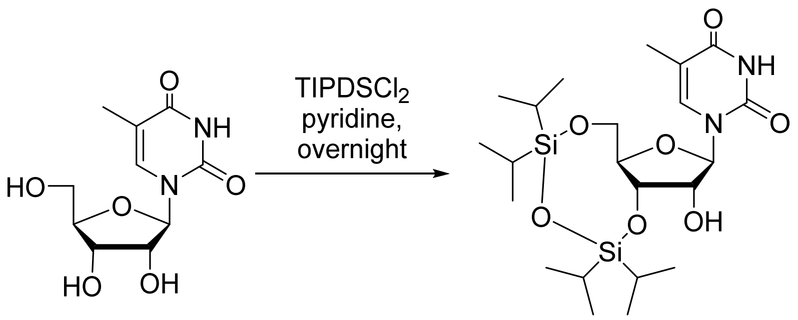

4 **6**

3',5'-*O*-(1,1,3,3-Tetraisopropyldisiloxane-1,3-diyl)-β-D-ribofuranosyl-thymine (6)[4]

Ribothymidine (3.54 g, 13.74 mmol) was dissolved in dry pyridine (40 ml). 1,3-dichloro-1,1,3,3-tetraisopropyldisiloxane (4.6 ml, 14.4 mmol, 1.05 equiv.) was added and the reaction mixture was stirred overnight. Next day, water (20 ml) was added and the solvent was evaporated under reduced pressure. The residue was dissolved in EtOAc (400 ml) and extracted with, 10% aq. solution of $NaHSO_4$ (2x) and brine (1x). The organic phase was dried over Na_2SO_4, filtered and concentrated *in vacuo*. The crude product was purified by flash column chromatography (hexane/acetone 8/2→7/3) to give compound **6** (5.81 g, 84%) as a white foam.

$[\alpha]_D$ = -15.0 (c = 0.18, $CHCl_3$), Rf = 0.26 (hexane/acetone 8/2), ^{1}H NMR (360 MHz, $CDCl_3$) δ (ppm) 10.22 (s, 1H, N*H*), 7.56 (s, 1H, H-6), 5.73 (s, 1H, H-1'), 4.37 – 4.27 (m, 2H), 4.26 – 4.18 (m, 3H), 4.01 (dd, *J* = 13.4, 2.6 Hz, 1H, H-5'a), 1.91 (s, 3H, thymineCH_3), 1.12 – 0.96 (m, 28H,

6x*i*-PrCH_3 & 4x*i*-Pr*CH*). ^{13}C NMR (90 MHz, $CDCl_3$) δ (ppm) 164.4, 150.6 (2C, 2x*C*O), 135.6 (1C, C-6), 110.6 (1C, C-5), 91.3, 81.9, 75.1, 68.7 (4C, C-1', C-2', C-3', C-4'), 60.1 (1C, C-5'), 17.5, 17.4, 17.3, 17.1, 17.0, 16.9 (8C, 8x *i*-PrCH_3), 13.5, 13.0, 12.8, 12.7, 12.5 (5C, 4x *i*-Pr*C*H & thymine *C*H_3). MALDI-ToF MS: *m/z* calcd for $C_{22}H_{40}N_2O_7Si_2Na^+$: 523.227 $[M+Na]^+$, found 523.172.

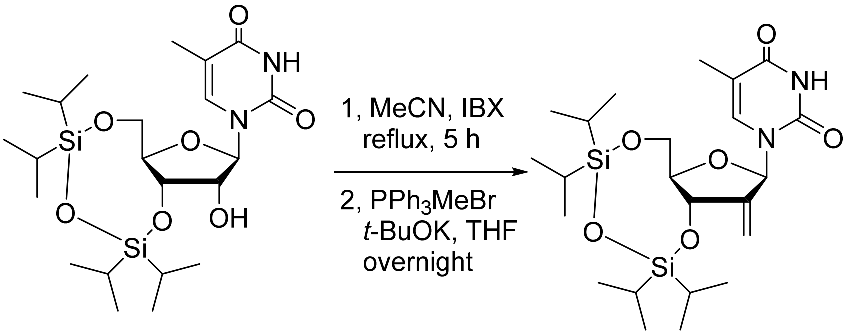

6 **8**

2'-Deoxy-2'-methylene-3',5'-*O*-(1,1,3,3-tetraisopropyldisiloxane-1,3-diyl)-β-D-ribofuranosyl-thymine (8)[3]

Compound **6** (5.5 g, 10.9 mmol) and IBX (9.2 g, 32.9 mmol, 3.0 equiv.) were suspended in MeCN (40 ml) and stirred for 5 h at 100 °C. The suspension was diluted with EtOAc (200 ml), filtered, and concentrated under reduced pressure.

Methyltriphenylphosphonium bromide (11.8 g, 32.9 mmol, 3.0 equiv.) was suspended in dry THF (30 ml). Under Ar *t*-BuOK (3.7 g, 32.9 mmol, 3.0 equiv.) was added and stirred for 2 h. After 2 h, the reaction mixture was cooled to -78 °C, and the keton crude product (dissolved in dry THF) was added. The reaction mixture was was allowed to warm to −10 °C over 1 h and stored at 0−4 °C overnight. The reaction mixture was diluted with CH_2Cl_2 (350 ml) and washed with saturated aq. NH_4Cl solution (3 x 10 ml) and brine (1 x 10ml). The organic phase was dried over anhydrous Na_2SO_4 and concentrated in *vacuo*. The residue was purified by flash column chromatography (gradient elution hexane/acetone 9/1→85/15→8/2) to yield compound **8** (2.7 g, 48%) as a yellow syrup.

$[\alpha]_D$ = -36.1 (c = 0.18, $CHCl_3$), Rf = 0.27 (hexane/acetone 8/2), ^{1}H NMR (400 MHz, $CDCl_3$) δ (ppm) 9.49 (s, 1H, NH), 7.12 (s, 1H, H-6), 6.58 (d, *J* = 1.5 Hz, 1H, H-1'), 5.49 (dd, *J* = 2.6, 1.3 Hz, 1H, exomethyleneH_a), 5.48 – 5.45 (m, 1H, exomethyleneH_b), 4.86 (dd, *J* = 8.8, 1.7 Hz, 1H, H-3'), 4.15 (dd, *J* = 13.3, 2.0 Hz, 1H, H-5'$_a$), 4.05 (dd, *J* = 13.2, 2.8 Hz, 1H, H-5'$_b$), 3.67 (dt, *J* = 8.8, 2.4 Hz, 1H, H-4'), 1.89 (s, 3H, thymineCH_3), 1.08 (dd, *J* = 19.6, 4.7 Hz, 28H, 28H, 8x *i*-

PrCH_3 and 4x *i*-Pr*CH*). ^{13}C NMR (100 MHz, $CDCl_3$) δ (ppm) 164.0, 150.9 (2C, 2x*C*O), 147.1 (1C, C-2'), 135.6 (1C, C-6), 112.1, 111.5 (2C, exomethyleneCH_2 & C-5), 83.5, 82.8, 70.2 (3C, C-1', C-4', C-3'), 60.2 (1C, C-5'), 17.5, 17.3, 17.1, 17.1, 17.1, 16.9 (8C, 8x *i*-Pr*C*H_3), 13.7, 13.1, 12.7, 12.6, 12.6 (5C, 4x4x *i*-Pr*C*H & thymine*C*H_3). MALDI-ToF MS: *m/z* calcd for $C_{23}H_{40}N_2O_6Si_2Na^+$: 519.232 $[M+Na]^+$, found 519.171.

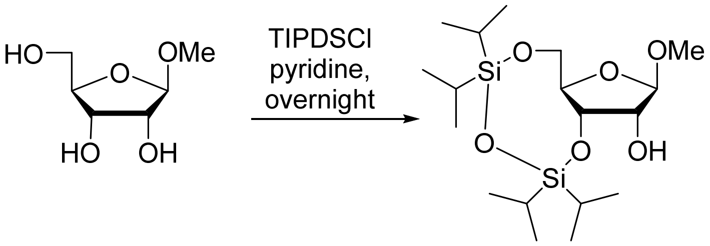

20 **21**

Methyl 3,5-*O*-(1,1,3,3-tetraisopropyldisiloxane-1,3-diyl)-β-D-ribofuranoside (21)[5]

Methyl-β-D-ribofuranoside (5.0 g, 30.5 mmol) was dissolved in dry Pyridine (50 mL), and cooled to 0 °C. Then, 1,3-dichloro-1,1,3,3-tetraisopropyldisiloxane (10 mL, 32 mmol, 1.05 equiv.) was added and stirred overnight at r.t. Next day, water (40 mL, was added and the solvent was evaporated under reduced pressure. The residue was dissolved in EtOAc, and extracted with 10% aq.$NaHSO_4$ and brine. The organic phase was dried over Na_2SO_4, filtered and concentrated *in vacuo*. The crude product was purified by flash chromatography (hexane/acetone 98/2) to give compound **21** (9.4 g, 84%).

Rf= 0.68 (hexane/acetone 9/1), ^{1}H NMR (360 MHz, $CDCl_3$) δ (ppm) 4.82 (s, 1H, H-1), 4.50 (t, *J* = 5.4 Hz, 1H), 4.09 – 3.99 (m, 3H), 3.75 (dd, *J* = 10.5, 8.8 Hz, 1H, H-5b), 3.32 (s, 3H, OMe), 2.99 (s, 1H), 1.15 – 0.97 (m, 28H, 8x*i*-PrCH_3 & 4x *i*-Pr*CH*). ^{13}C NMR (90 MHz, $CDCl_3$) δ (ppm)107.4 (1C, C-1), 82.8, 75.9, 75.2 (3C, C-2, C-3, C-4), 66.3 (1C, C-5), 55.0 (1C, OMe), 17.6, 17.5, 17.3, 17.1 (8C, 8x*i*-Pr*C*H_3), 13.4, 12.9, 12.7 (4C, 4x*i*-Pr*C*H).

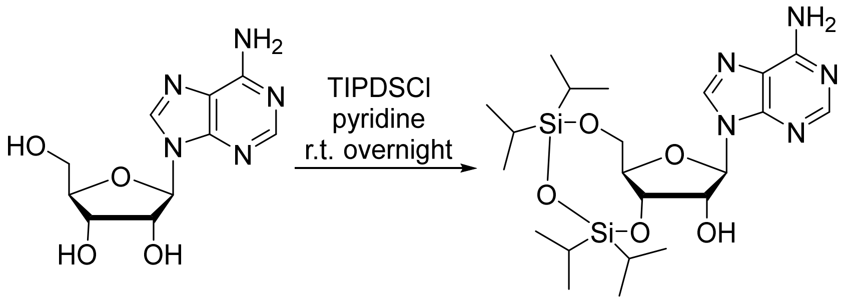

24 **25**

3′,5′-*O*-(1,1,3,3-Tetraisopropyldisiloxyl)-adenosine (25)[6]

Adenosine (2.0 g, 7.5 mmol) was dissolved in dry pyridine (50 mL) and cooled to 0 ºC. $TIPDSCl_2$ (2.5 mL, 7.85 mmol, 1.05 equiv.) was added, the reaction mixture was allowed to warm up to room temperature and stirred for 2 days. The solvent was evaporated under reduced pressure, the residue was dissolved in EtOAc (500 mL) and washed with water (100 mL), 10% aq. $NaHSO_4$ solution (3x100 mL) and brine (100 mL). The organic phase was dried over Na_2SO_4, filtered and evaporated under reduced pressure. The crude product was purified by flash column chromatography (CH_2Cl_2/MeOH 95/5) to give compound **25** (3.52 g, 93%) as white solid.

$[\alpha]_D$= +10.8 (c= 0.12, DMSO), Rf = 0.32 (CH_2Cl_2/MeOH 95/5), ^{1}H NMR (400 MHz, DMSO) δ (ppm) 8.22, 8.08 (2xs, 2x1H, H-2, H-8), 7.34 (s, 2H, NH_2), 5.88 (s, 1H), 5.65 (d, J = 4.6 Hz, 1H, H-1'), 4.80 (dd, J = 8.5, 5.1 Hz, 1H, H-3'), 4.52 (t, J = 4.6 Hz, 1H, H-2'), 4.07 (dd, J = 12.6, 3.2 Hz, 1H, H-5'a), 4.01 (dt, J = 8.5, 2.8 Hz, 1H, H-4'), 3.93 (dd, J = 12.6, 2.6 Hz, 1H, H-5'b), 1.04 (s, 28H, 4x*i*-PrC*H*, 8x*i*-PrCH_3). ^{13}C NMR (100 MHz, DMSO) δ (ppm) 156.1, 148.6, 119.3 (3C, 3x adenine C_q), 152.5 (1C, adenine *C*H), 89.4, 80.8, 73.7, 69.8 (4C, C-1', C-2', C-3', C-4'), 60.8 (1C, C-5'), 17.4, 17.2, 17.0, 16.9, 16.8 (8C, 8x *i*-Pr*C*H_3), 12.8, 12.5, 12.3, 12.1 (4C, 4x *i*-Pr*C*H). MALDI-ToF MS: *m/z* calcd for $C_{22}H_{39}N_5O_5Si_2Na^+$: 532.239 $[M+Na]^+$, found 532.279.

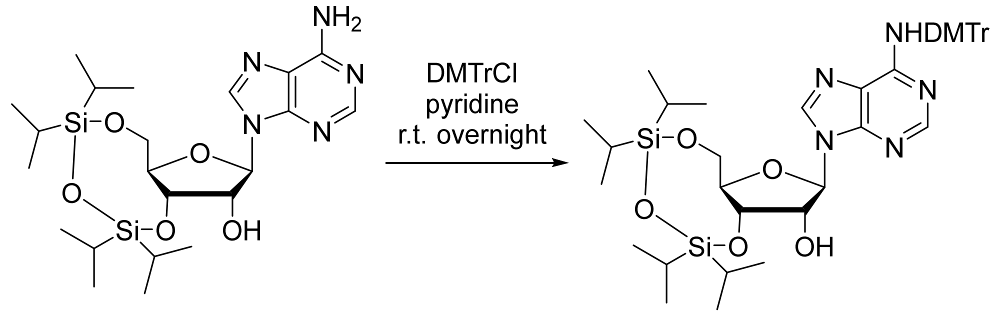

25 26

3′,5′-*O*-(1,1,3,3-Tetraisopropyldisiloxyl)-*N*-dimethoxytrityl-adenosine (26)[7]

Compound **25** (410 mg, 0.80 mmol) was dissolved in dry pyridine (5 mL) and DMTrCl (553 mg, 1.6 mmol, 2.0 equiv.) was added, the reaction mixture was stirred at r.t. overnight. Next day, the was diluted with CH_2Cl_2 (200 mL) and washed with 10% aq. $NaHSO_4$ solution. The organic phase was dried over Na_2SO_4, filtered and evaporated under reduced pressure. The crude product was purified by flash column chromatography (hexane/acetone 9/1→8/2) to give compound **26** (498 mg, 77%) as white foam.

$[\alpha]_D$= -35.7 (c = 0.21, $CHCl_3$), Rf = 0.54 (hexane/acetone 6/4), ^{1}H NMR (400 MHz, $CDCl_3$) δ (ppm) 7.99, 7.86 (2xs, 2x1H, H-2, H-8), 7.33 (dd, *J* = 8.2, 1.3 Hz, 2H, arom), 7.28 – 7.20 (m, 7H, arom), 6.91 (s, 1H, N*H*), 6.82 – 6.75 (m, 4H, arom), 5.89 (d, *J* = 1.0 Hz, 1H, H-1’), 5.15 (dd, *J* = 7.6, 5.6 Hz, 1H, H-3’), 4.61 (d, *J* = 5.5 Hz, 1H, H-2’), 4.12 (dd, *J* = 11.6, 4.1 Hz, 1H, H-5’a), 4.07 (dd, *J* = 7.7, 3.0 Hz, 1H, H-4’), 4.05 – 4.00 (m, 1H, H-5’b), 3.77 (s, 6H, 2x OMe), 3.52 (s, 1H, O*H*) 1.12 – 1.03 (m, 28H, 4x*i*-PrC*H*, 8x*i*-PrCH_3). ^{13}C NMR (100 MHz, $CDCl_3$) δ (ppm) 158.4 (2C, 2x arom C_q), 152.4 (1C, adenine *C*H), 154.3, 148.1 (2C, 2x adenine C_q), 145.5 (1C, Trt C_q), 137.6 (2C, 2x Trt C_q), 130.2, 128.9, 127.9, 126.9, 113.2 (13C, arom.), 121.7 (1C, adenine C_q), 89.9, 82.2, 75.0, 70.9 (4C, C-1’, C-2’, C-3’, C-4’), 70.7 (1C, Trt C_q), 61.8 (1C, C-5’), 55.3 (2C, 2x OMe), 17.6, 17.4, 17.2, 17.1 (8C, 8x *i*-PrCH_3), 13.4, 13.2, 12.8, 12.7 (4C, 4x *i*-Pr*C*H). MALDI-ToF MS: *m/z* calcd for $C_{43}H_{57}N_5O_7Si_2Na^+$: 834.369 $[M+Na]^+$, found 834.464.

Synthesis of 5'-thiouridine

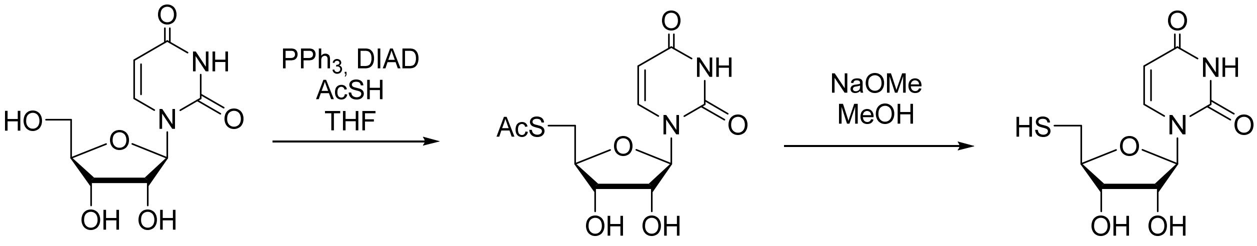

9e

5'-S-acetyl-5'-thiouridine

PPh_3 (6.5 g, 24.6 mmol, 2.0 equiv.) was suspended in dry THF (50 mL). The reaction mixture was cooled to 0 °C and diisopropyl azodicarboxylate (DIAD) (5.0 mL, 24.6 mmol, 2.0 equiv.) was added dropwise and stirred for 30 min at 0 °C. Uridine (3.02 g, 12.3 mmol) and AcSH (1.8 mL, 24.6 mmol, 2.0 equiv.) were dissolved in dry DMF (50 mL) and the solution was added to the reaction mixture dropwise, then stirred for 30 min at 0 °C and for 30 min at r.t. The solvent was evaporated under reduced pressure and the crude product was purified by flash chromatography (CH_2Cl_2/MeOH 97.5/2.5→95/5→9/1→8/2) to give **5'-S-acetyl-5'-thiouridine** (2.5 g, 70%) as a yellowish solid.

$[\alpha]_D$= +17.8 (c = 0.23, DMSO), Rf = 0.16 (CH_2Cl_2/MeOH 95/5), ^{1}H NMR (400 MHz, DMSO) δ (ppm) 11.36 (s, 1H, N*H*), 7.63 (d, *J* = 8.1 Hz, 1H, H-6), 5.73 (d, *J* = 5.6 Hz, 1H, H-1'), 5.67 (dd, *J* = 8.0, 2.1 Hz, 1H, H-5), 5.43 (d, *J* = 5.6 Hz, 1H, 2'-O*H*), 5.29 (d, *J* = 4.5 Hz, 1H, 3'-O*H*), 4.14 (dd, *J* = 10.2, 5.1 Hz, 1H, H-2'), 3.87 – 3.78 (m, 2H, H-4', H-3'), 3.27 (dd, *J* = 13.8, 5.0 Hz, 1H, H-5'a), 3.10 (dd, *J* = 13.8, 7.0 Hz, 1H, H-5'b), 2.36 (s, 3H, AcCH_3). ^{13}C NMR (100 MHz, DMSO) δ (ppm) 194.8 (1C, Ac*C*O), 163.1, 150.7 (2C, C-2, C-4), 141.2 (1C, C-6), 102.2 (1C, C-5), 88.4, 82.3, 72.3, 72.2 (4C, C-1', C-2', C-3', C-4'), 31.0 (1C, C-5'), 30.5 (1C, AcCH_3). MALDI-ToF MS: *m/z* calcd for $C_{11}H_{14}N_2O_6SNa^+$: 325.0470 $[M+Na]^+$, found 325.0423.

5'-thiouridine (9e)[8]

5'-S-acetyl-5'-thiouridine (2.4 g, 8.0 mmol) was dissolved in dry MeOH (50 mL) under Ar, and NaOMe (648 mg, 12 mmol, 1.5 equiv.) was added and stirred at r.t. for 1 h. The reaction mixture was neutralized with Amberlite IR 120 ion exchanger resine, filtered off, and evaporated under reduced pressure. The crude product was purified by flash column chromatography (CH_2Cl_2/MeOH 9/1) to give compound **9e** (1.47 g, 71%) as a white solid

$[\alpha]_D$= -40.8 (c = 0.13, DMSO), Rf = 0.24(CH_2Cl_2/MeOH 95/5), ^{1}H NMR (400 MHz, DMSO) δ (ppm) 11.35 (s, 1H, N*H*), 7.67 (d, *J* = 8.1 Hz, 1H, H-6), 5.78 (d, *J* = 6.0 Hz, 1H, H-1'), 5.67 (d, *J* = 8.1 Hz, 1H, H-6), 5.41 (d, *J* = 5.8 Hz, 1H, H-3'), 5.22 (d, *J* = 5.0 Hz, 1H), 4.13 (dd, *J* = 11.3, 5.7 Hz, 1H, H-2'), 3.96 – 3.90 (m, 1H), 3.85 (dd, *J* = 9.5, 5.8 Hz, 1H, H-4'), 2.81 (dd, *J* = 13.6, 5.5 Hz, 1H, H-5'a), 2.73 (dd, *J* = 13.8, 6.0 Hz, 1H, H-5'b), 2.46 (s, 1H). ^{13}C NMR (100 MHz, DMSO) δ (ppm) 163.1, 150.8 (2C, C-2, C-4), 141.2 (1C, C-6), 102.2 (1C, C-5), 88.0, 84.6, 72.4, 71.5 (4C, C-1', C-2', C-3', C-4'), 26.3 (1C, C-5'). MALDI-ToF MS: *m/z* calcd for $C_9H_{12}N_2O_5SNa^+$: 283.0365 [M+Na]$^+$, found 283.0306.

Characteristic NMR signals of the compounds

Table S1. Characteristic NMR signals of ribonucleoside (**5, 6, 21, 25, 26** and **29ribo**) and arabinonucleoside (**10-16, 18, 23, 28, 29arabino** and **30**) derivatives

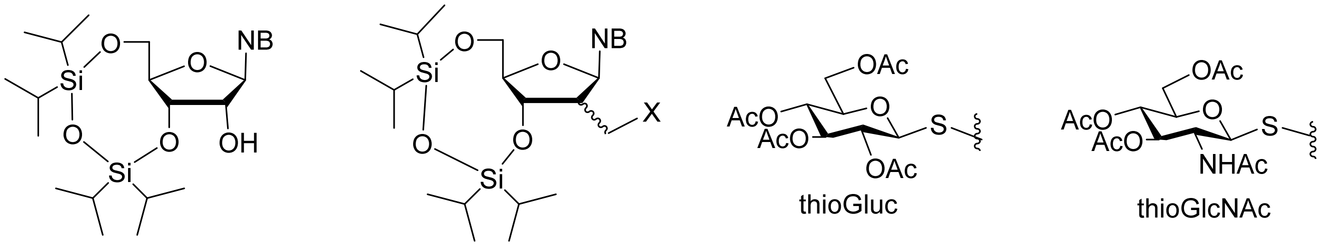

5, 6, 21, 25, 26   **10-16, 18, 23, 28, 29, 30**

Product	NB	X	H-1'	H-2'	H-3'	H-4'	C-1'	C-2'	C-3'	C-4'
**5**	U	-	5.75 s	N.A.	N.A.	N.A.	91.1	N.A.	N.A.	N.A.
**6**	T	-	5.73 s	N.A.	N.A.	N.A.	91.3	N.A.	N.A.	N.A.
**10**	U	-SPr	6.23 d J= 4.7 Hz	2.86 m[a]	4.35 t J= 8.8 Hz	3.75 d J= 8.1 Hz	84.2	49.3	N.A.	84.2
**11**	U	-SBu	6.23 d J= 4.4 Hz	2.89-2.81 m[a]	4.35 t J= 8.8 Hz	3.75 d J= 8.1 Hz	84.2	49.3	N.A.	84.2
**12**	U	-SHex	6.24 d J= 5.2 Hz	2.91-2.83 m[a]	4.36 t J= 8.8 Hz	3.77 dt J= 8.1, 2.2 Hz	84.3	49.4	N.A.	84.3

**13**	U	-SAc	6.26 d J= 6.2 Hz	2.90-2.78 m[a]	4.24 t J= 8.9 Hz	3.77 d J= 8.2 Hz	84.2	49.7	N.A.	84.2
**16**	T	-5'-thiouridine	6.23 s	3.00-2.91 m	4.47 s	3.89-3.81 m	N.A.	50.7	74.5	85.2
**14**	T	-SPr	6.24 d J= 4.1 Hz	2.82 d J= 4.1 Hz	4.38 t J= 8.6 Hz	3.76 d J= 7.9 Hz	84.1	49.4	71.3	84.1
**15**	T	-SBu	6.12 s	2.84-2.79 m[a]	4.35 t J= 8.3 Hz	3.73 d J= 7.9 Hz	84.3	49.3	N.A.	84.0
**18 major**	T	-thioGlcNAc	6.36 d J= 7.2 Hz	N.A.	N.A.	N.A.	84.1	N.A.	N.A.	N.A.
**18minor**	T	-thioGlcNAc	5.85 s	N.A.	N.A.	N.A.	89.2	N.A.	N.A.	N.A.
**21**	OMe	-	4.82 s	N.A.	N.A.	N.A.	107.4	N.A.	N.A.	N.A.
**23**	OMe	-SBu	4.88 d J= 3.9 Hz	2.44 td J= 9.4, 4.6 Hz	4.19 dd J= 8.8, 5.5 Hz	3.96-3.89 m	103.8	53.6	N.A.	N.A.
**25**	A	-	5.65 d J= 4.6 Hz	4.52 t J= 4.6 Hz	4.80 dd J= 8.5, 5.1 Hz	4.01 dt J= 8.5, 2.8 Hz	89.4	N.A.	N.A.	N.A.
**26**	A^{DMTr}	-	5.89 d J= 1.0 Hz	4.61 d J= 5.5 Hz	5.15 dd J= 7.6, 5.6 Hz	4.07 dd J= 7.7, 3.0 Hz	89.9			
**28**	A^{DMTr}	-SBu	6.25 d J= 7.6 Hz	2.96 ddd J= 13.5, 11.2, 4.0 Hz	5.14-5.05 m	3.89 t J= 6.7, 3.0 Hz	85.7	51.4	74.9	85.0
**30**	A^{DMTr}	-thioGluc	6.25 d J= 7.5 Hz	3.23-3.12 m	5.16-5.08	3.89 t J= 6.7, 3.0 Hz	85.5	52.3	75.3	82.6

**29 ribo**	A^{DMTr}	-SAc	5.89 s	2.78 d J= 14.3 Hz	5.12 d J= 7.6 Hz	4.09 ddd 7.7, 6.0, 3.1 Hz	90.8	55.4	75.3	82.6
**29 arabino**	A^{DMTr}	-SAc	6.25 d, 7.5 Hz	3.02-2.90 m	4.99-4.91 m	3.87 ddd 7.9, 4.9, 3.3 Hz	84.9	51.1	74.2	84.7

a: overlapping signals
N.A.: not assigned

Cytotoxic activity of compounds **6, 8, 10, 12, 14, 16, 28** and **29ara**

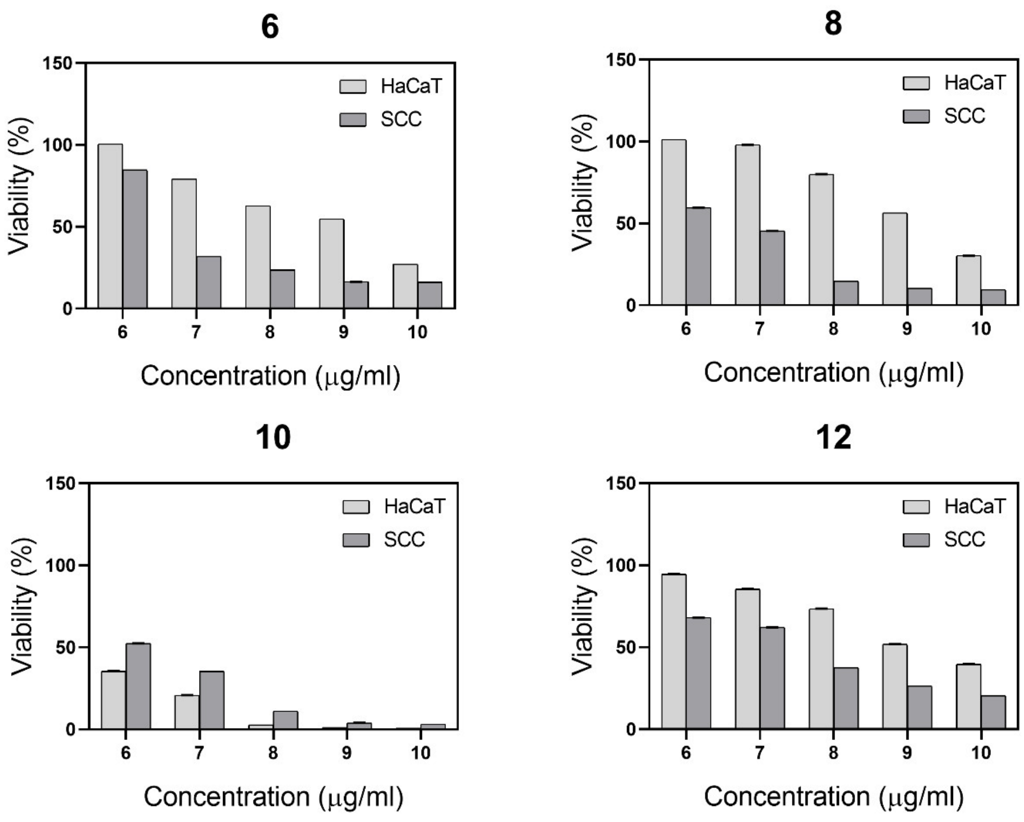

Figure S1. Effect of compounds **6, 8, 10** and **12** on viability of HaCaT and SCC cells by MTT assay

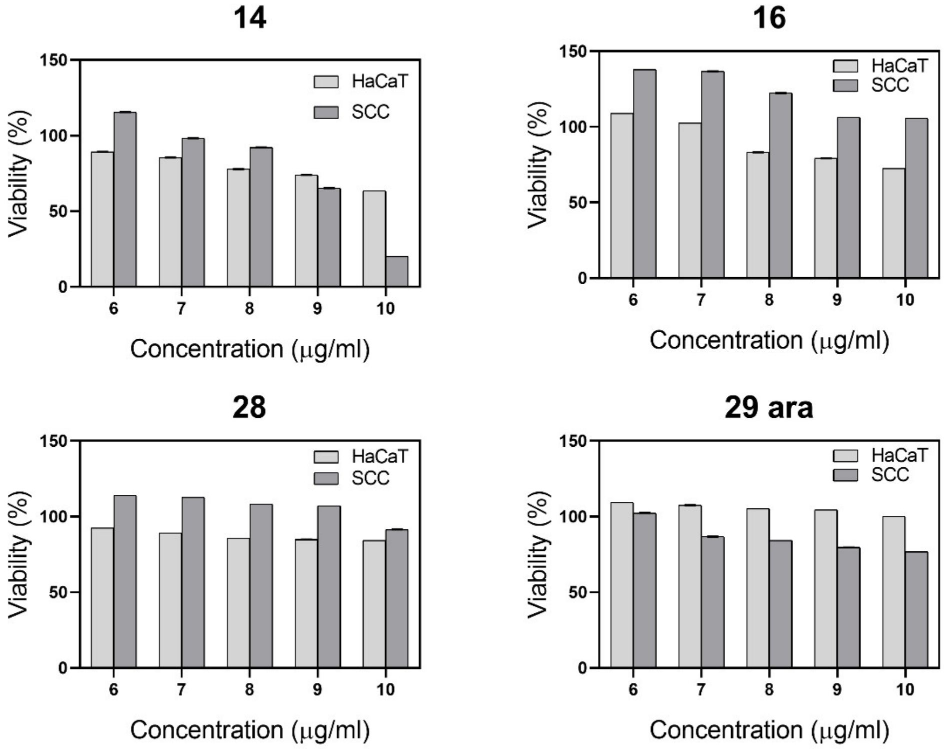

Figure S2. Effect of compounds **14, 16, 28** and **29ara** on viability of HaCaT and SCC cells by MTT assay

Anti-SARS-CoV-2 activity determination in VERO E6 cells

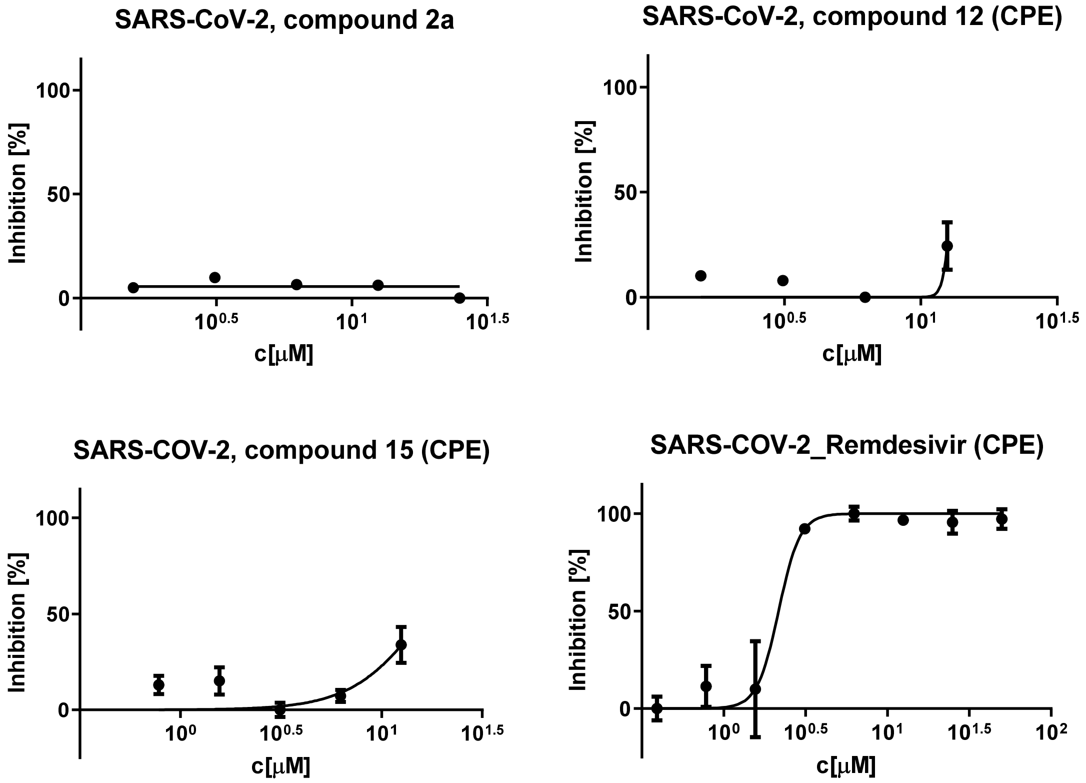

Figure S3. Inhibition of SARS-CoV-2-induced cytopathic effect in Vero E6 cells

Cytotoxicity in VERO E6 cells

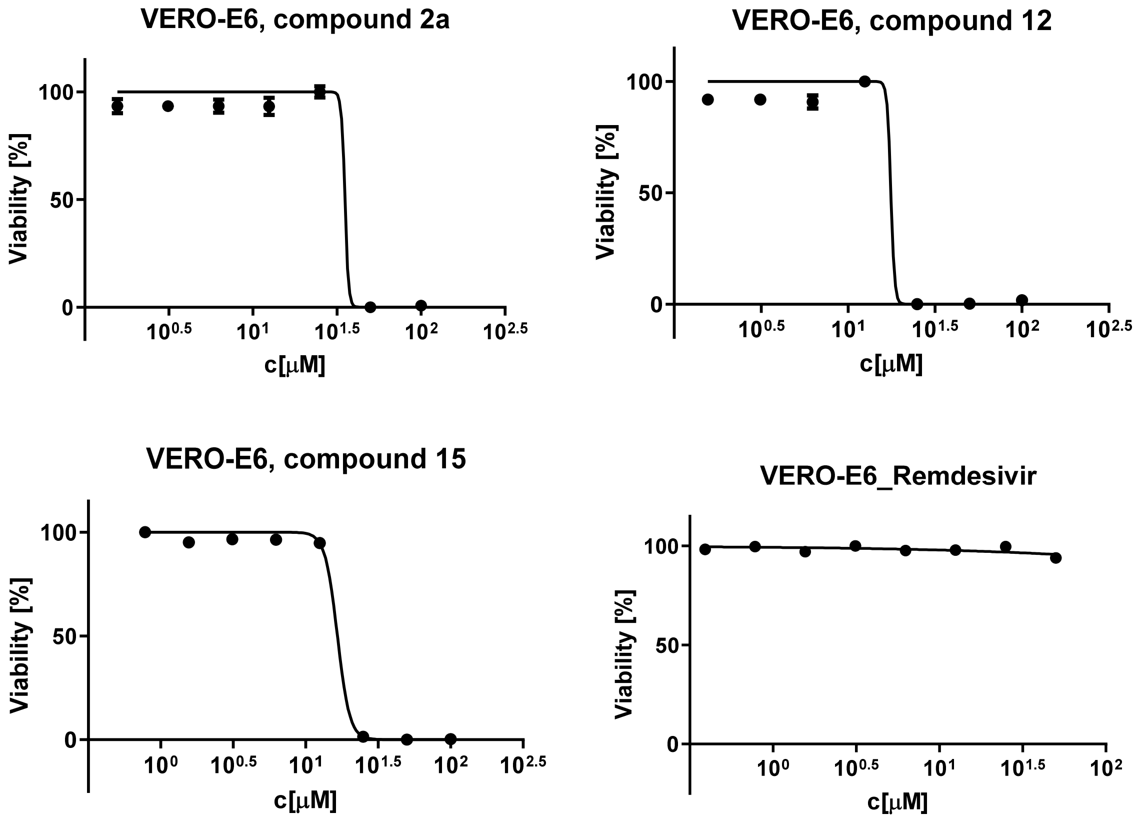

Figure S4. Cytotoxicity determination in Vero E6 cells

References

1. Lázár, L.; Csávás, M.; Herczeg, M.; Herczegh, P.; Borbás, A. Synthesis of S-Linked Glycoconjugates and S-Dissacharides by Thiol-Ene Coupling Reaction of Enoses, *Org. Lett.* **2012**, *14*, 4650–4653

2. F. Gauthier, A. Malher, J.-J. Vasseur, C. Dupouy, F. Debart, Conjugation of Small Molecules to RNA Using a Reducible Disulfide Linker Attached at the 2′-OH Position through a Carbamate Function, *Eur. J. Org. Chem.* **2019**, 5636–5645.

3. T.-S. Lin, M.-Z. Luo, M.-C. Liu, R. H. Clarke-Katzenburg, Y.-C. Cheng, W.H. Prusoff, W. R. Mancini, G. I. Birnbaum, E. J. Gabe, J. Giziewicz, Synthesis and Anticancer and Antiviral Activities of Various 2'- and 3'-Methylidene-Substituted Nucleoside Analogues and Crystal Structure of 2'-Deoxy-2'-methylidenecytidine Hydrochloride, *J. Med. Chem.* **1991**,.*34*, 2607-2615.

4. Y. Masaki , K. Yamamoto , T. Inde , K. Yoshida , A. Maruyama, T. Nagata, J. Tanihata, S. Takeda, M. Sekine, K. Seioa, Synthesis of 2′-O-(N-methylcarbamoylethyl) 5-methyl-2-thiouridine and its application to splice-switching oligonucleotides, *Bioorg. Med. Chem. Lett.* **2019**, *29*. 160–163.

5. S. Buchini, C. J. Leumann, New Nucleoside Analogues for the Recognition of Pyrimidine-Purine Inversion Sites, *Nucleosides, Nucleotides & Nucleic Acids*, **2003**, *22*, 1199–1201.

6. M. R. Bockman, A. S. Kalinda, R. Petrelli, T. De la Mora-Rey, D. Tiwari, F. Liu, S. Dawadi, M. Nandakumar, K. Y. Rhee, D. Schnappinger, B. C. Finzel, C. C. Aldrich, Targeting *Mycobacterium tuberculosis* Biotin Protein Ligase (*Mt*BPL) with Nucleoside-Based Bisubstrate Adenylation Inhibitors, *J. Med. Chem.* **2015**, *58*, 7349–7369.

7. M. Zhong, S. A. Strobel, Synthesis of isotopically labeled P-site substrates for the ribosomal peptidyl transferase reaction, *J. Org. Chem.* **2008**, *73*, 603–611.

8. E.J. Reist, A. Benitez, L. Goodman, The Synthesis of Some S'-Thiopentofuranosylpyrimidines, *J. Chem. Soc*. **1964**, *29*, 554-558.

NMR spectra of the compounds

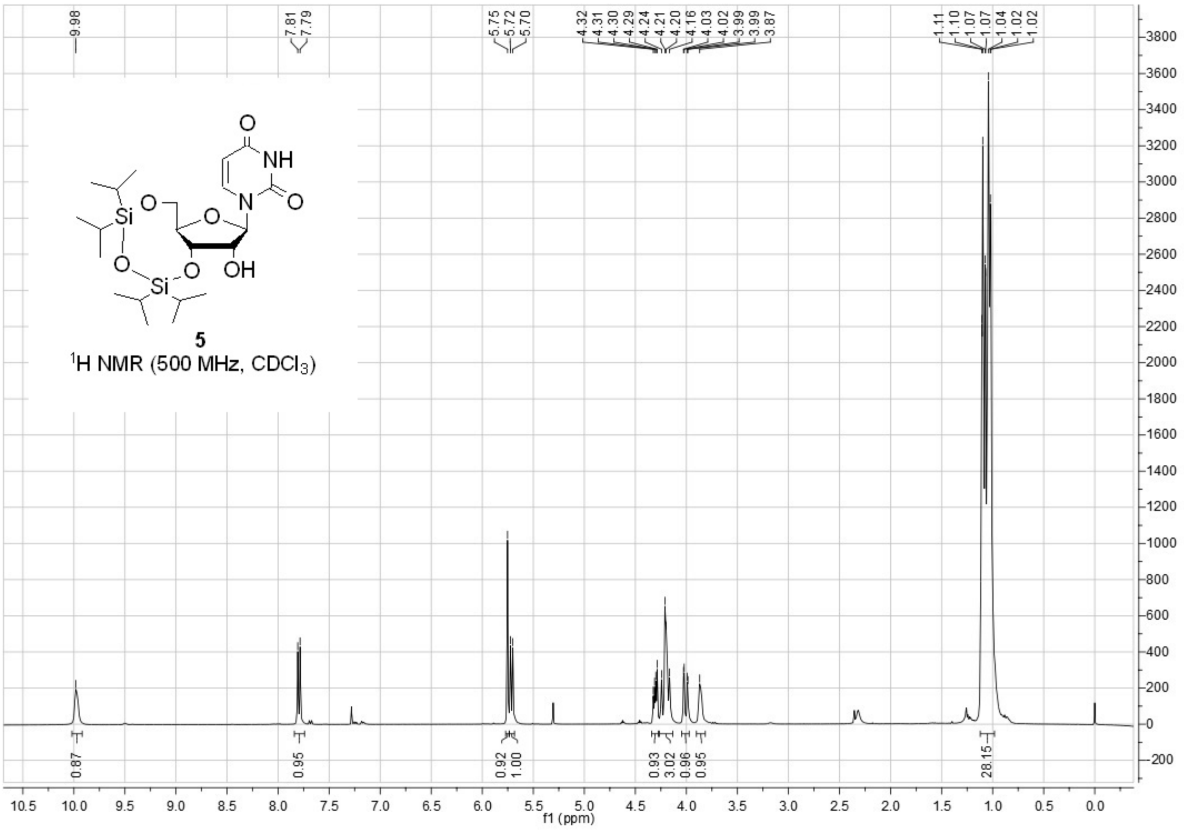

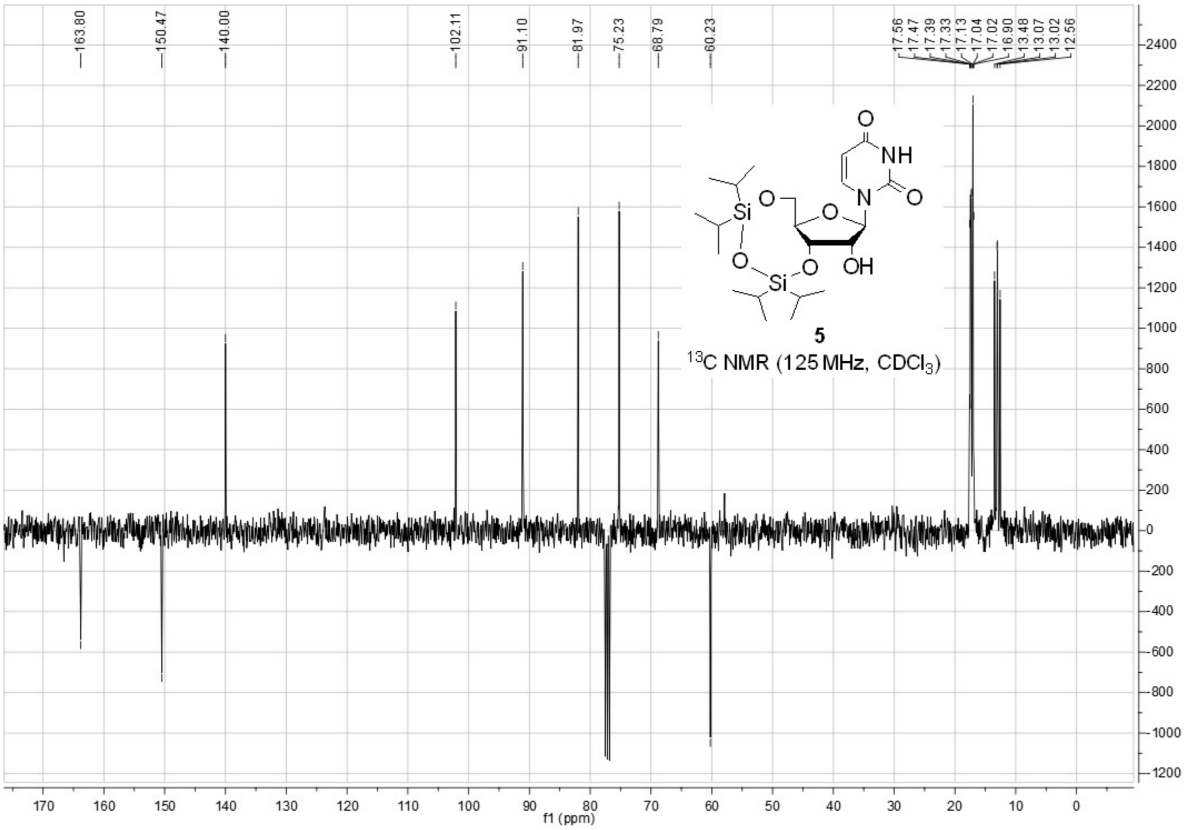

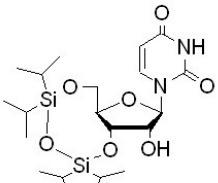

5

^{1}H-^{1}H COSY (500 MHz, $CDCl_3$)

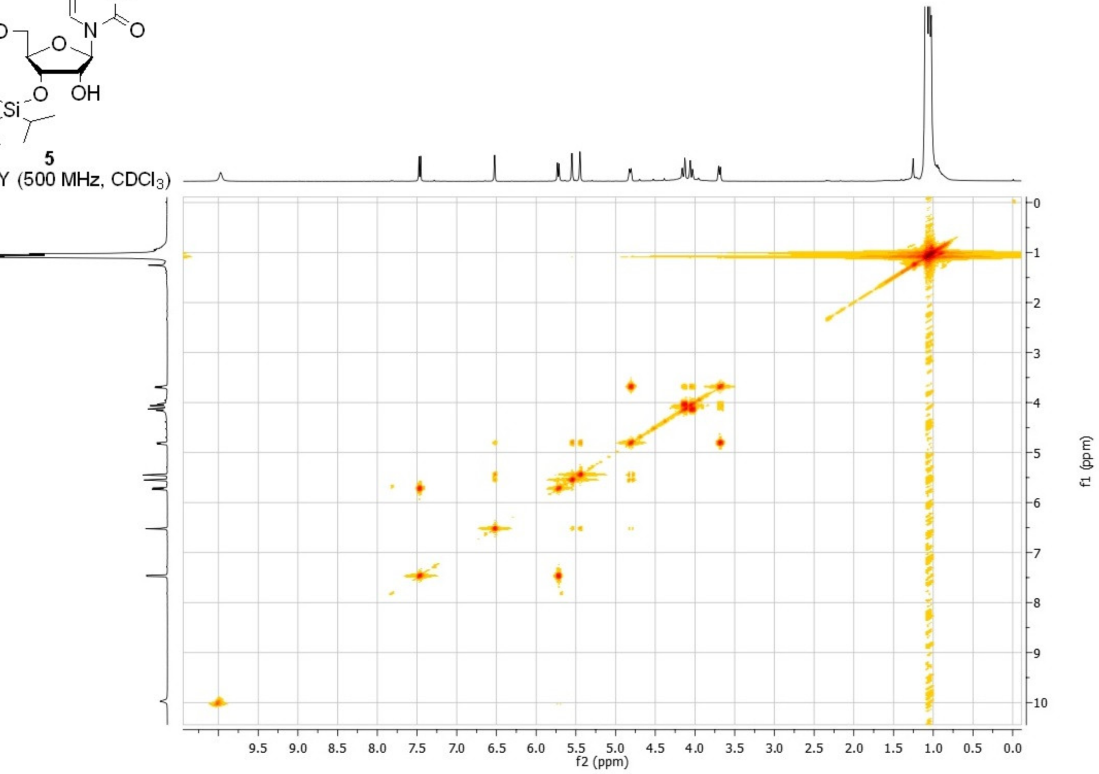

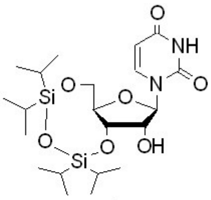

5

^{1}H-^{13}C HSQC (125 MHz, $CDCl_3$)

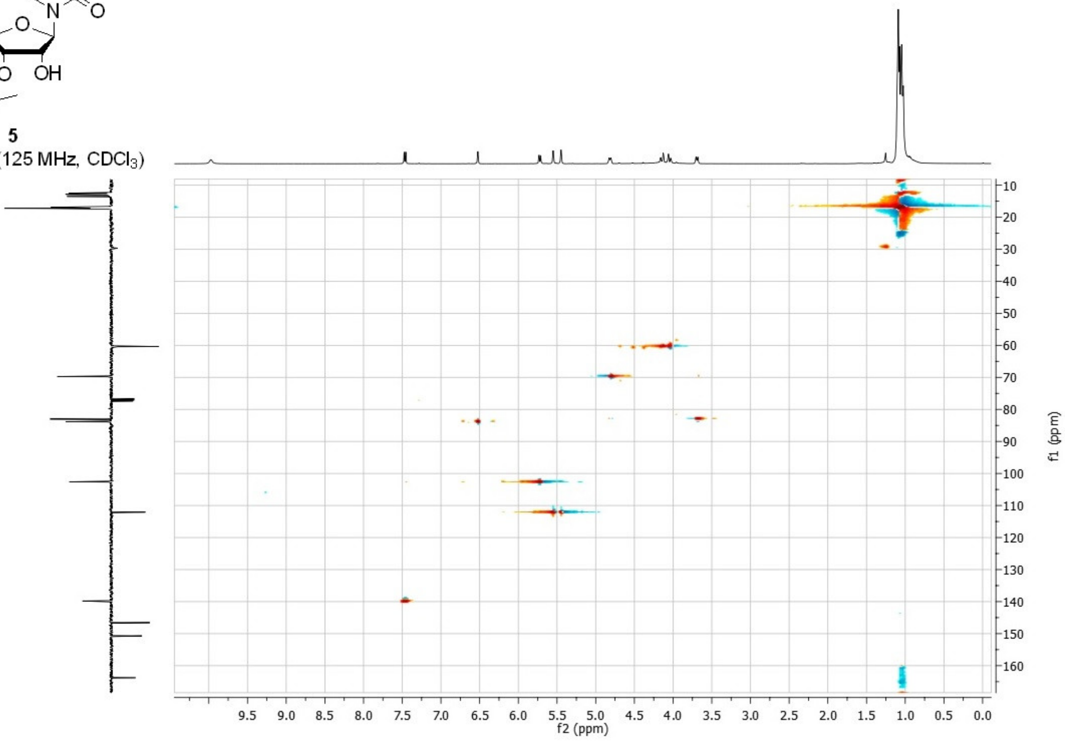

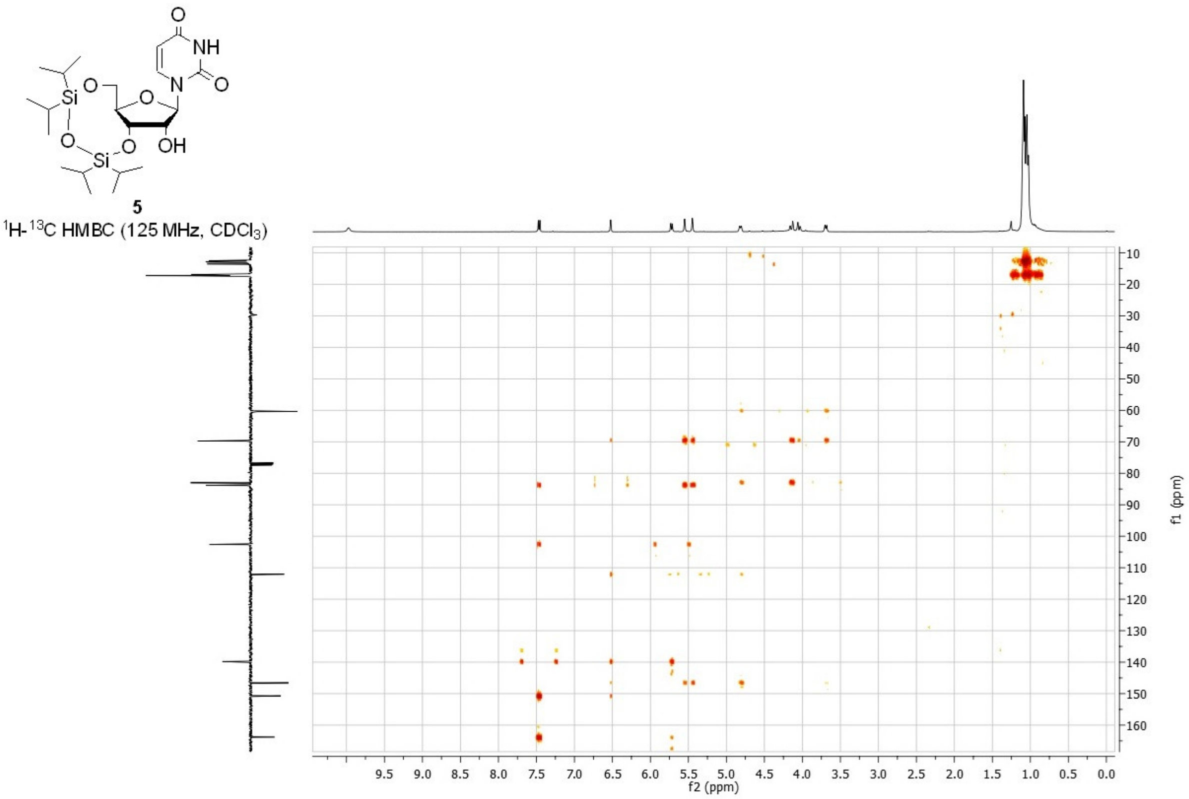

5

^{1}H-^{13}C HMBC (125 MHz, $CDCl_3$)

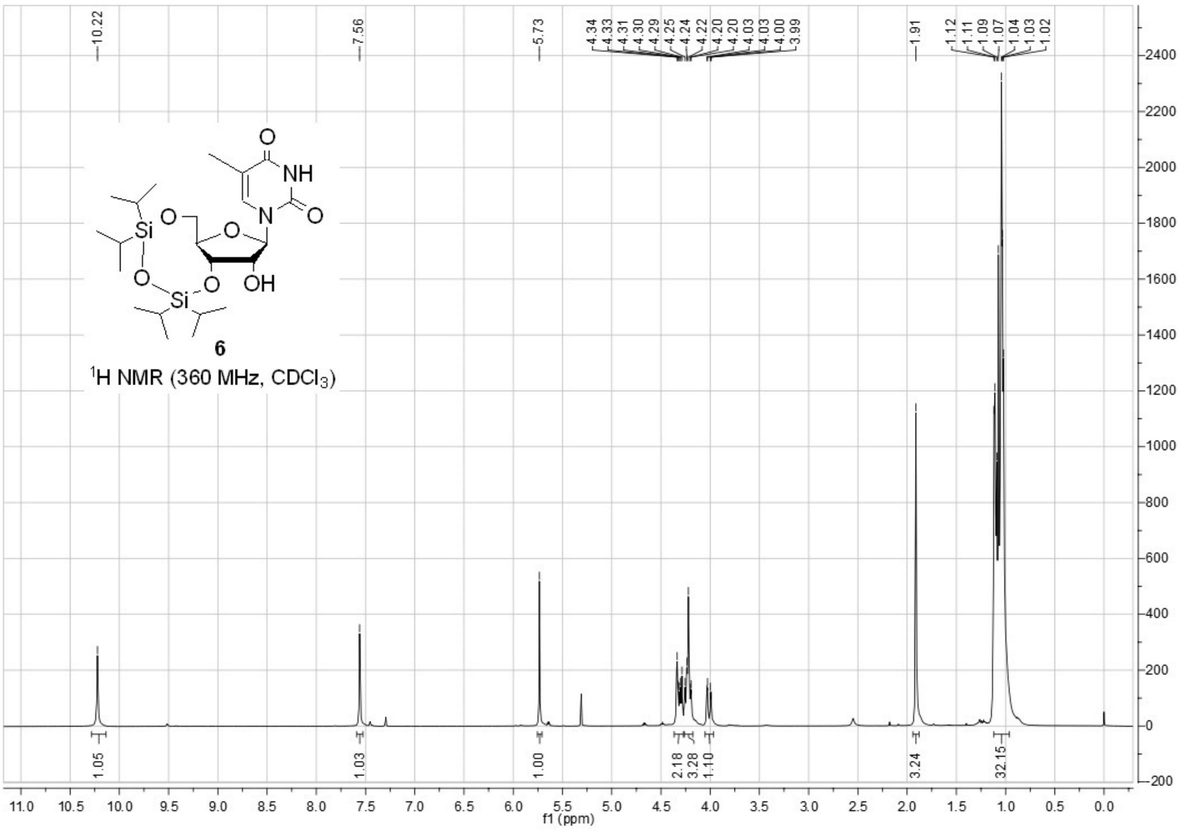

6

^{1}H NMR (360 MHz, $CDCl_3$)

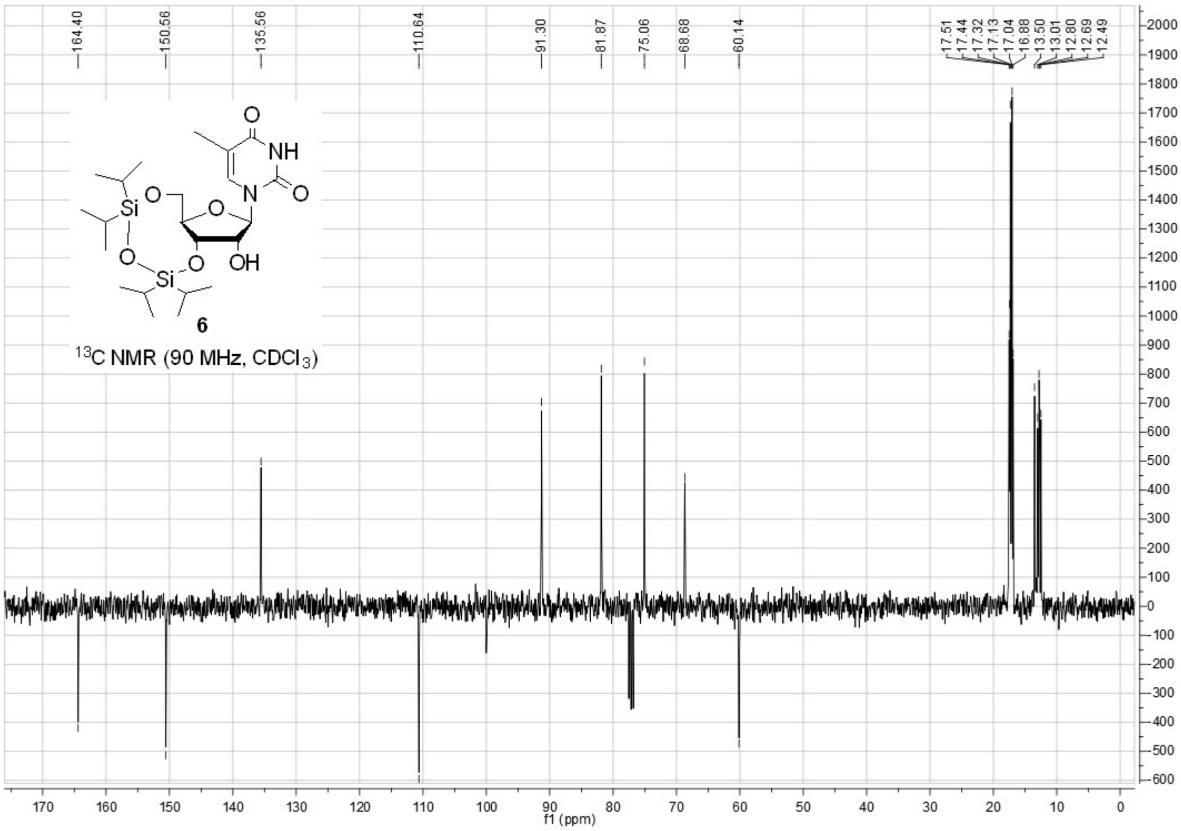
164.40
150.56
135.56
110.64
91.30
81.87
75.06
68.68
60.14
17.51
17.44
17.32
17.13
17.04
16.88
13.50
13.01
12.80
12.69
12.49
6
^{13}C NMR (90 MHz, $CDCl_3$)
f1 (ppm)

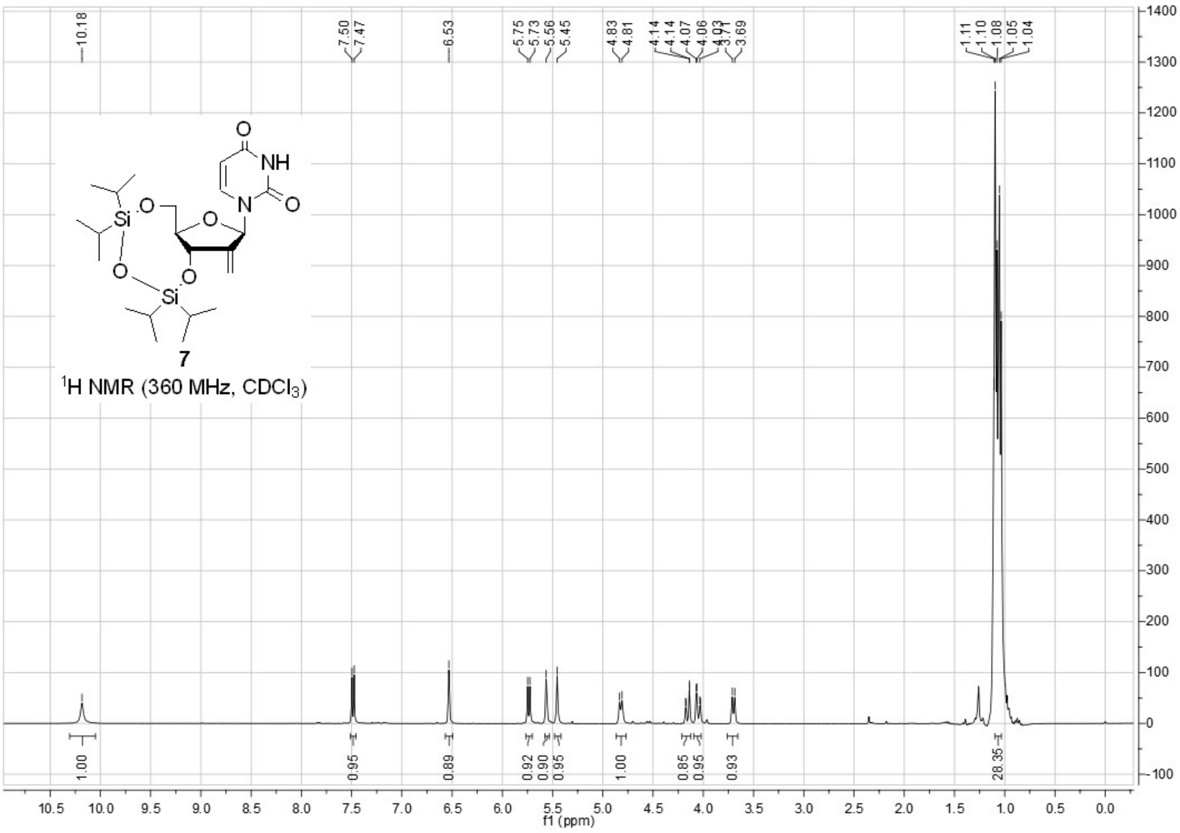
10.18
7.50
7.47
6.53
5.75
5.73
5.56
5.45
4.83
4.81
4.14
4.14
4.07
4.06
4.03
3.71
3.69
1.11
1.10
1.08
1.05
1.04
7
^1H NMR (360 MHz, $CDCl_3$)
1.00
0.95
0.89
0.92
0.90
0.95
1.00
0.85
0.95
0.93
28.35
f1 (ppm)

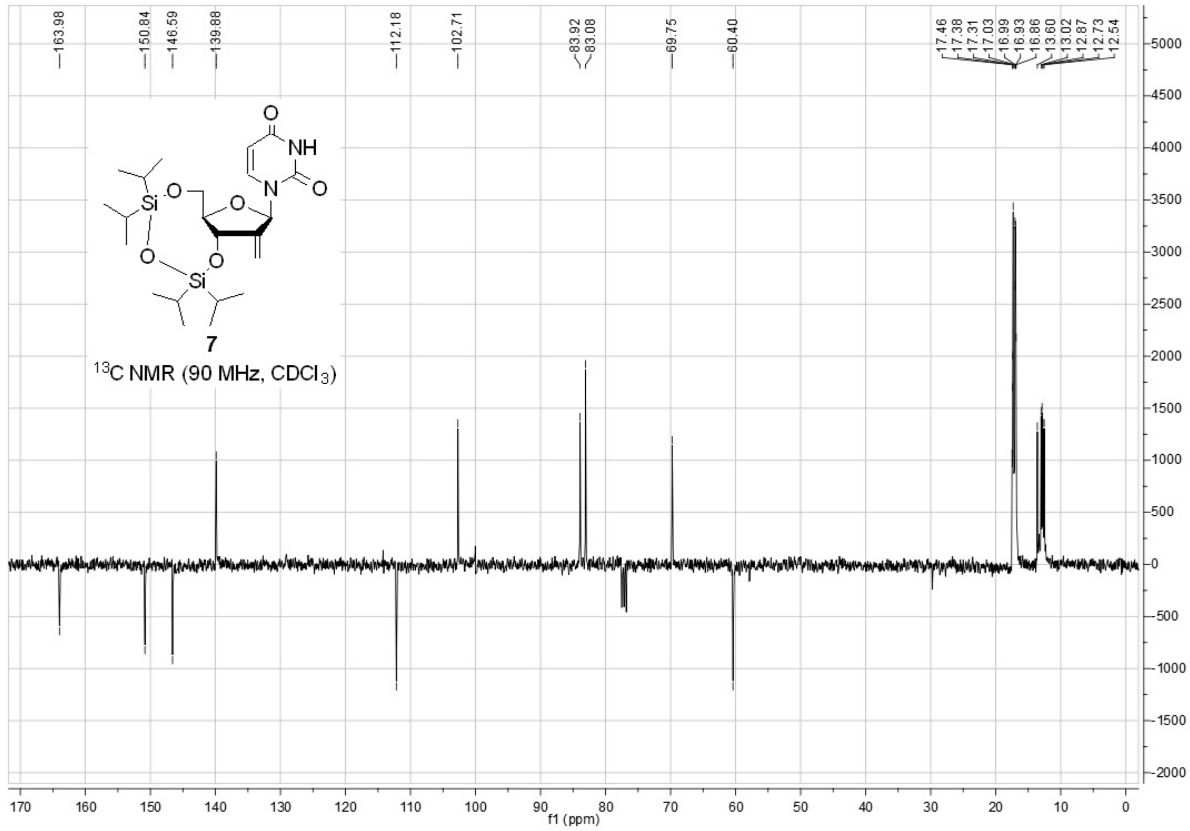
13C NMR (90 MHz, CDCl3)
7
f1 (ppm)

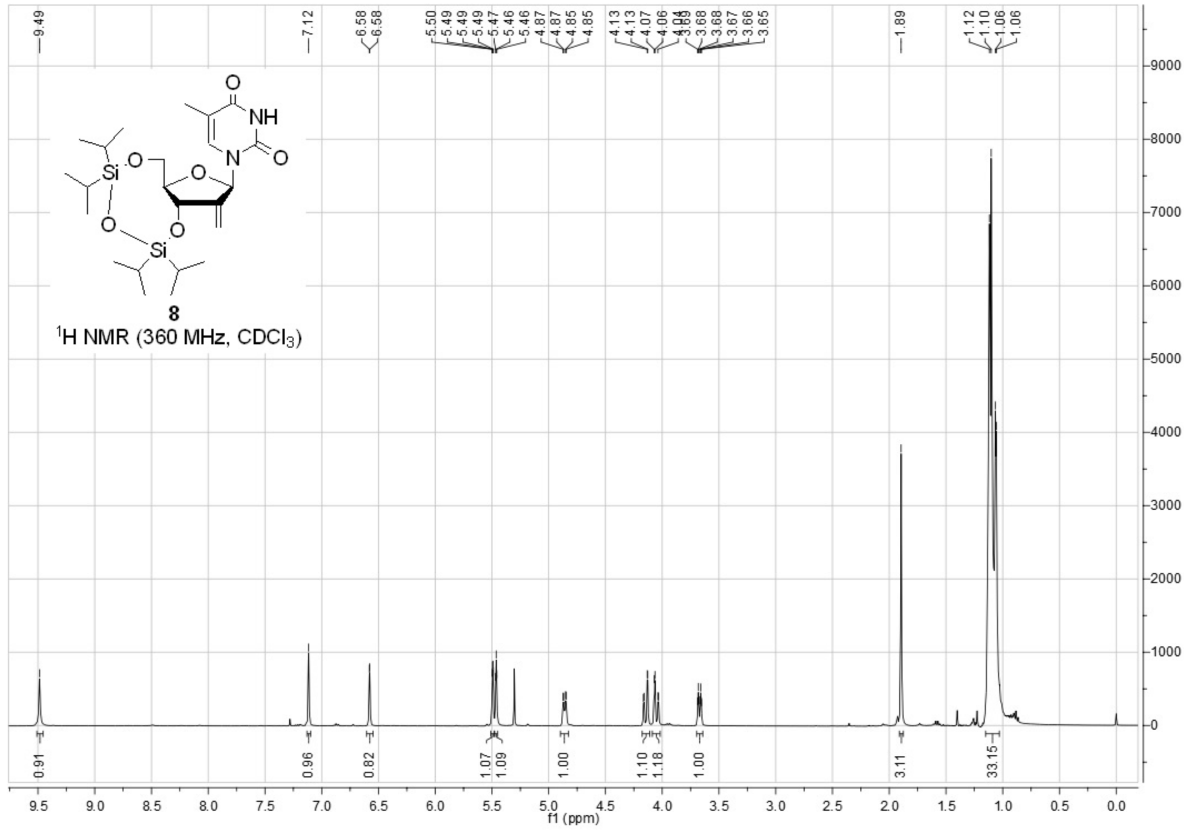
1H NMR (360 MHz, CDCl3)
8
f1 (ppm)

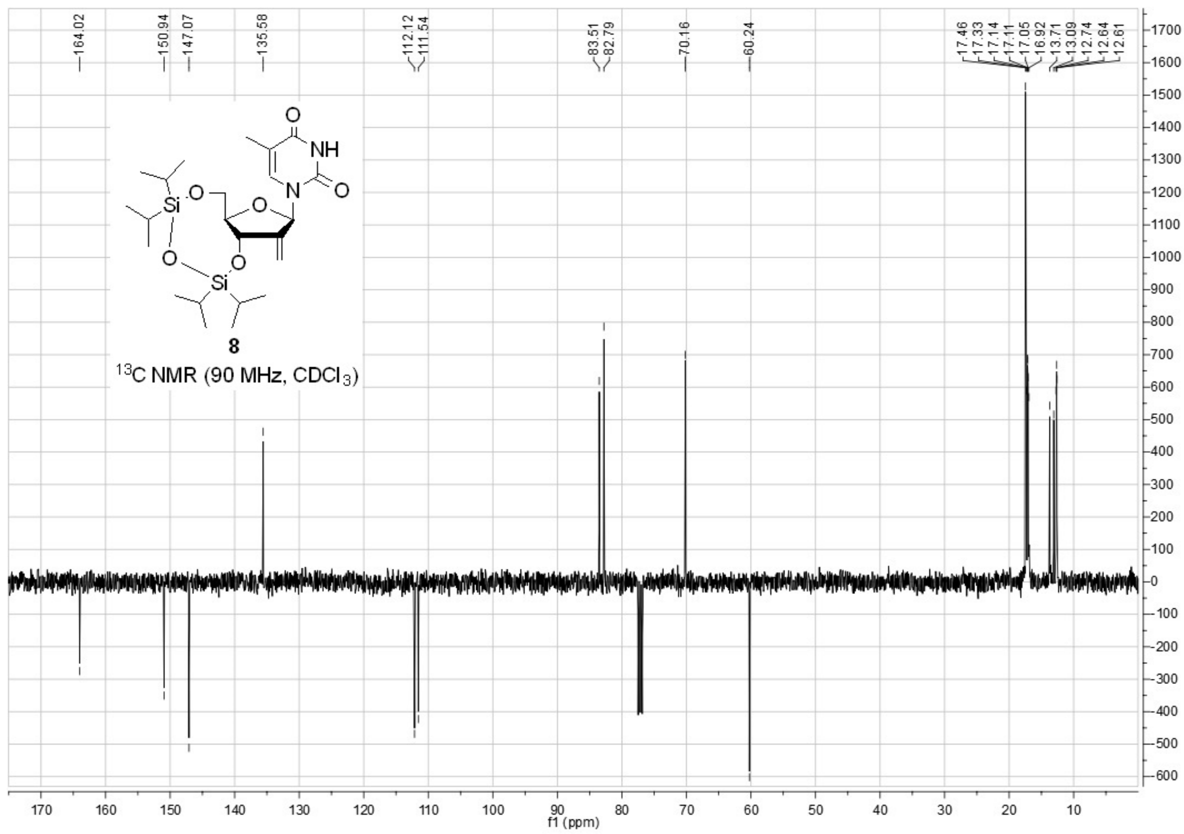

8
^{13}C NMR (90 MHz, $CDCl_3$)
f1 (ppm)

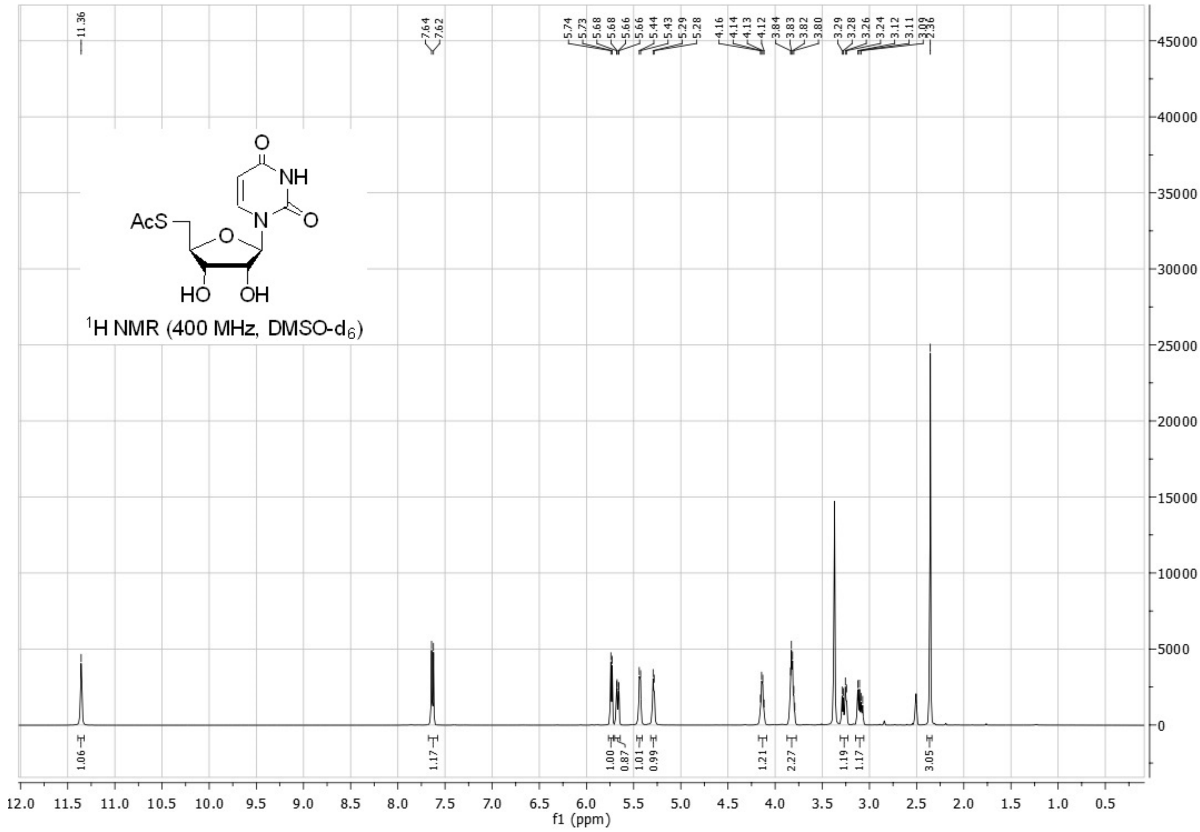

AcS
HO
OH
^{1}H NMR (400 MHz, DMSO-d_6)
f1 (ppm)

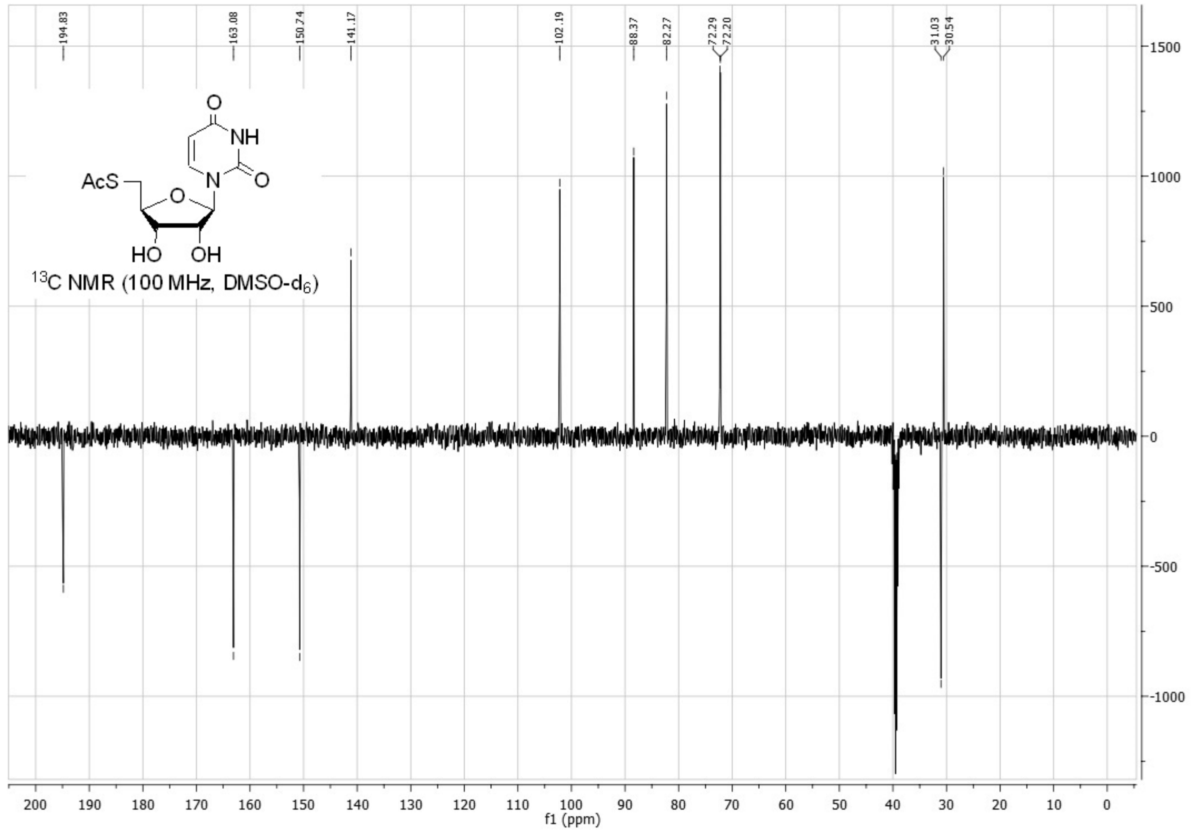
194.83
163.08
150.74
141.17
102.19
88.37
82.27
72.29
72.20
31.03
30.54
AcS
O
NH
N
HO
OH
13C NMR (100 MHz, DMSO-d6)
f1 (ppm)

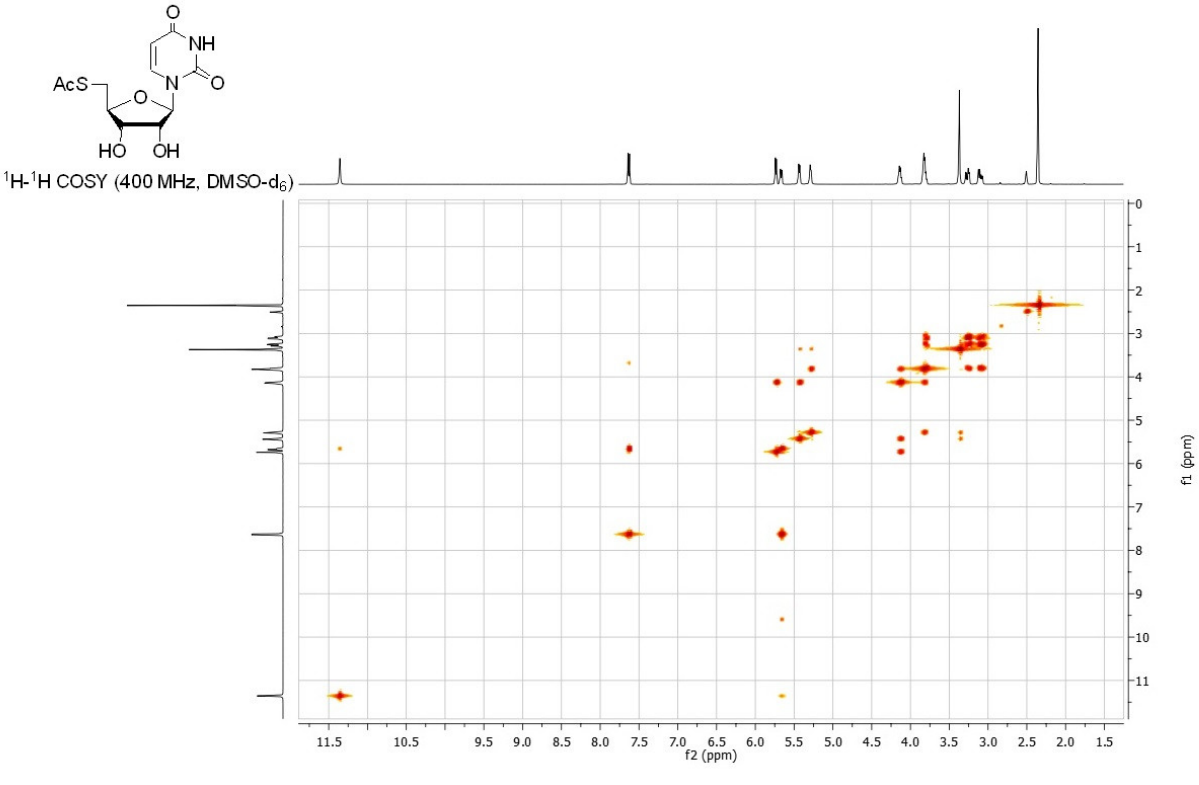
AcS
O
NH
N
HO
OH
1H-1H COSY (400 MHz, DMSO-d6)
f2 (ppm)
f1 (ppm)

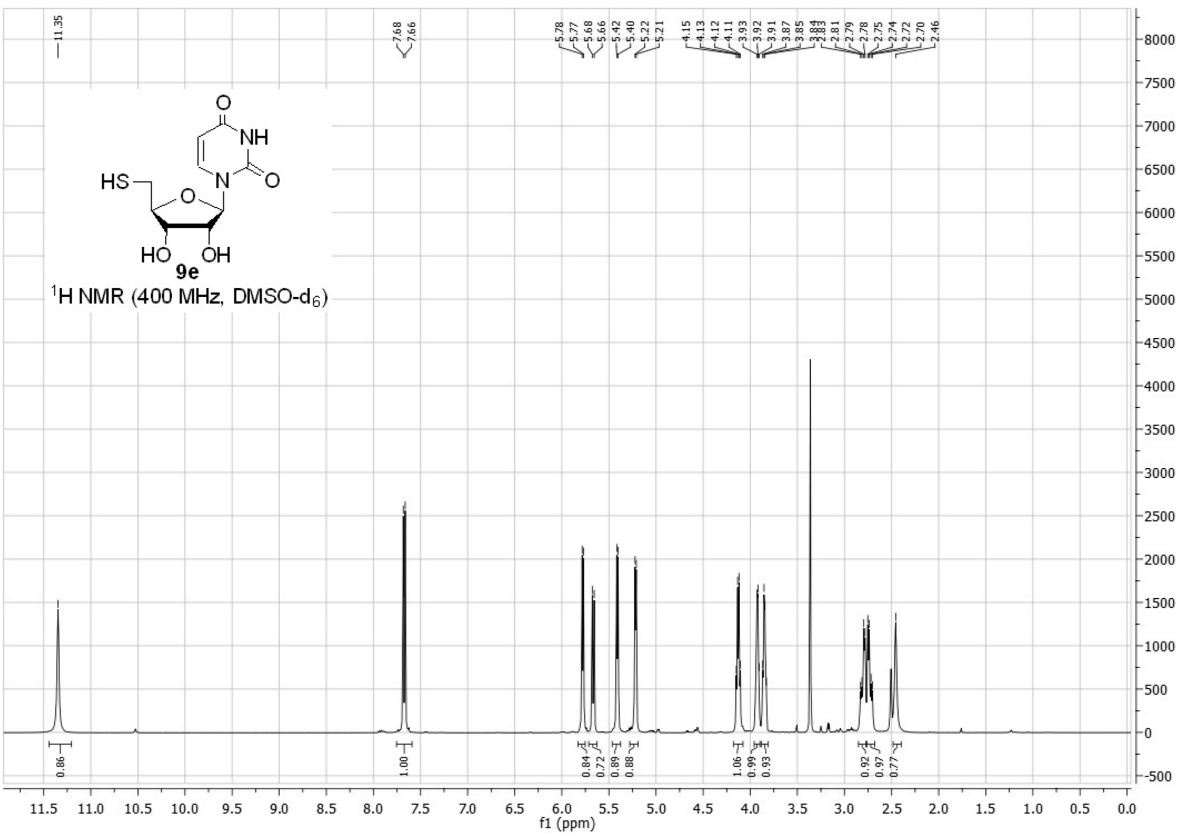
HS
O
NH
N
O
HO
OH
9e
1H NMR (400 MHz, DMSO-d6)
f1 (ppm)

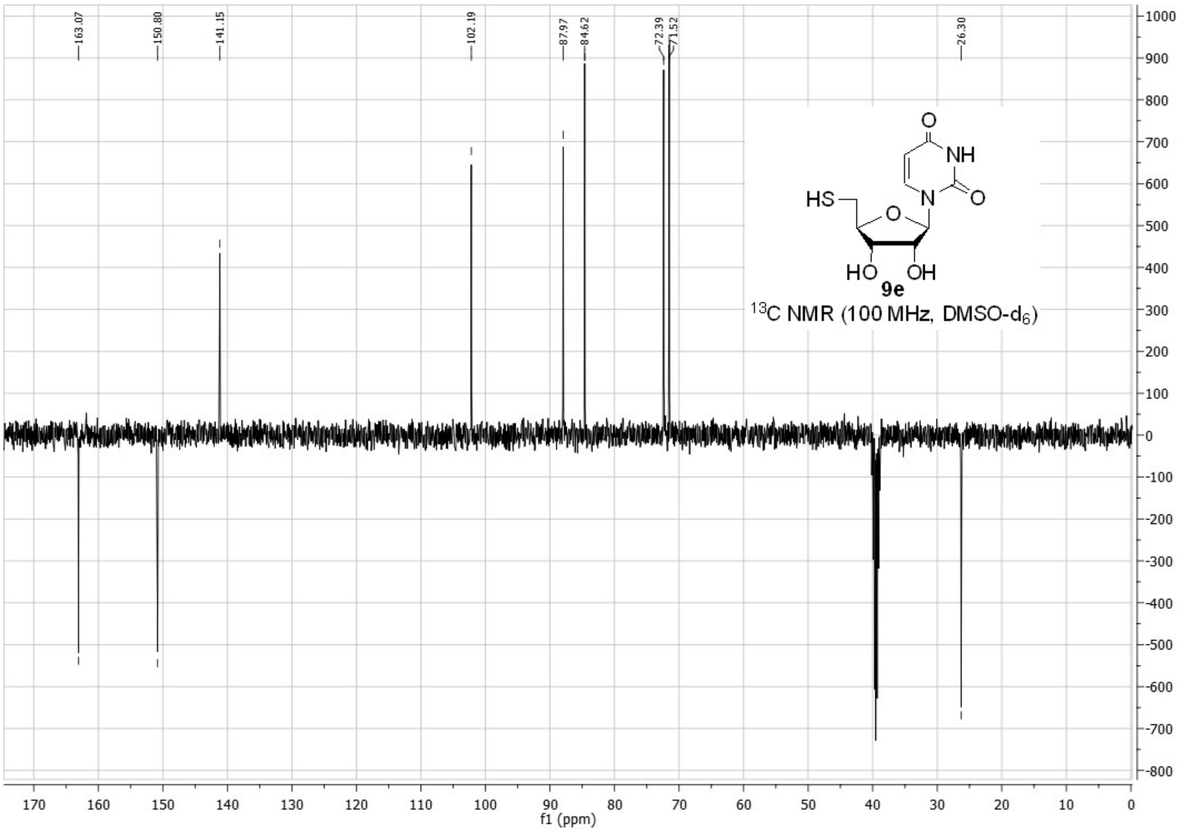
HS
O
NH
N
O
HO
OH
9e
13C NMR (100 MHz, DMSO-d6)
f1 (ppm)

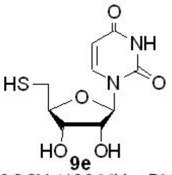

^{1}H-^{1}H COSY (400 MHz, DMSO-d_6)

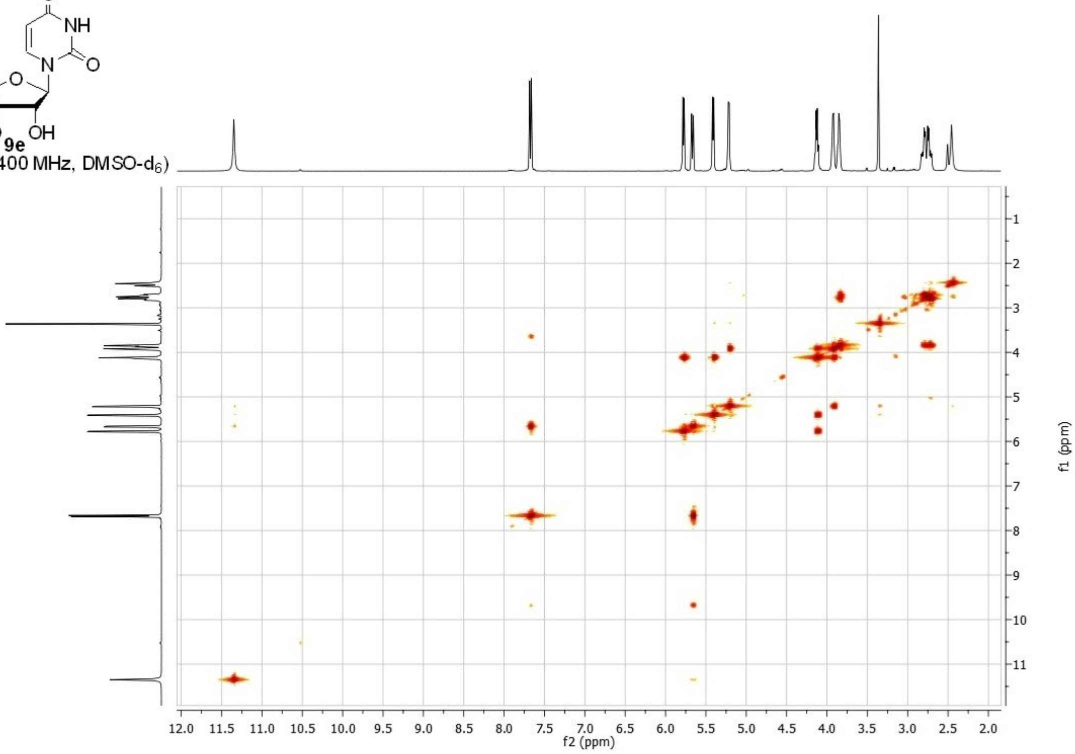

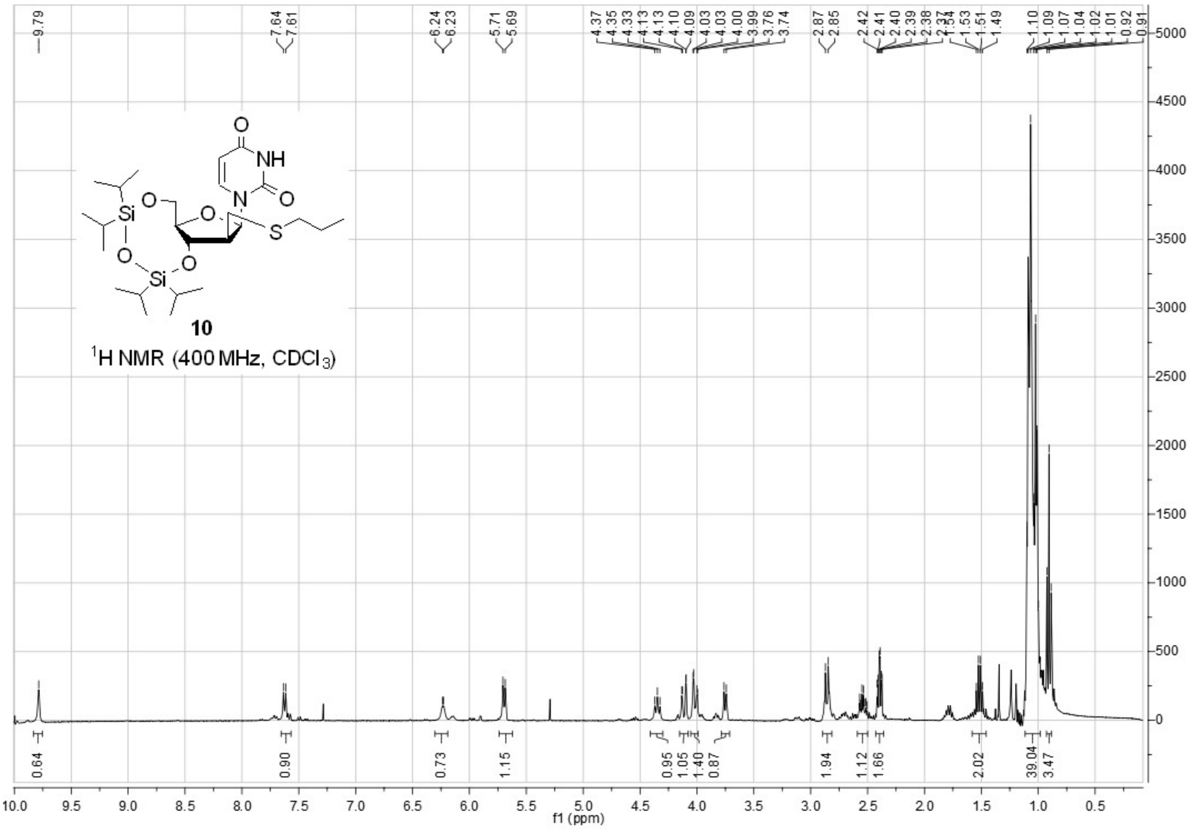
10
^{1}H NMR (400 MHz, CDCl$_3$)

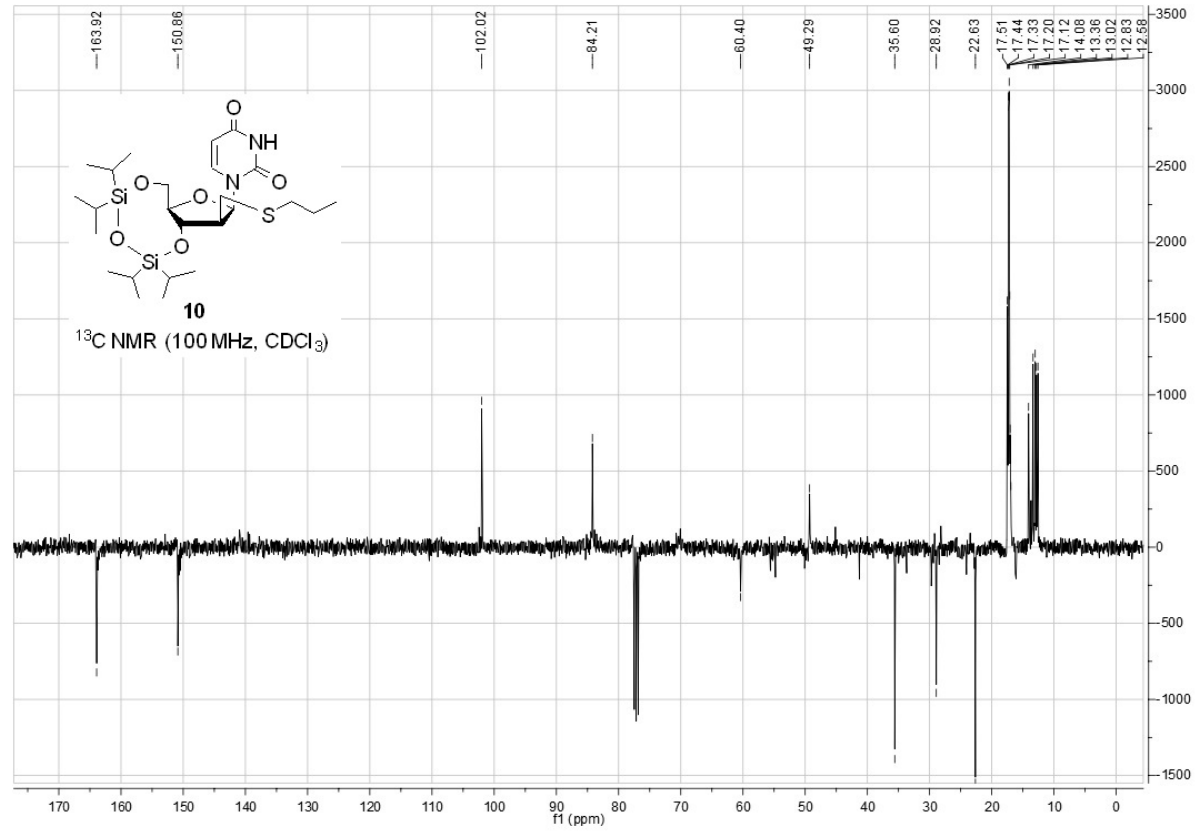
10
^{13}C NMR (100 MHz, CDCl$_3$)

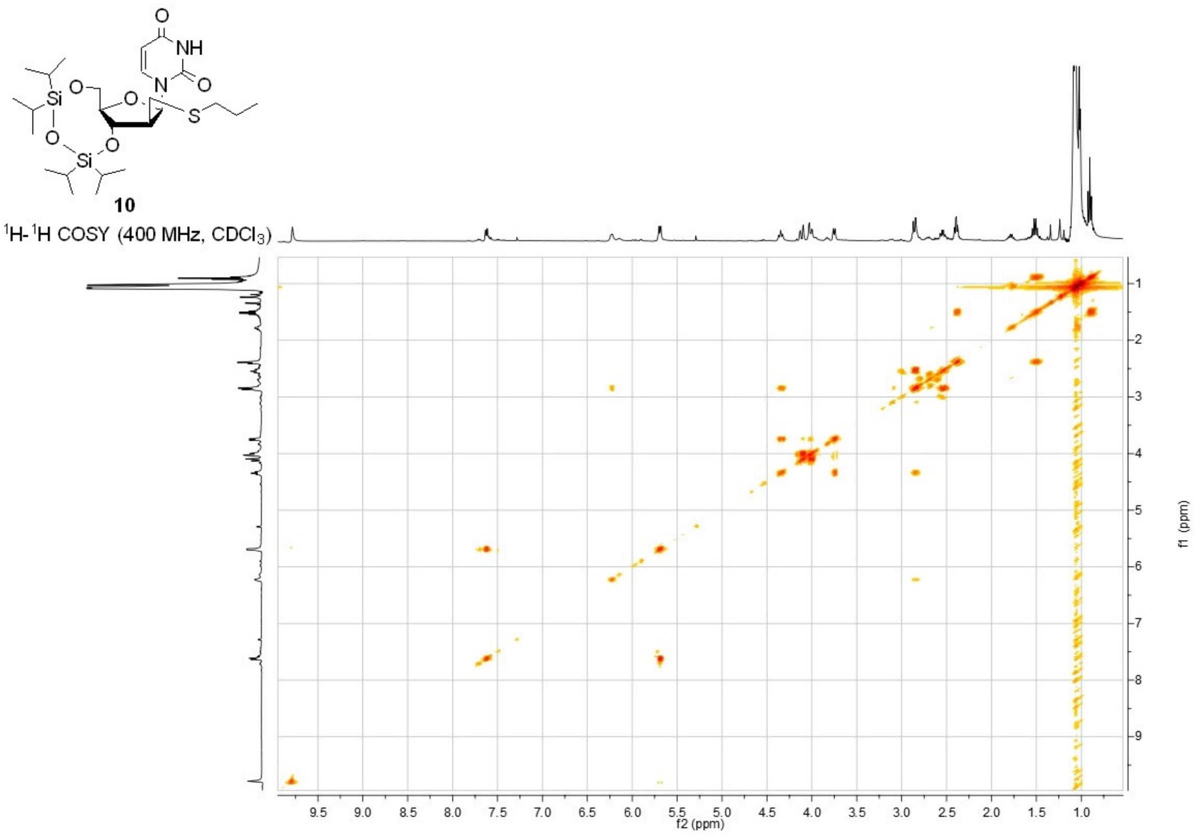

10

^{1}H-^{1}H COSY (400 MHz, CDCl$_3$)

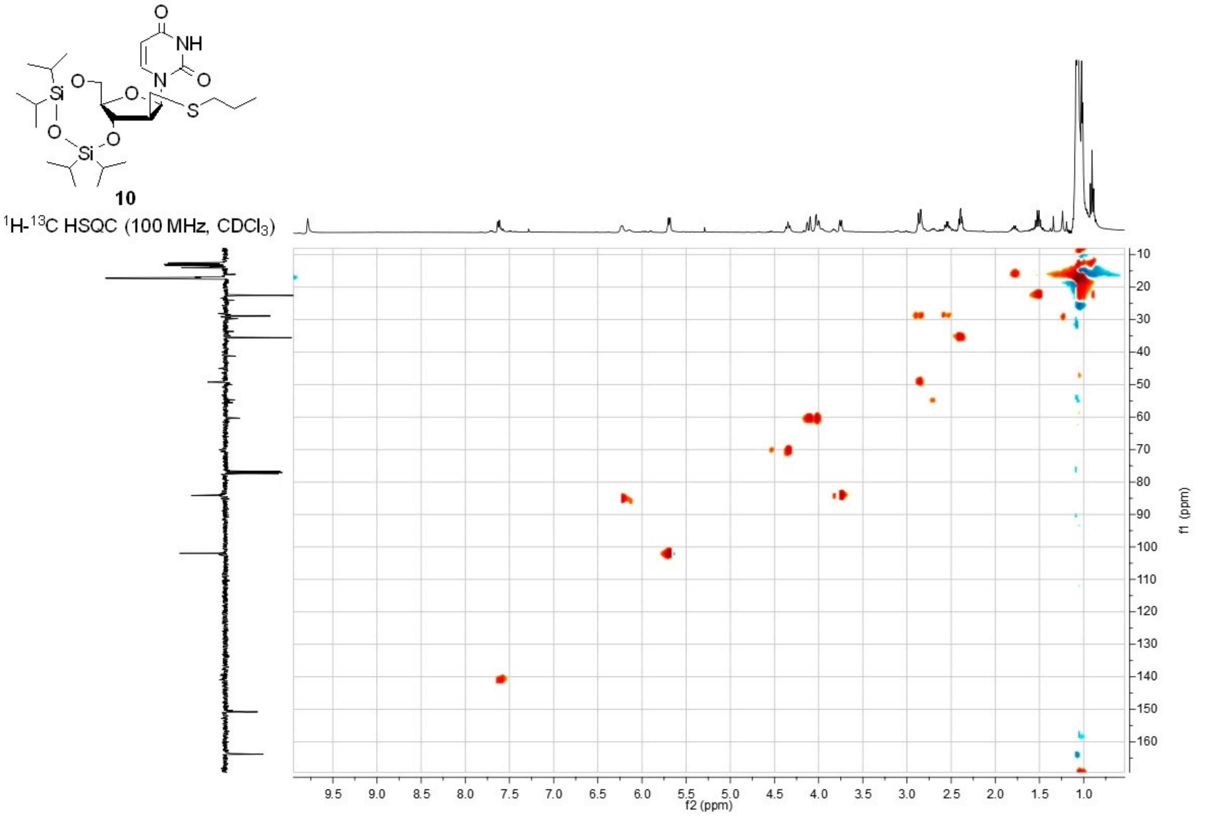

10

^{1}H-^{13}C HSQC (100 MHz, CDCl$_3$)

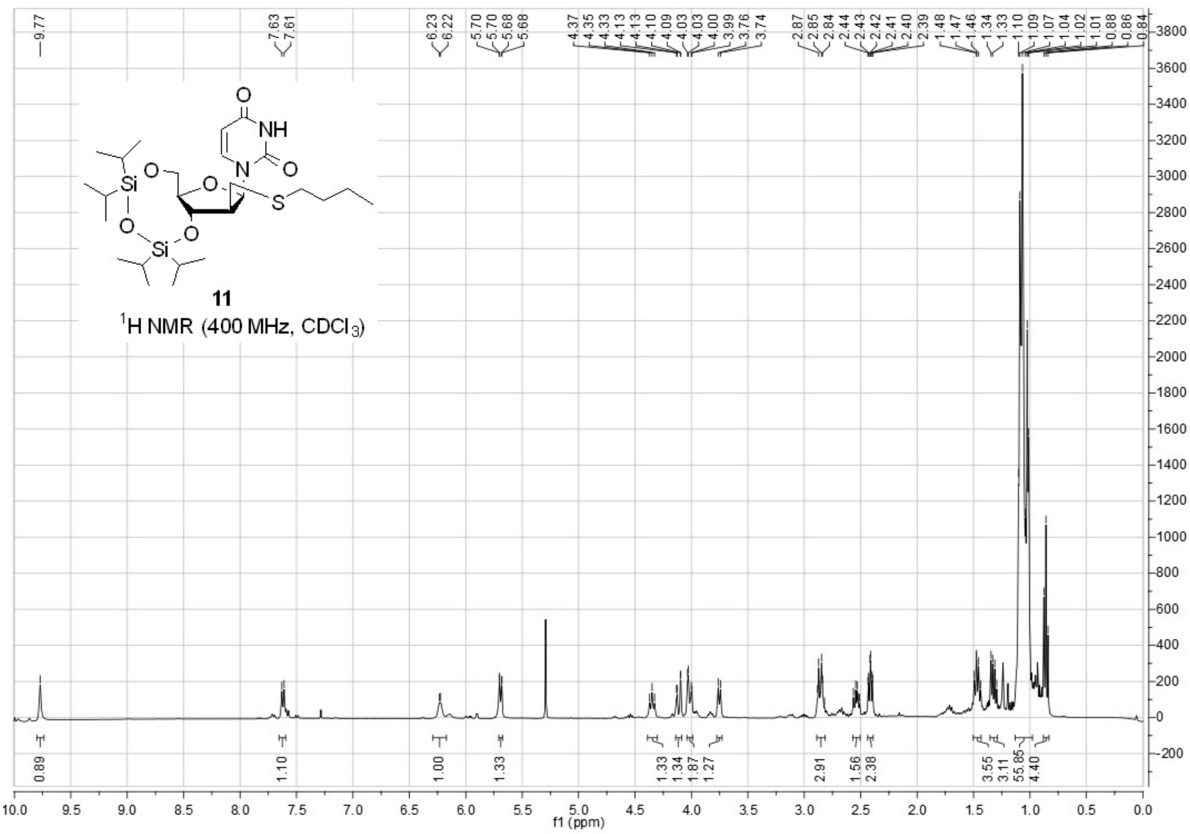
9.77
7.63
7.61
6.23
6.22
5.70
5.70
5.68
5.68
4.37
4.35
4.33
4.13
4.13
4.10
4.09
4.03
4.03
4.00
3.99
3.76
3.74
2.87
2.85
2.84
2.44
2.43
2.42
2.41
2.40
2.39
1.48
1.47
1.46
1.34
1.33
1.10
1.09
1.07
1.04
1.02
1.01
0.88
0.86
0.84
O
NH
O
N
O
Si
O
S
O
O
Si
O
11
1H NMR (400 MHz, CDCl3)
0.89
1.10
1.00
1.33
1.33
1.34
1.87
1.27
2.91
1.56
2.38
3.55
3.11
55.85
4.40
f1 (ppm)

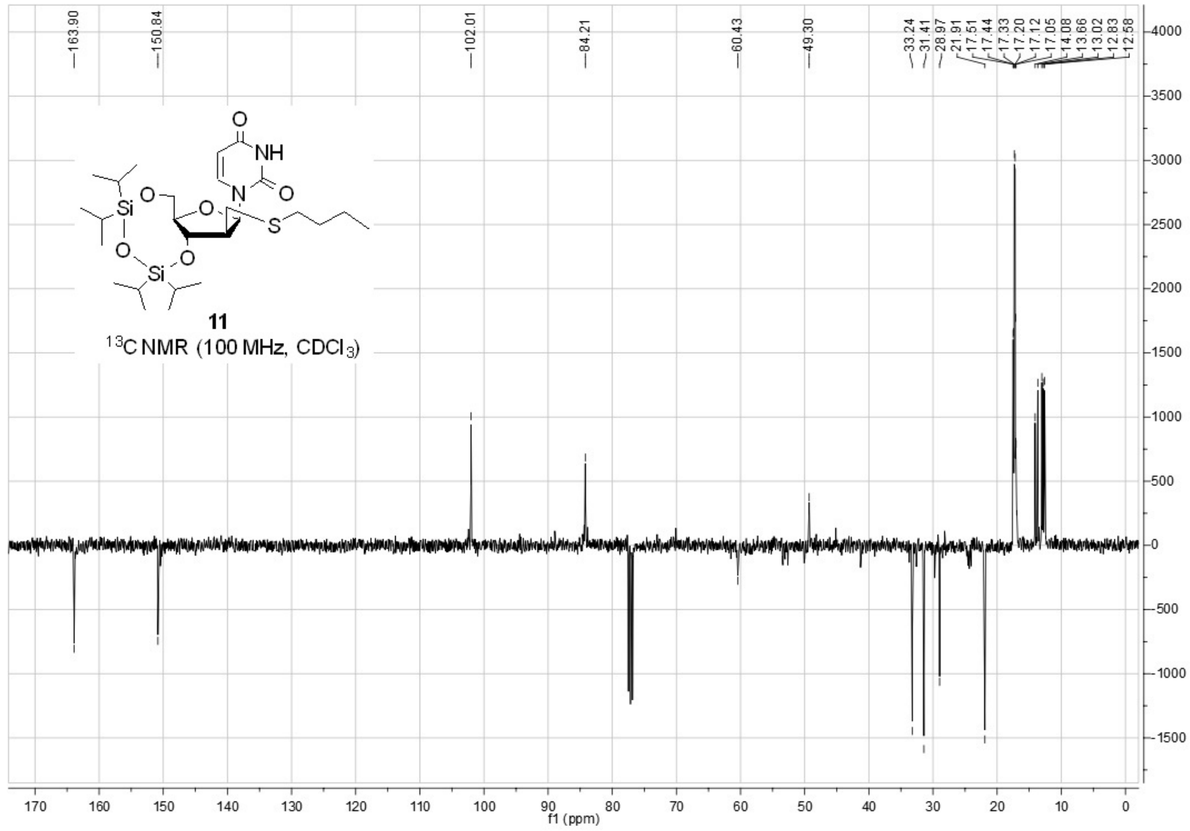
163.90
150.84
102.01
84.21
60.43
49.30
33.24
31.41
28.97
21.91
17.51
17.44
17.33
17.20
17.12
17.05
14.08
13.66
13.02
12.83
12.58
O
NH
O
N
O
Si
O
S
O
O
Si
O
11
13C NMR (100 MHz, CDCl3)
f1 (ppm)

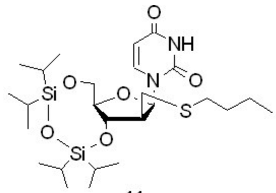

11

^{1}H-^{1}H COSY (400 MHz, $CDCl_3$)

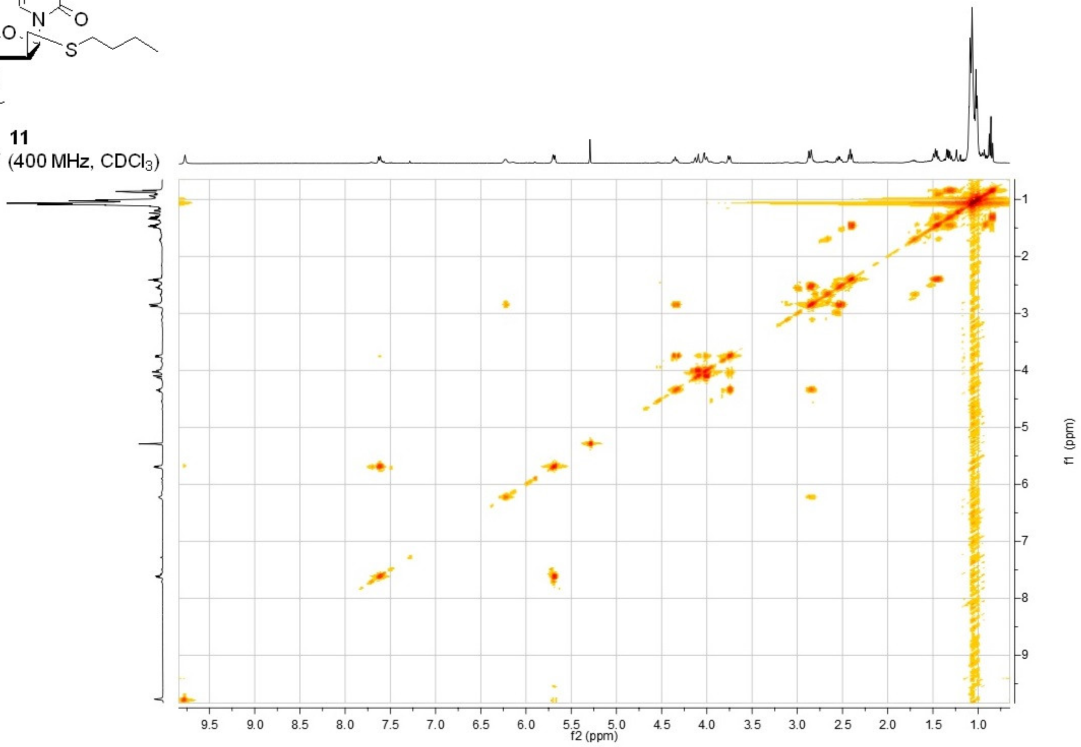

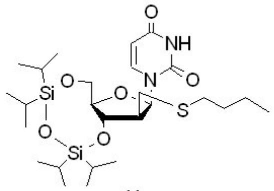

11

^{1}H-^{13}C HSQC (100 MHz, $CDCl_3$)

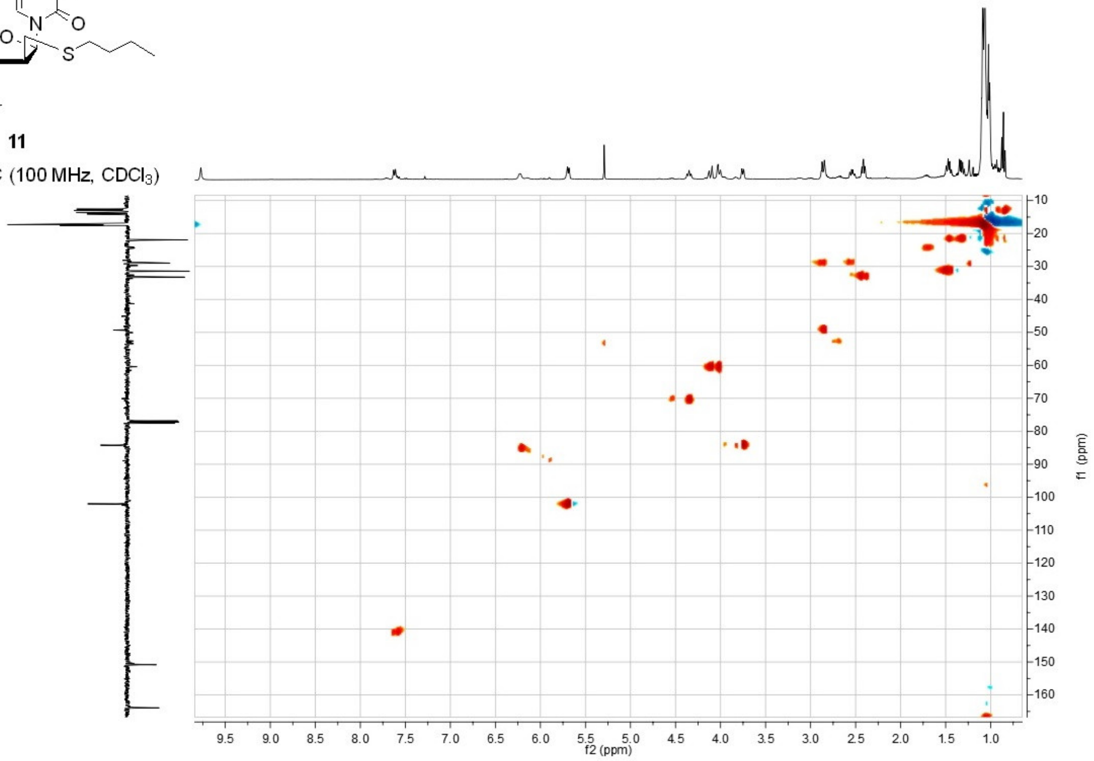

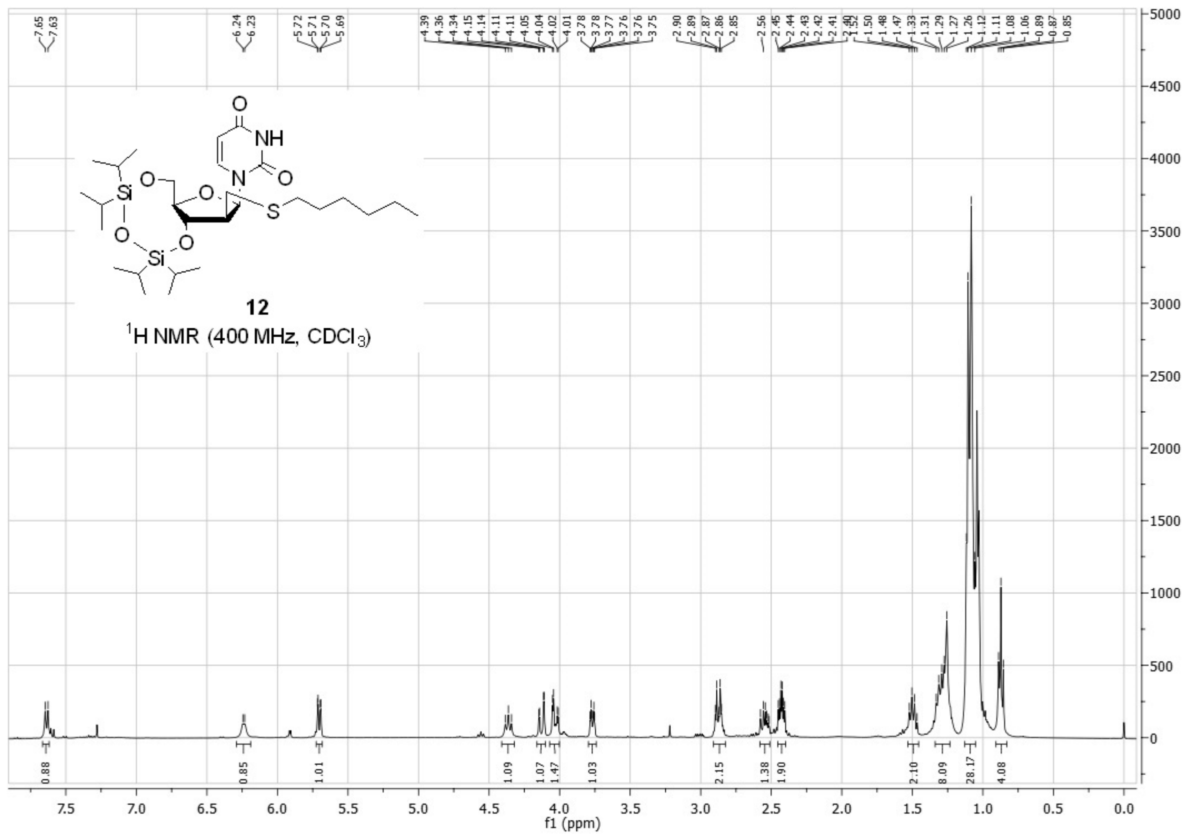
12
^{1}H NMR (400 MHz, CDCl$_3$)
f1 (ppm)

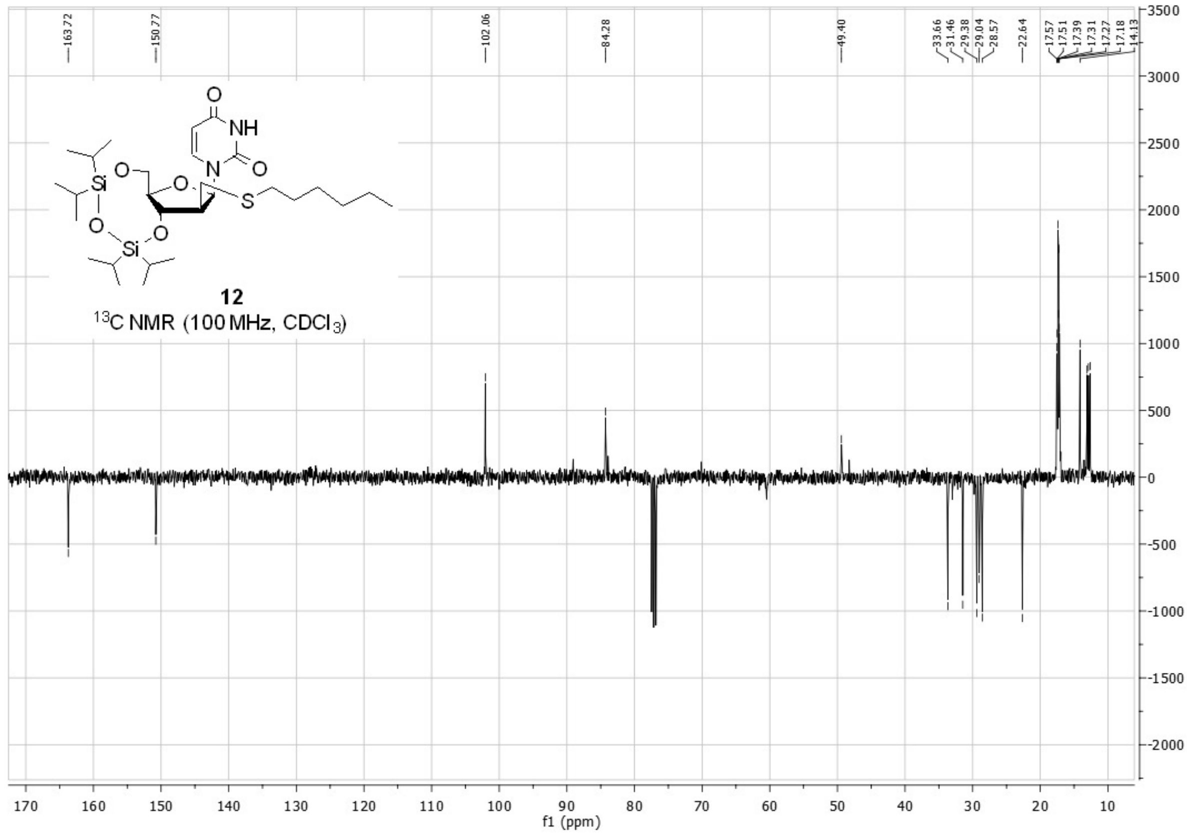
12
^{13}C NMR (100 MHz, CDCl$_3$)
f1 (ppm)

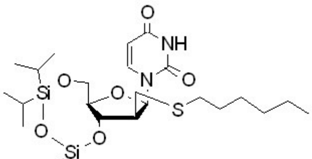

12

^{1}H-^{1}H COSY (400 MHz, $CDCl_3$)

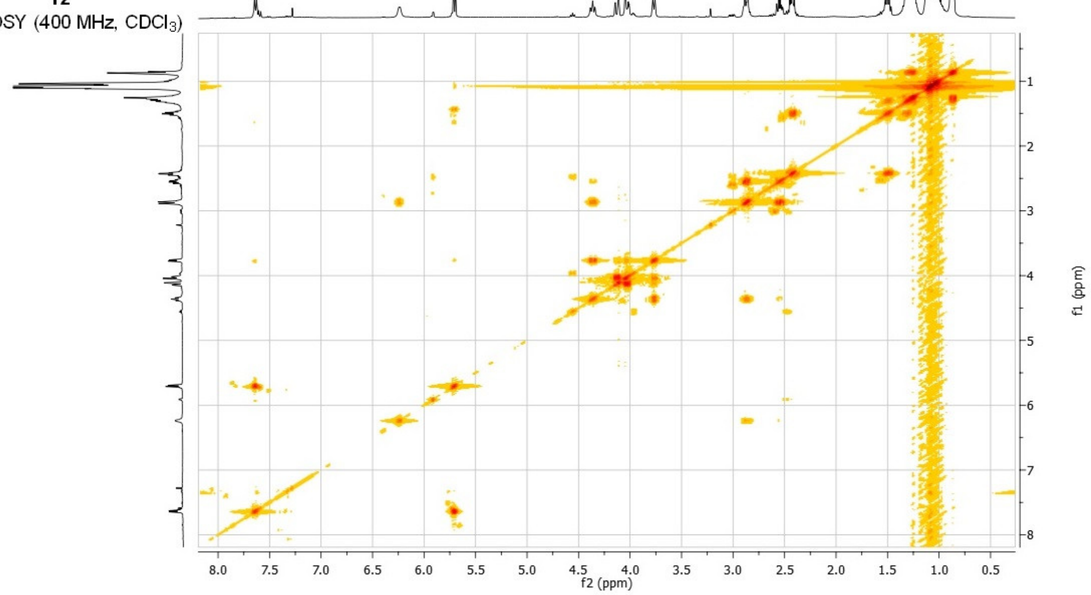

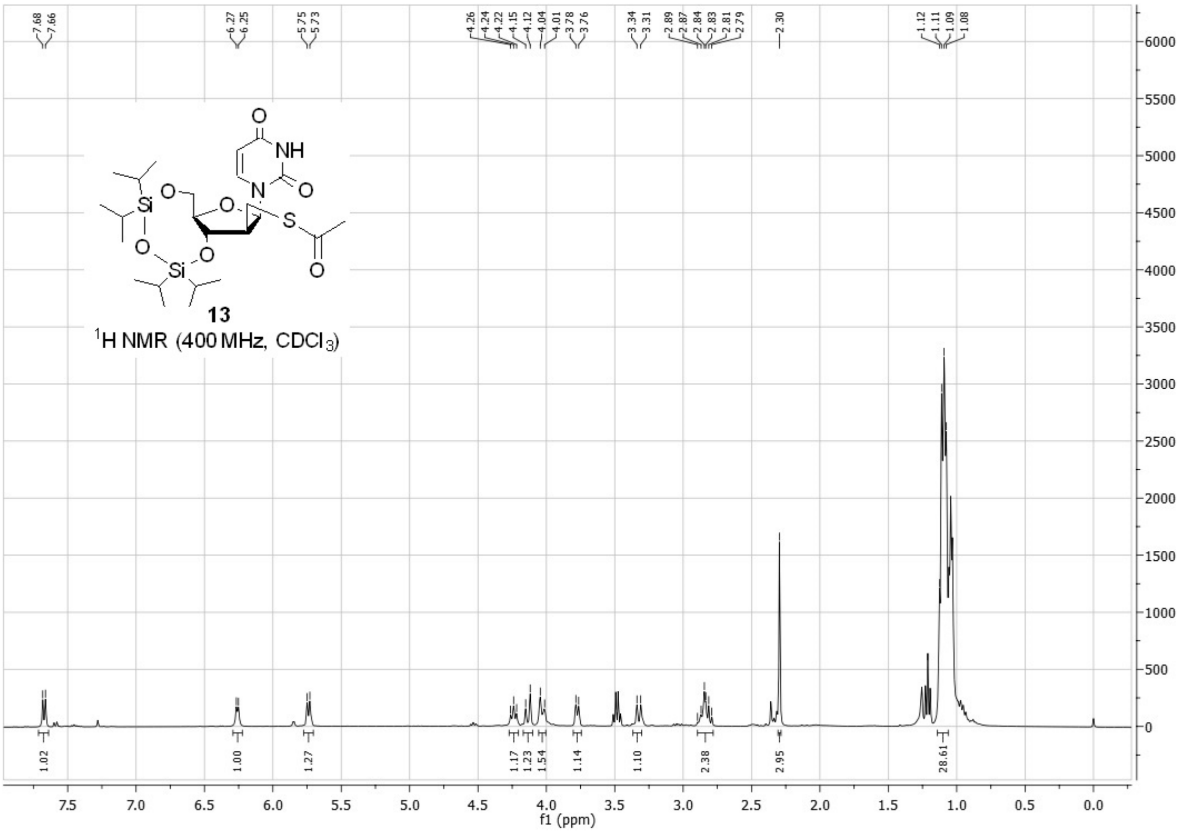

13

^{1}H NMR (400 MHz, $CDCl_3$)

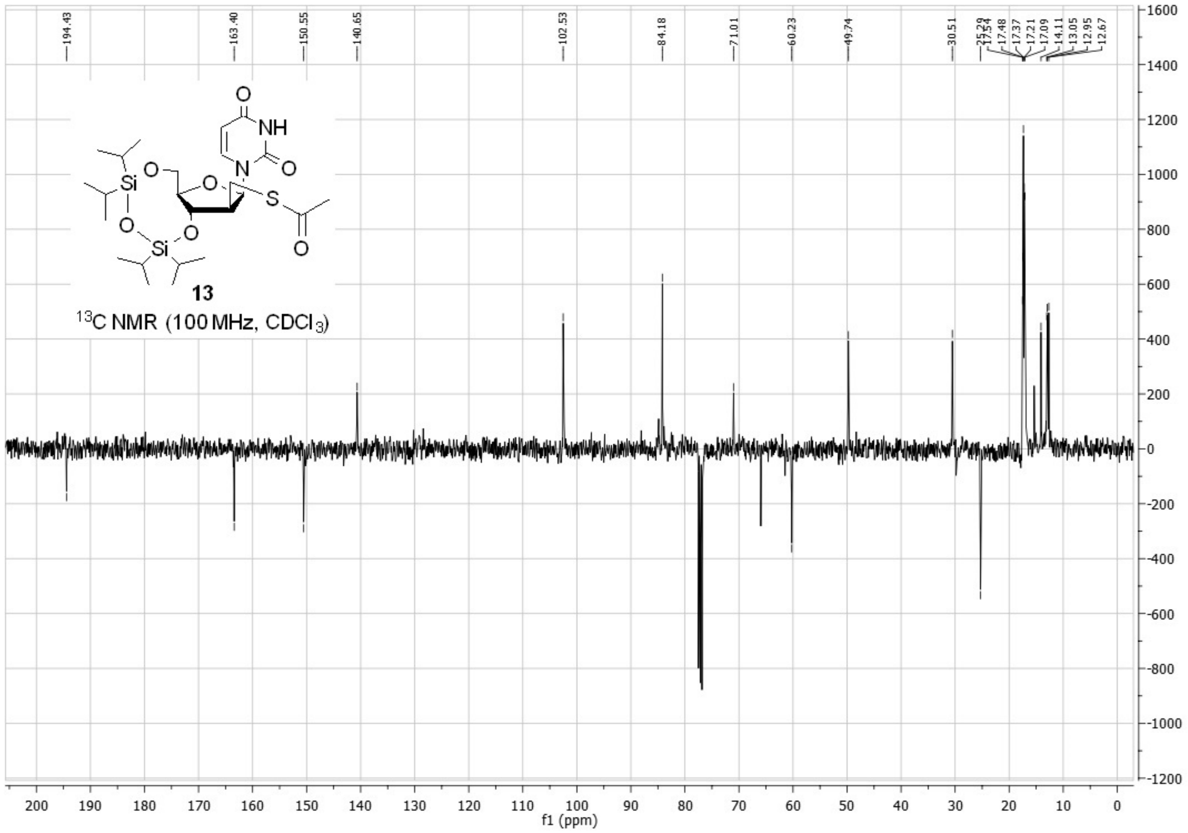
13
^{13}C NMR (100 MHz, CDCl$_3$)
f1 (ppm)

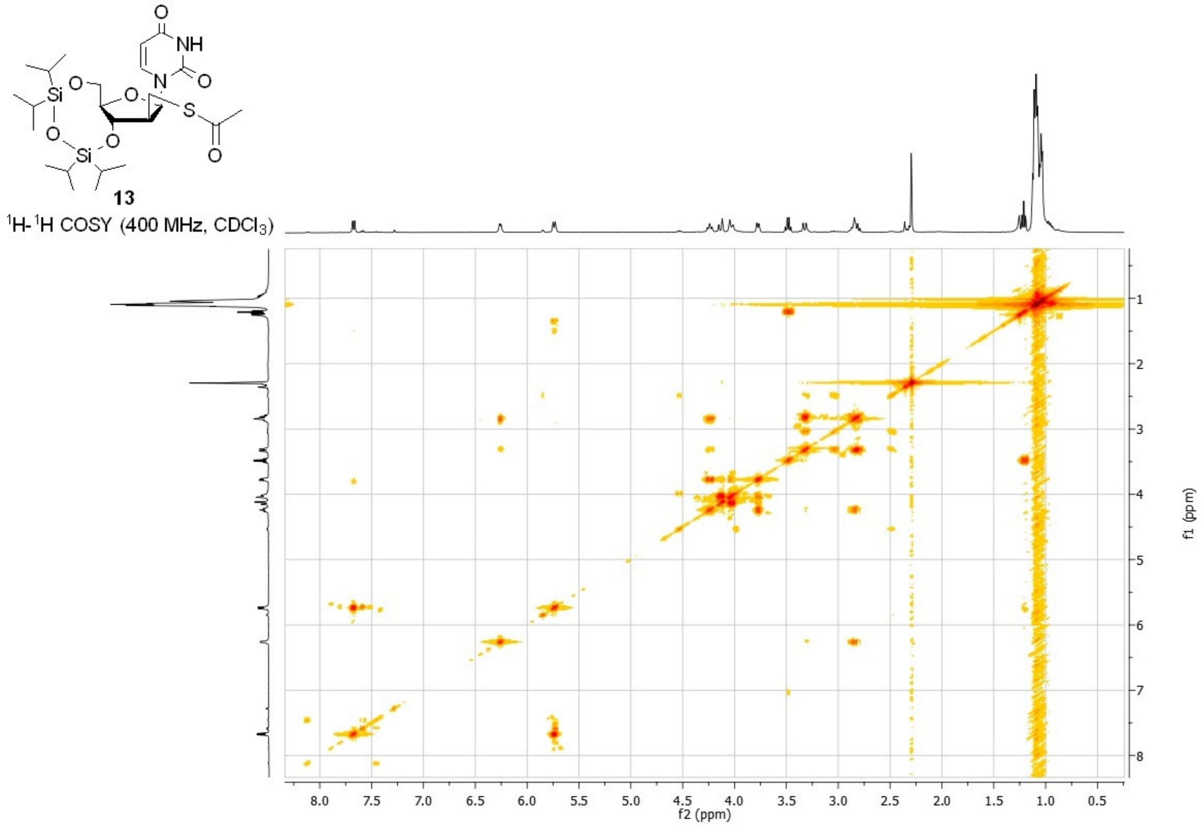
13
^{1}H-^{1}H COSY (400 MHz, CDCl$_3$)
f2 (ppm)
f1 (ppm)

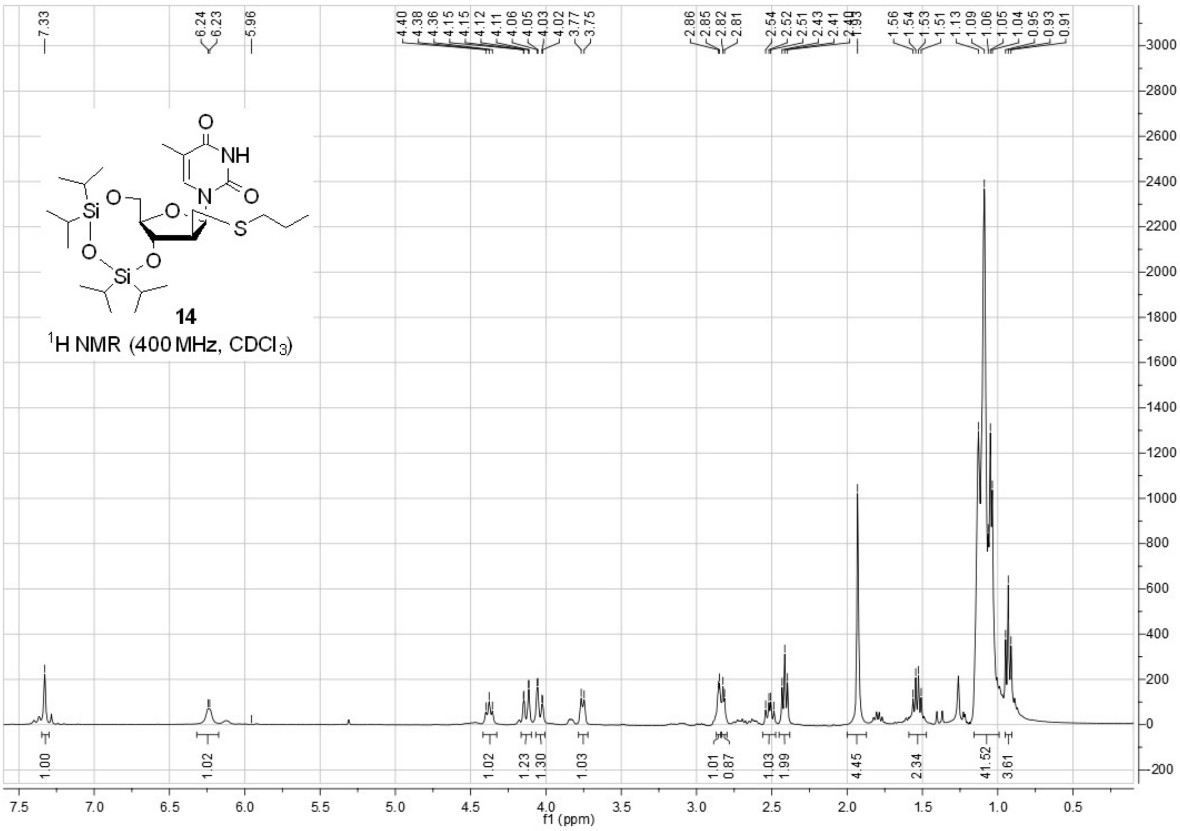

7.33
6.24 6.23
5.96
4.40 4.38 4.36 4.15 4.12 4.11 4.06 4.05 4.03 4.02 3.77 3.75
2.86 2.85 2.82 2.81
2.54 2.52 2.51 2.43 2.41 2.40 1.93
1.56 1.54 1.53 1.51 1.13 1.09 1.06 1.05 1.04 0.95 0.93 0.91
O
NH
N
O
Si
O
O
S
O
O
Si
14
1H NMR (400 MHz, CDCl3)
1.00 1.02 1.02 1.23 1.30 1.03 1.01 0.87 1.03 1.99 4.45 2.34 41.52 3.61
f1 (ppm)

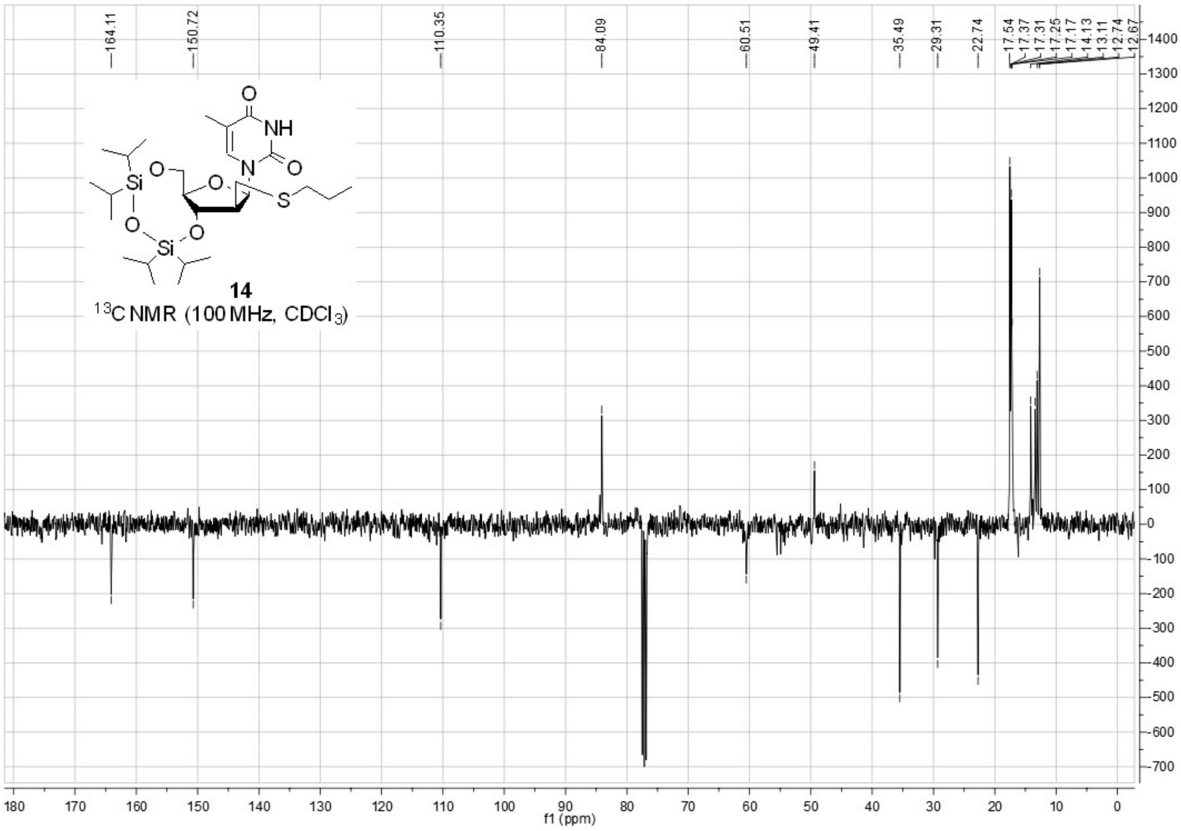

164.11
150.72
110.35
84.09
60.51
49.41
35.49
29.31
22.74
17.54 17.37 17.31 17.25 17.17 14.13 13.11 12.74 12.67
O
NH
N
O
Si
O
O
S
O
O
Si
14
13C NMR (100 MHz, CDCl3)
f1 (ppm)

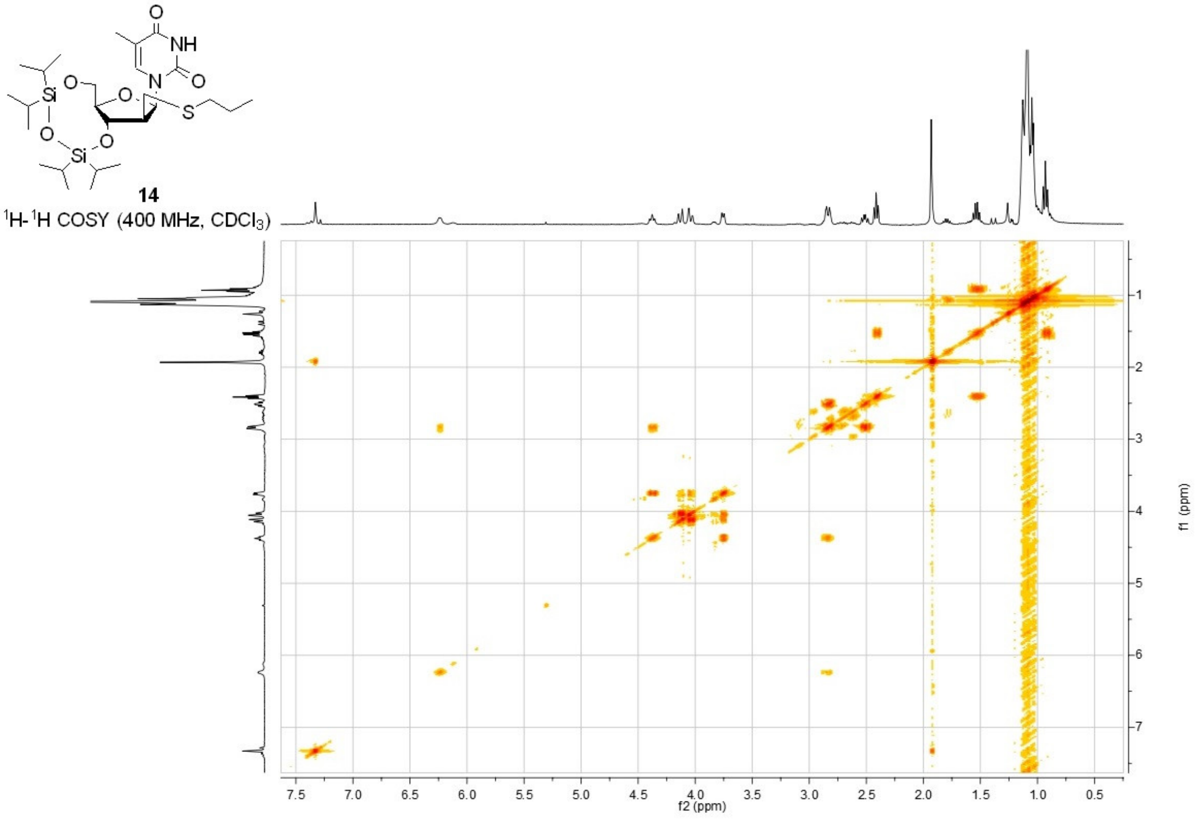

14

^{1}H-^{1}H COSY (400 MHz, $CDCl_3$)

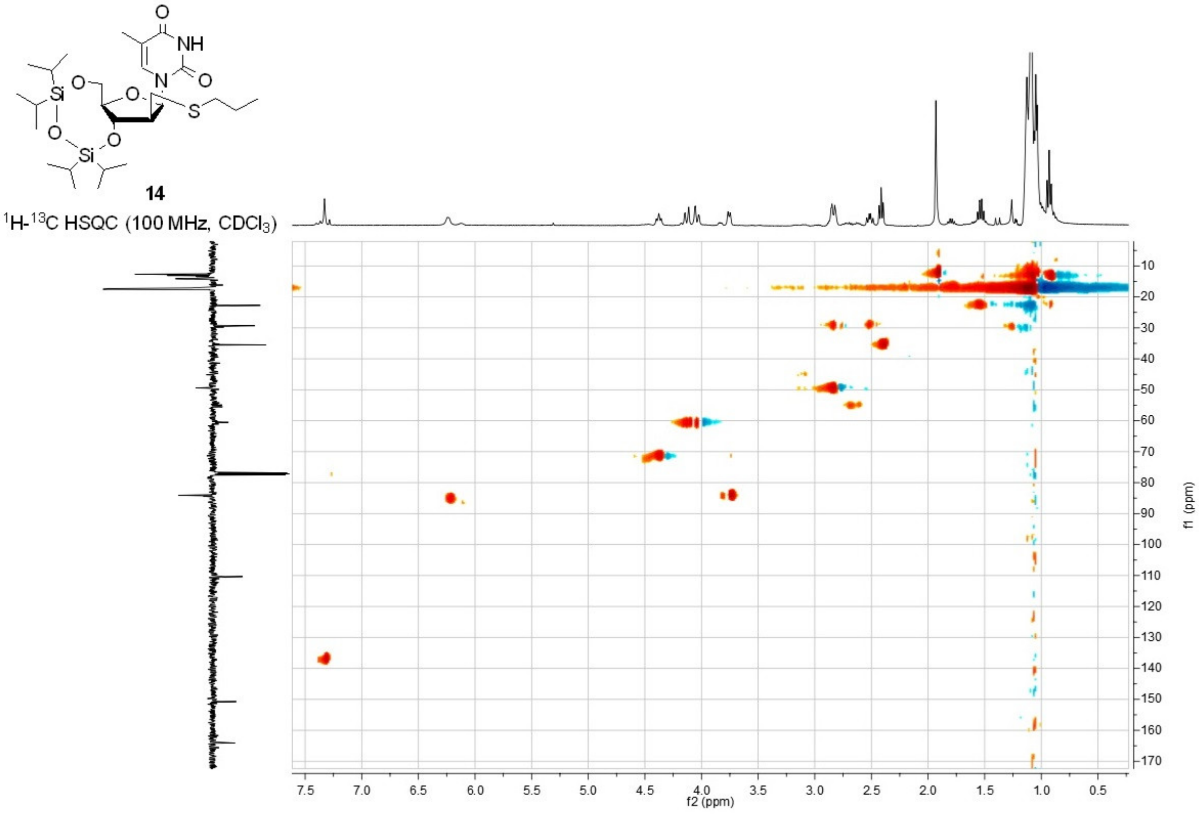

14

^{1}H-^{13}C HSQC (100 MHz, $CDCl_3$)

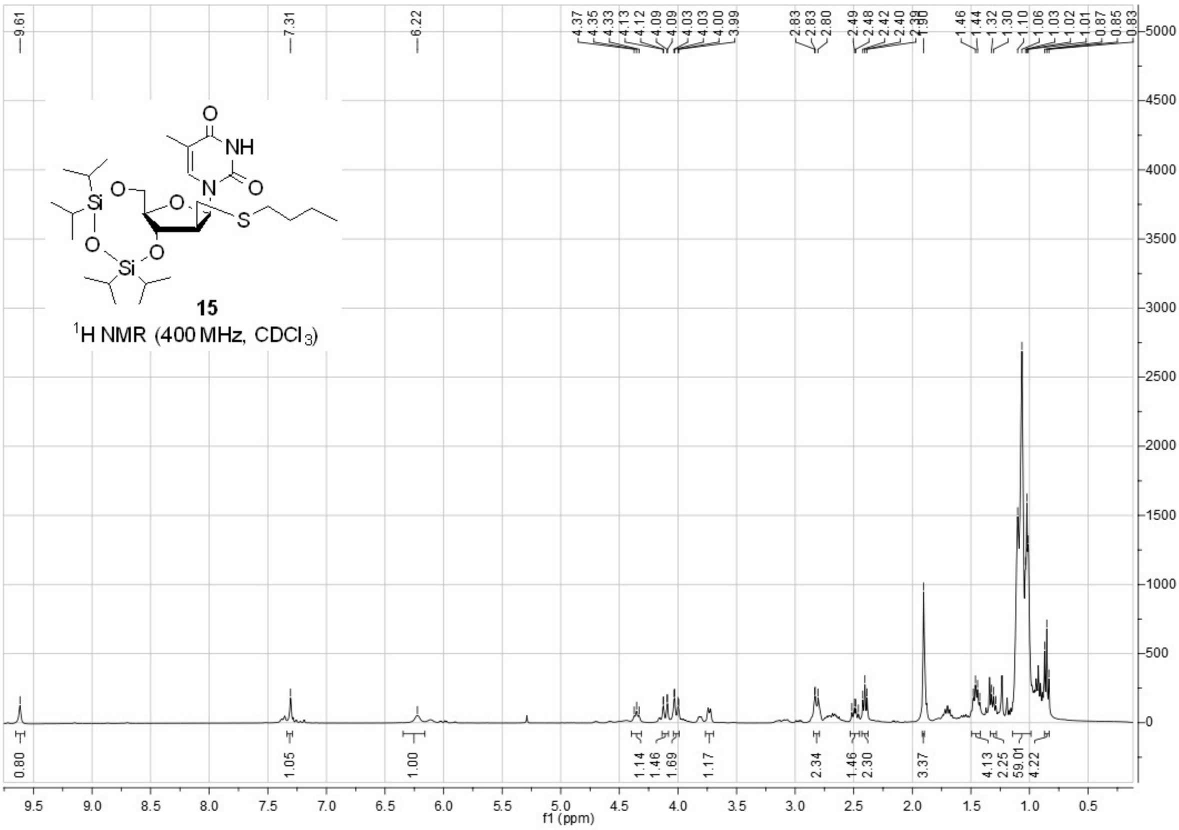
15
^{1}H NMR (400 MHz, CDCl$_3$)
f1 (ppm)

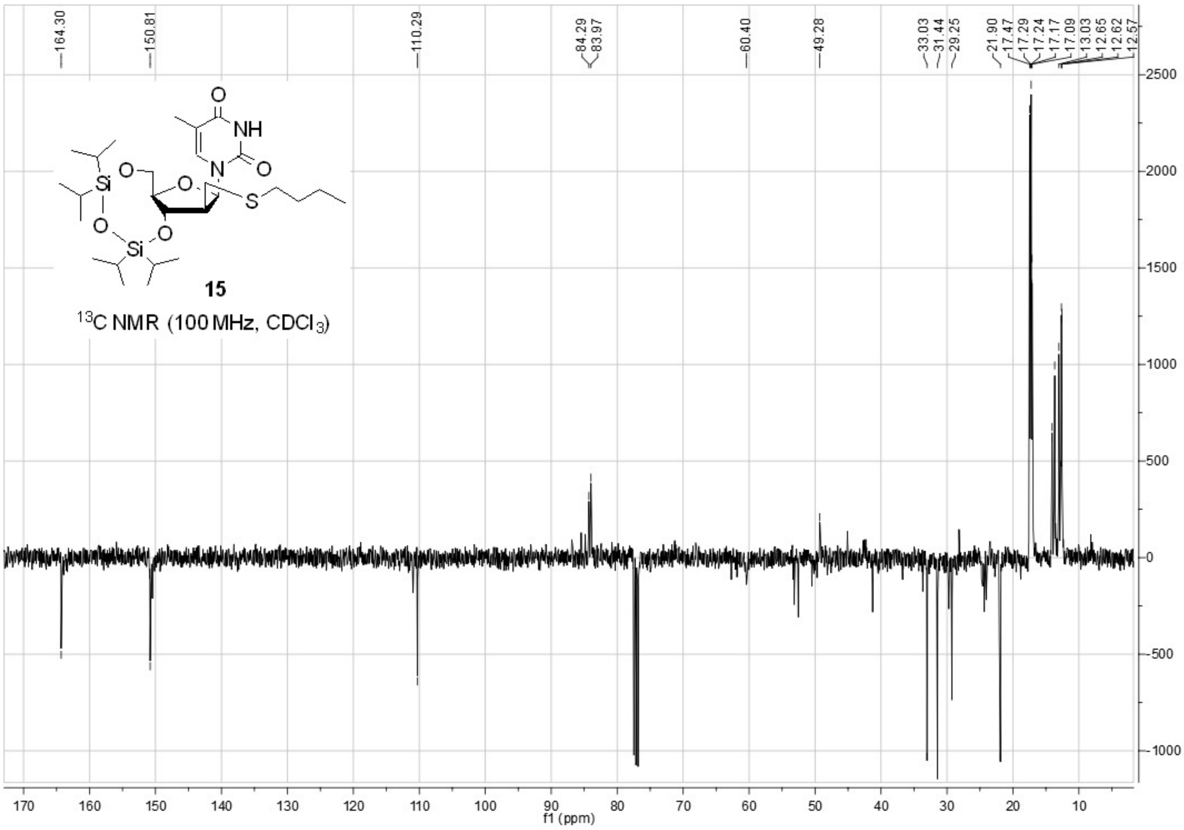
15
^{13}C NMR (100 MHz, CDCl$_3$)
f1 (ppm)

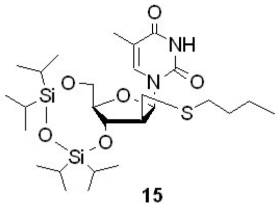

15

^{1}H-^{1}H COSY (400 MHz, $CDCl_3$)

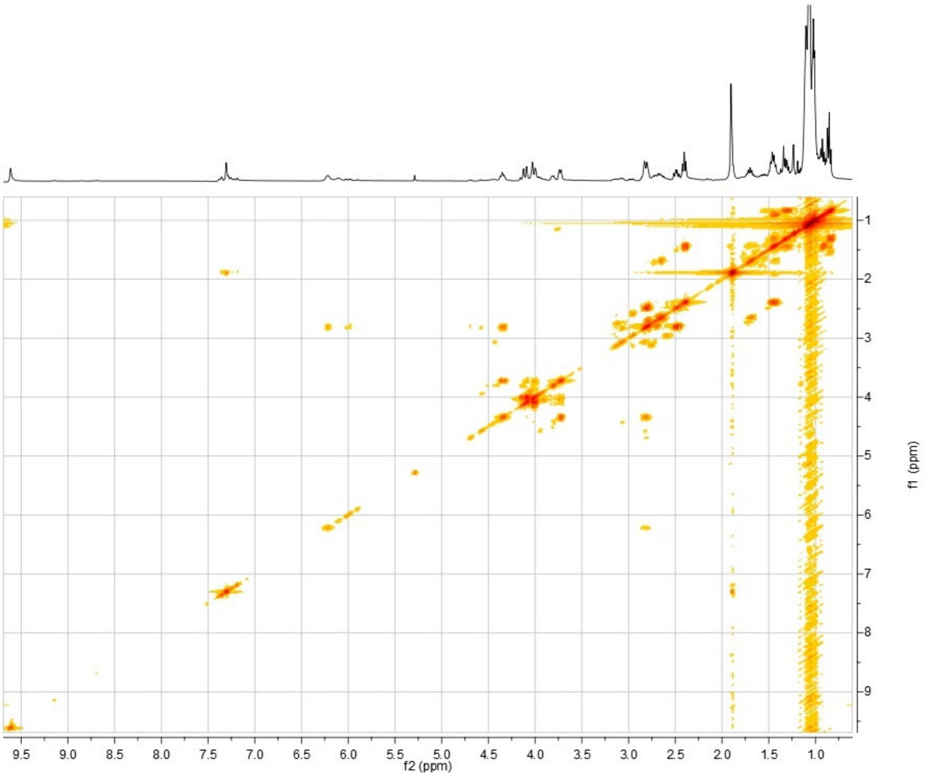

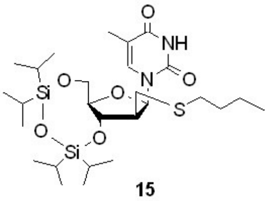

15

^{1}H-^{13}C HSQC (100 MHz, $CDCl_3$)

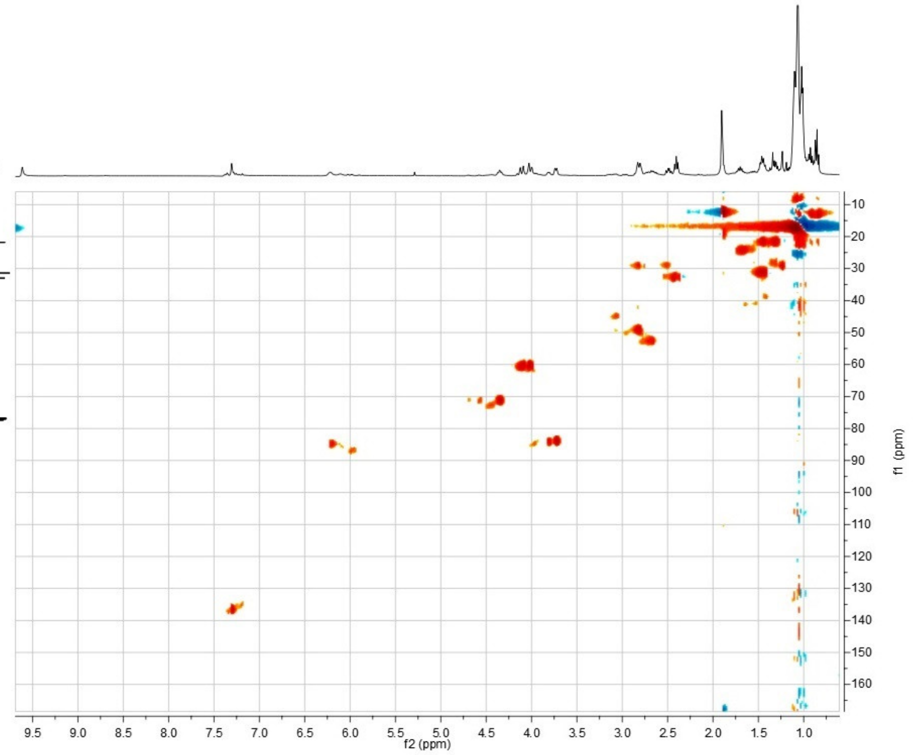

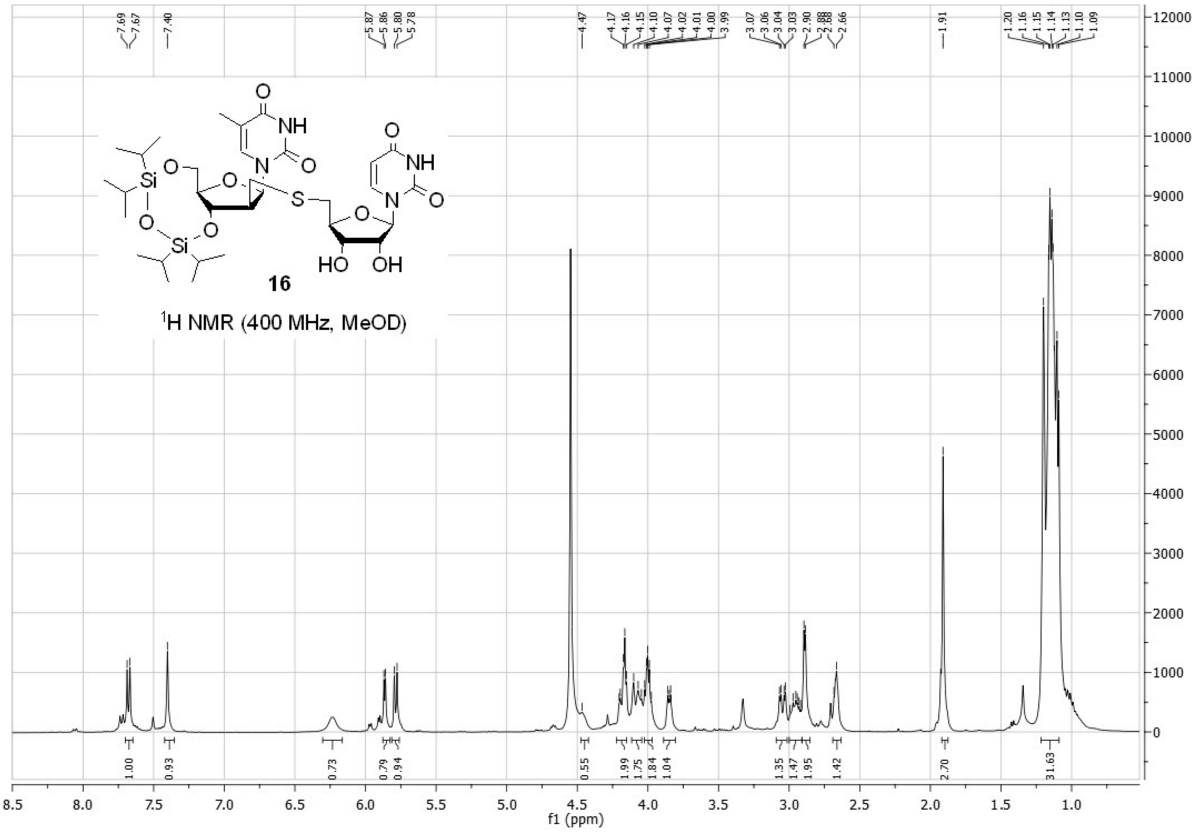
16
^{1}H NMR (400 MHz, MeOD)
f1 (ppm)

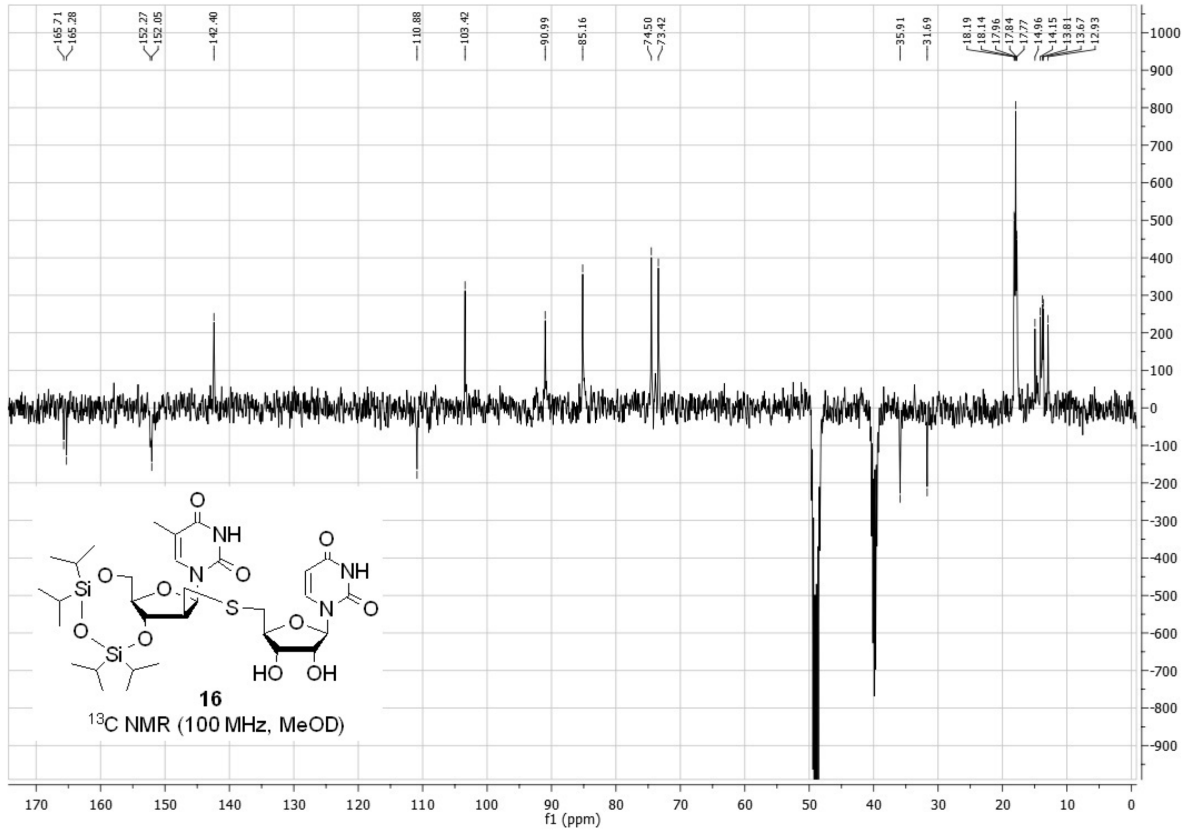
16
^{13}C NMR (100 MHz, MeOD)
f1 (ppm)

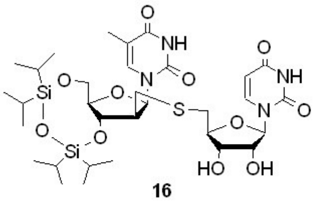

16

^{1}H- ^{1}H COSY (400 MHz, MeOD)

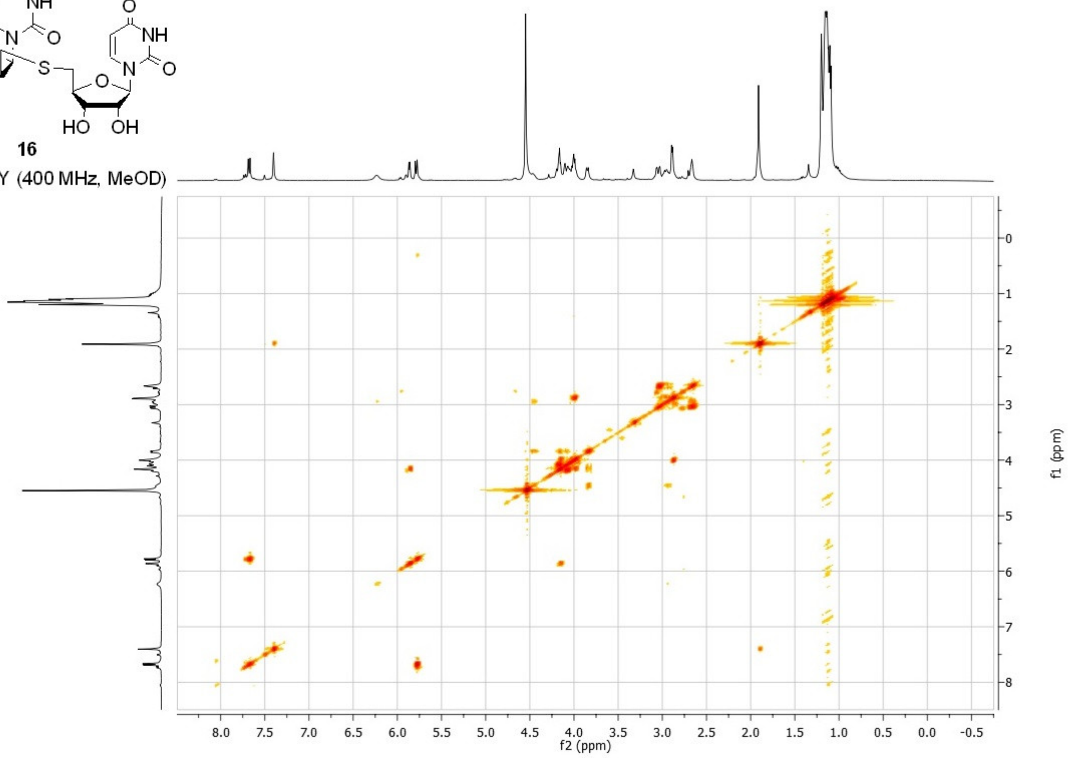

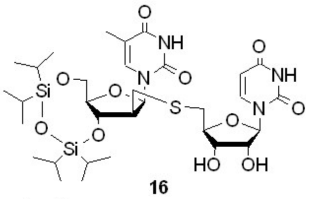

16

^{1}H-^{13}C HSQC (100 MHz, MeOD)

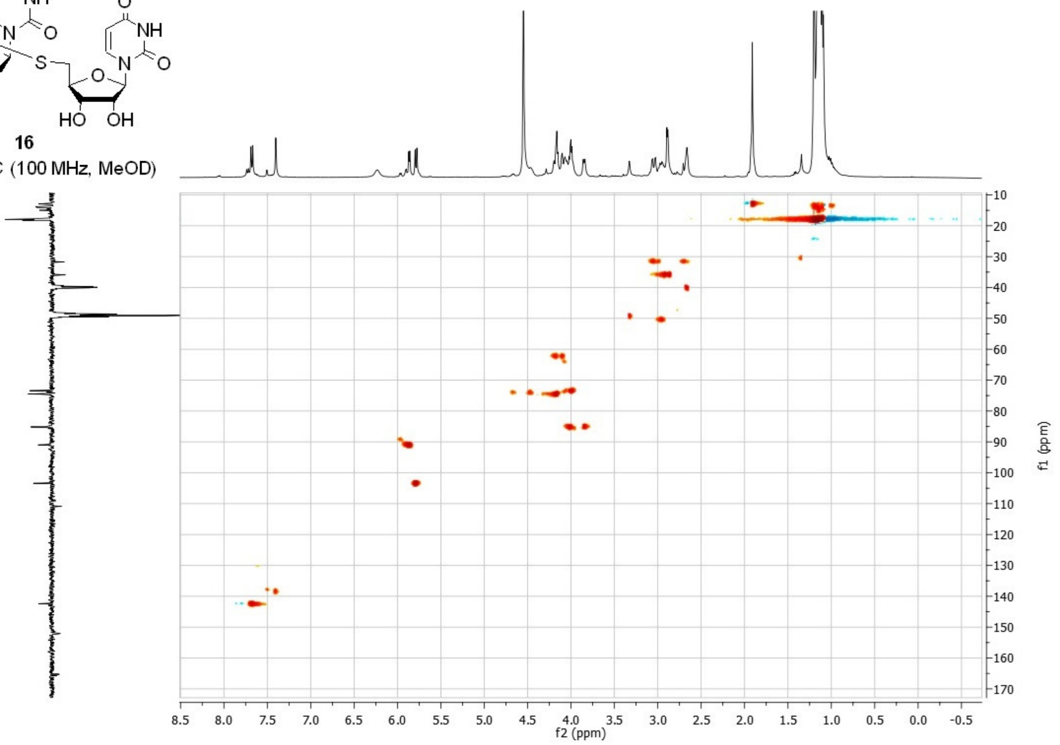

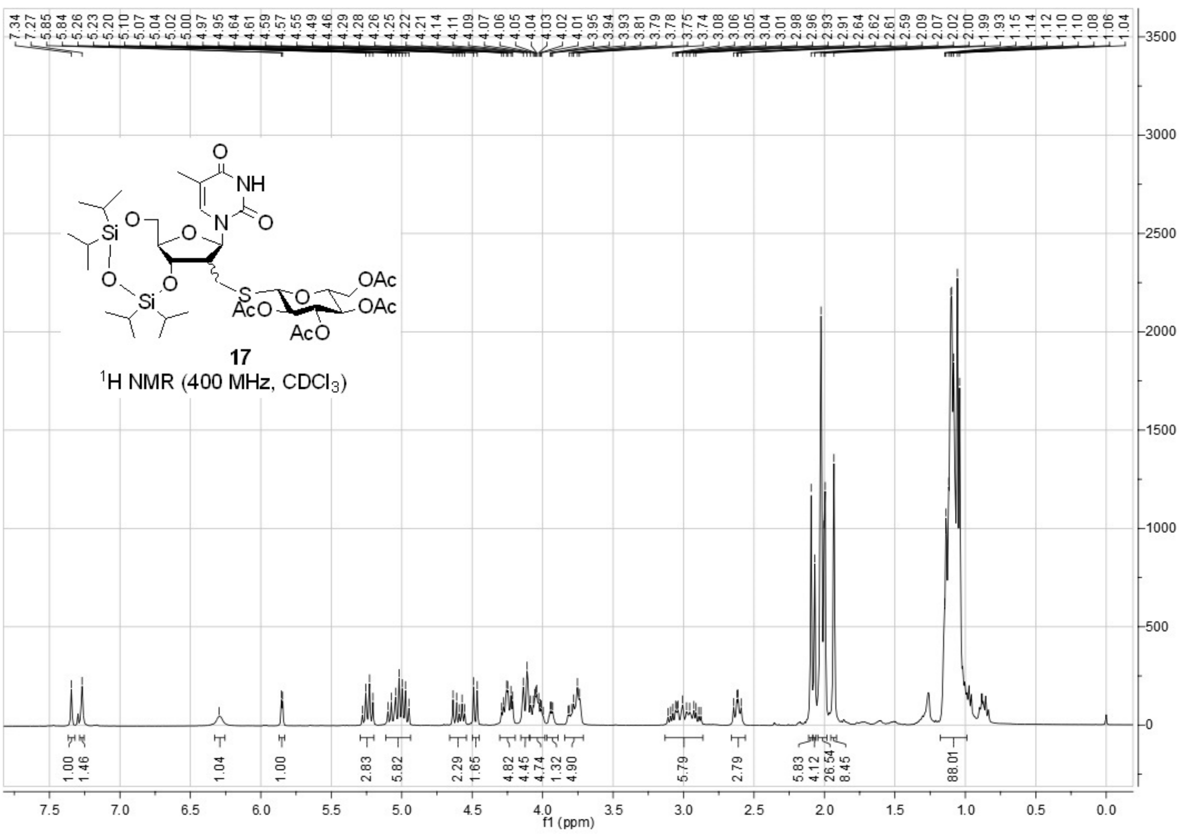

O
NH
N
O
Si
O
O
O
Si
S
OAc
OAc
AcO
AcO
17
^{1}H NMR (400 MHz, CDCl$_3$)
f1 (ppm)

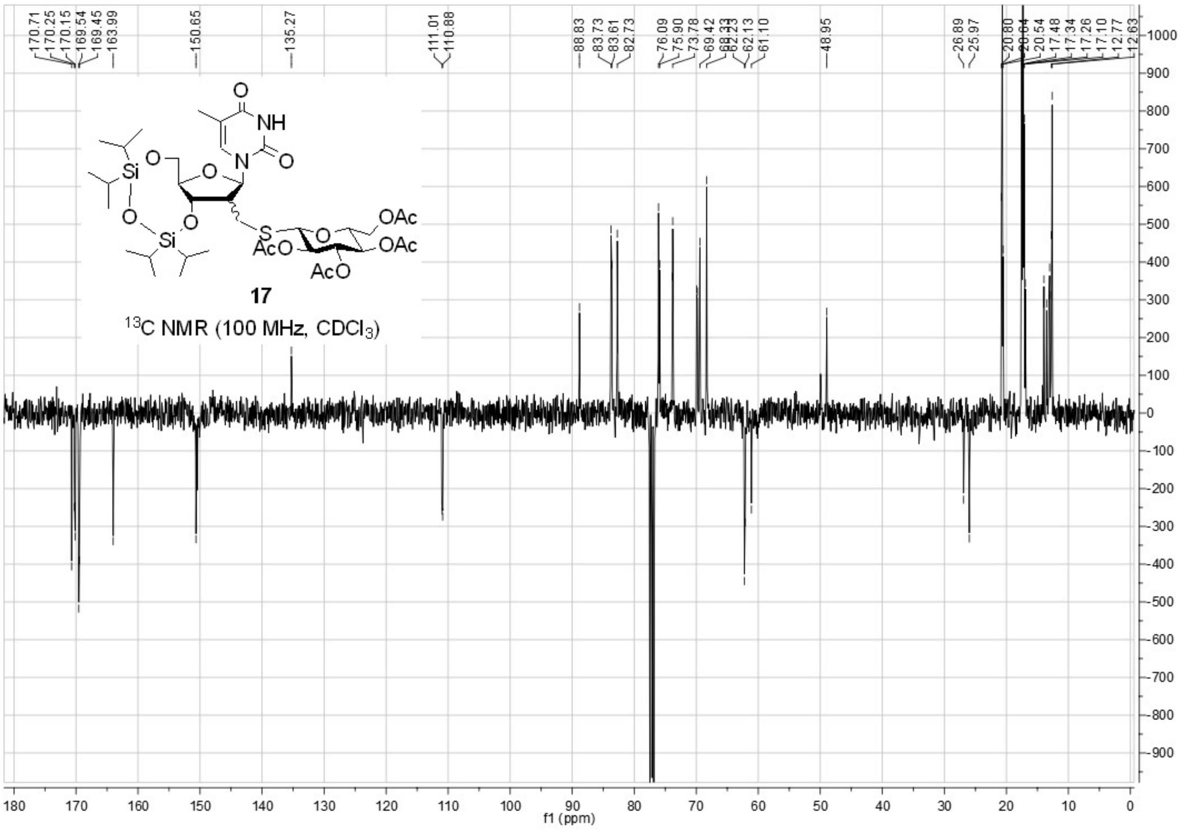

17
^{13}C NMR (100 MHz, CDCl$_3$)
f1 (ppm)

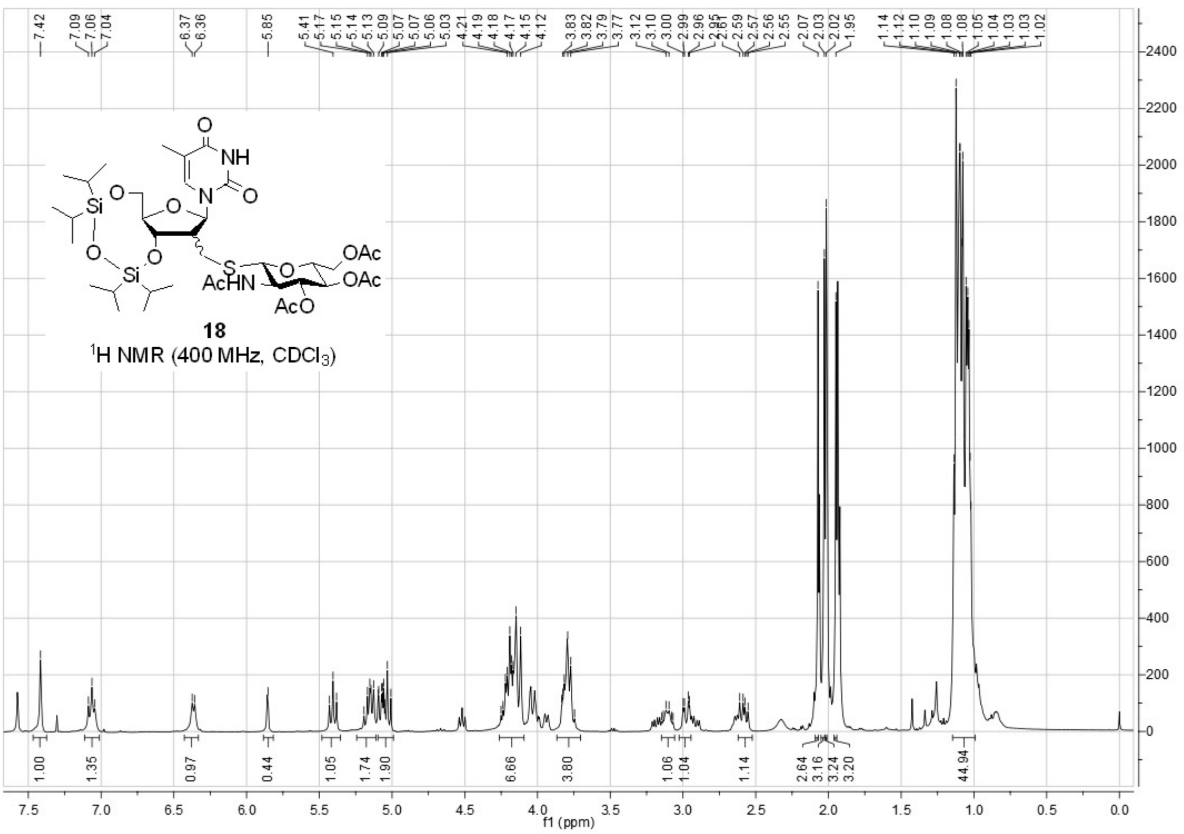
18
1H NMR (400 MHz, CDCl3)
OAc
OAc
AcHN
AcO
f1 (ppm)

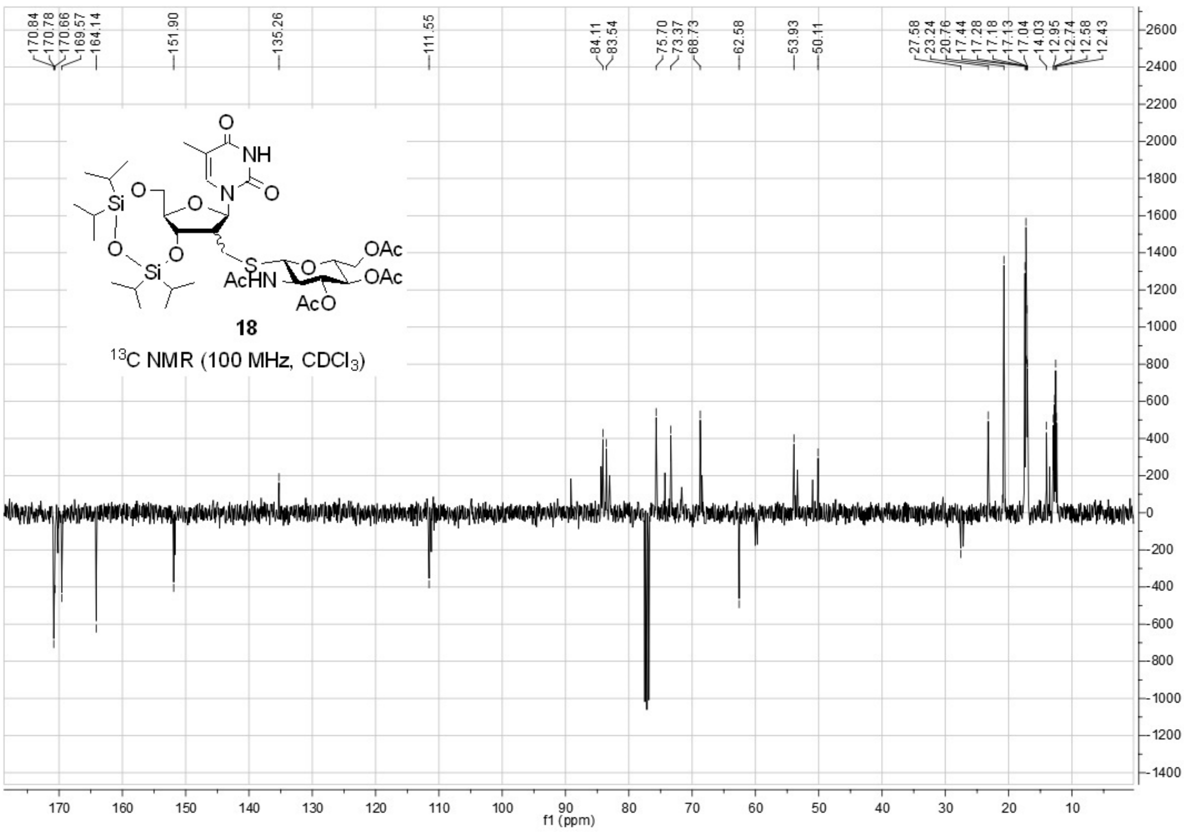
18
13C NMR (100 MHz, CDCl3)
OAc
OAc
AcHN
AcO
f1 (ppm)

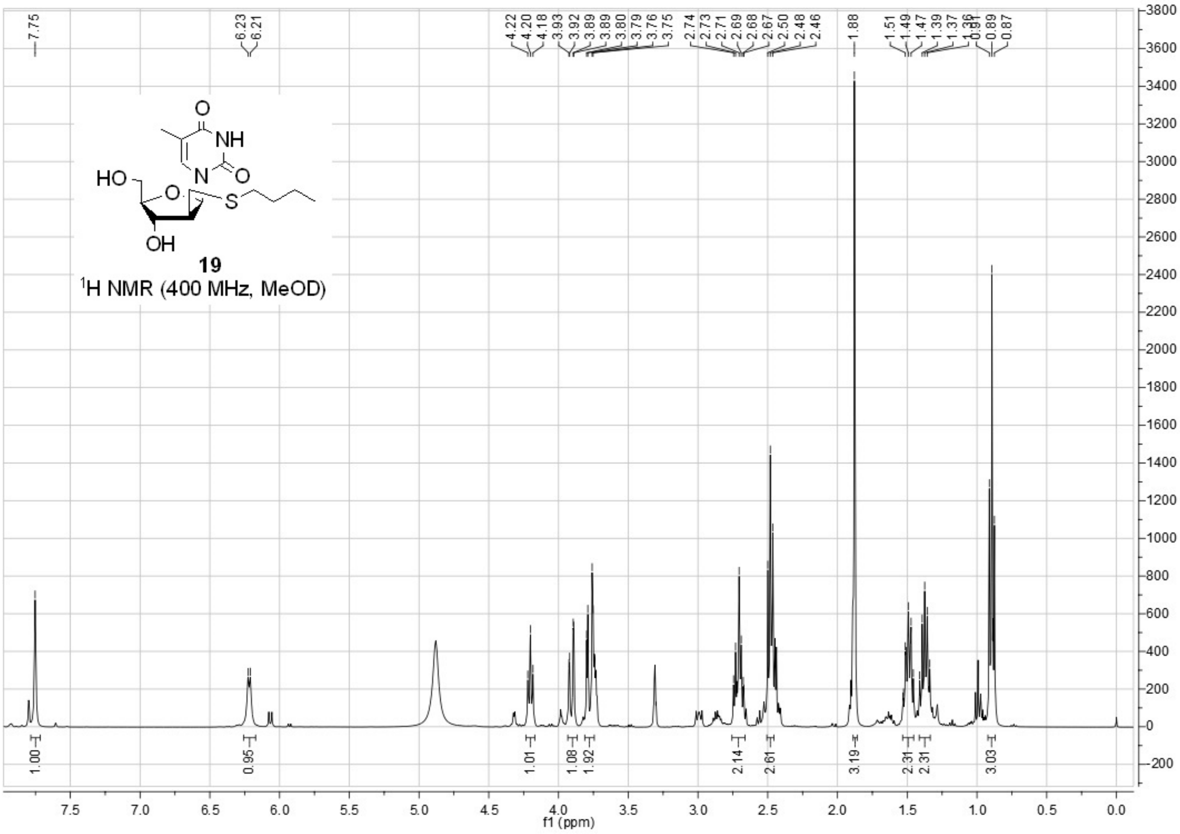
7.75
6.23
6.21
4.22
4.20
4.18
3.93
3.92
3.89
3.89
3.80
3.79
3.76
3.75
2.74
2.73
2.71
2.69
2.68
2.67
2.50
2.48
2.46
1.88
1.51
1.49
1.47
1.39
1.37
0.89
0.87
O
NH
HO
N
O
O
S
OH
19
1H NMR (400 MHz, MeOD)
1.00
0.95
1.01
1.08
1.92
2.14
2.61
3.19
2.31
2.31
3.03
f1 (ppm)

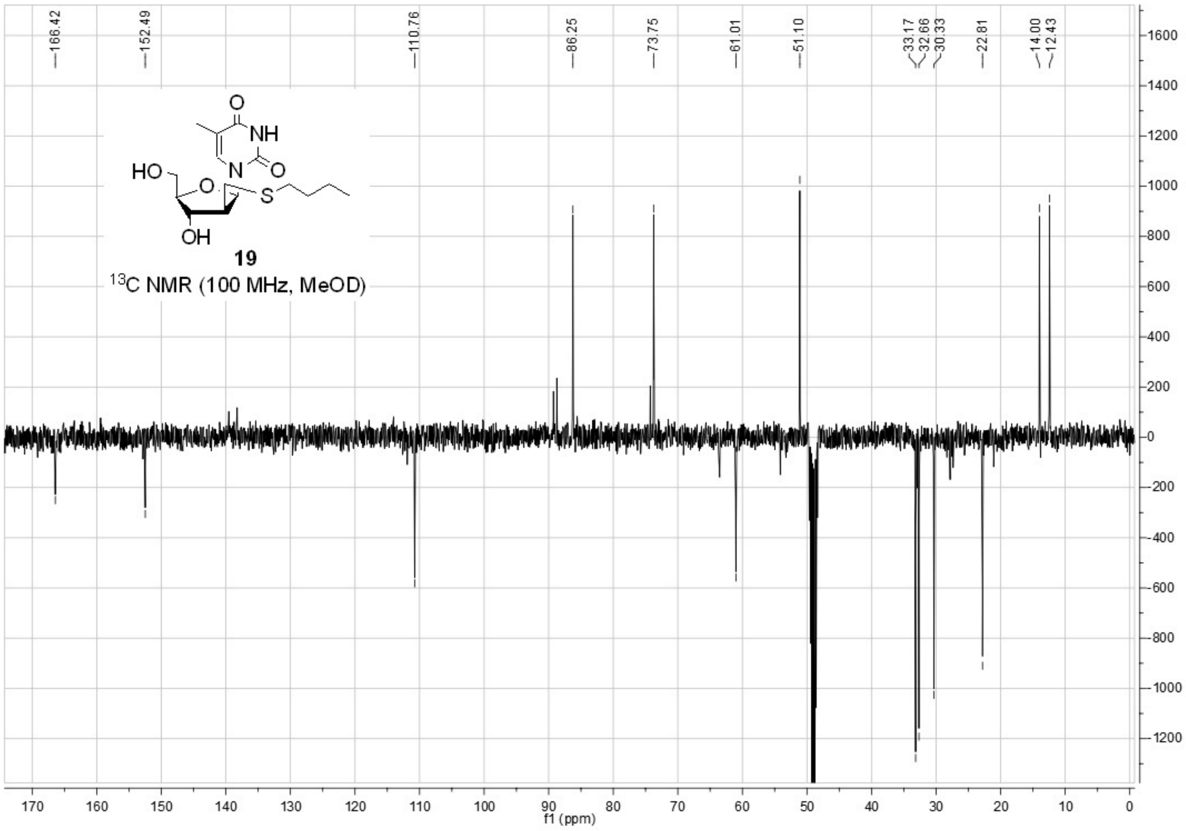
166.42
152.49
110.76
86.25
73.75
61.01
51.10
33.17
32.66
30.33
22.81
14.00
12.43
O
NH
HO
N
O
O
S
OH
19
13C NMR (100 MHz, MeOD)
f1 (ppm)

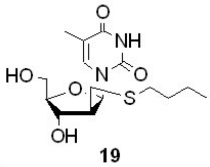

19

^{1}H-^{1}H COSY (400 MHz, MeOD)

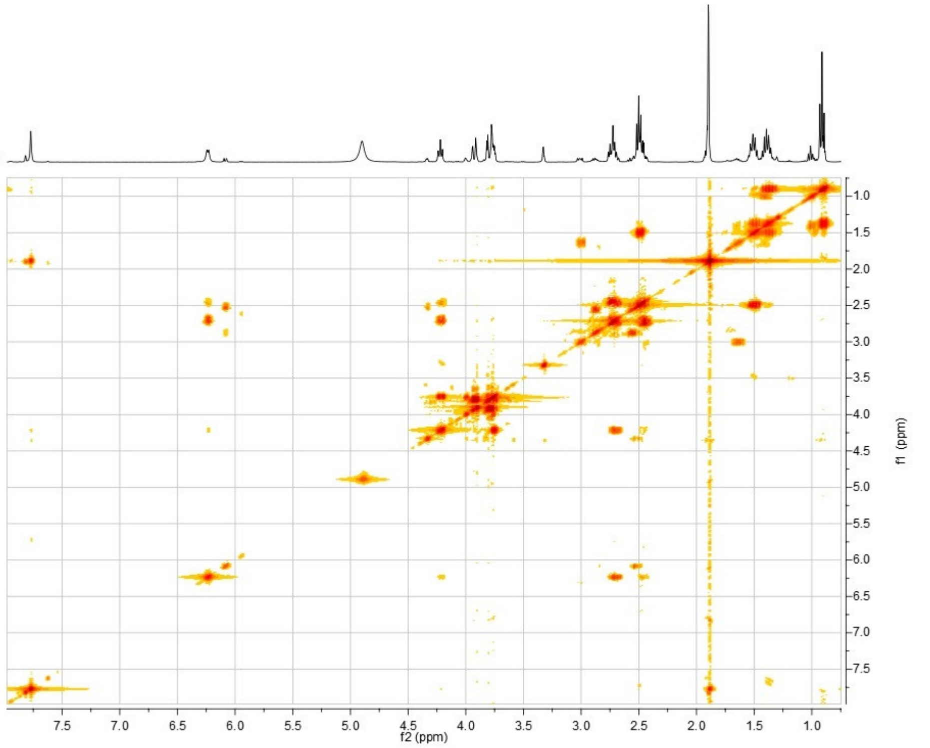

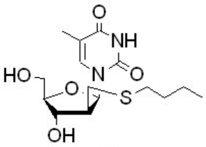

19

^{1}H-^{13}C HSQC (100 MHz, MeOD)

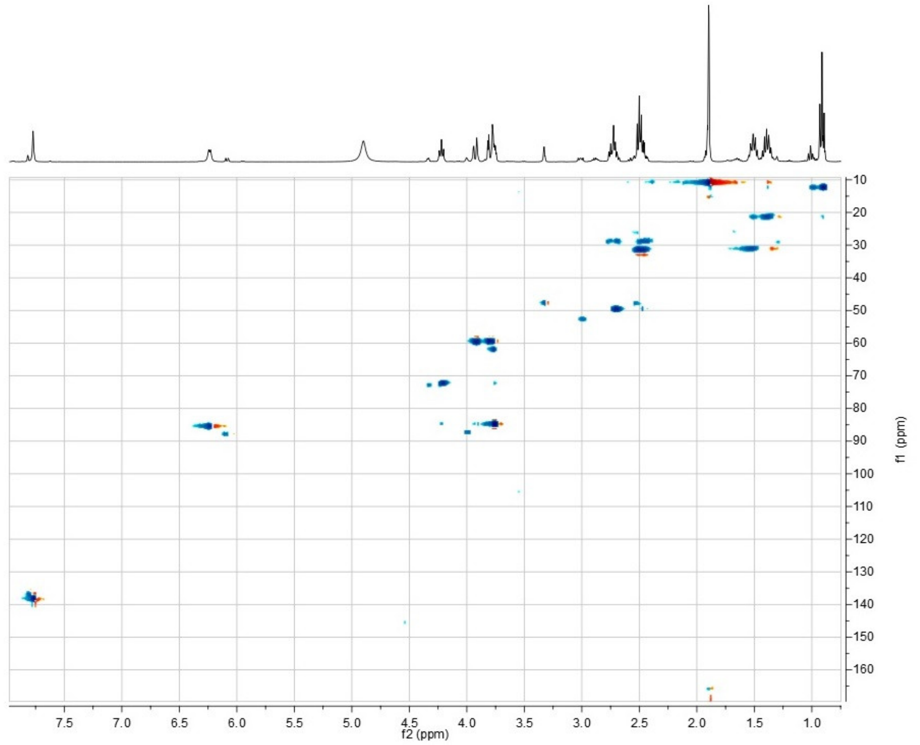

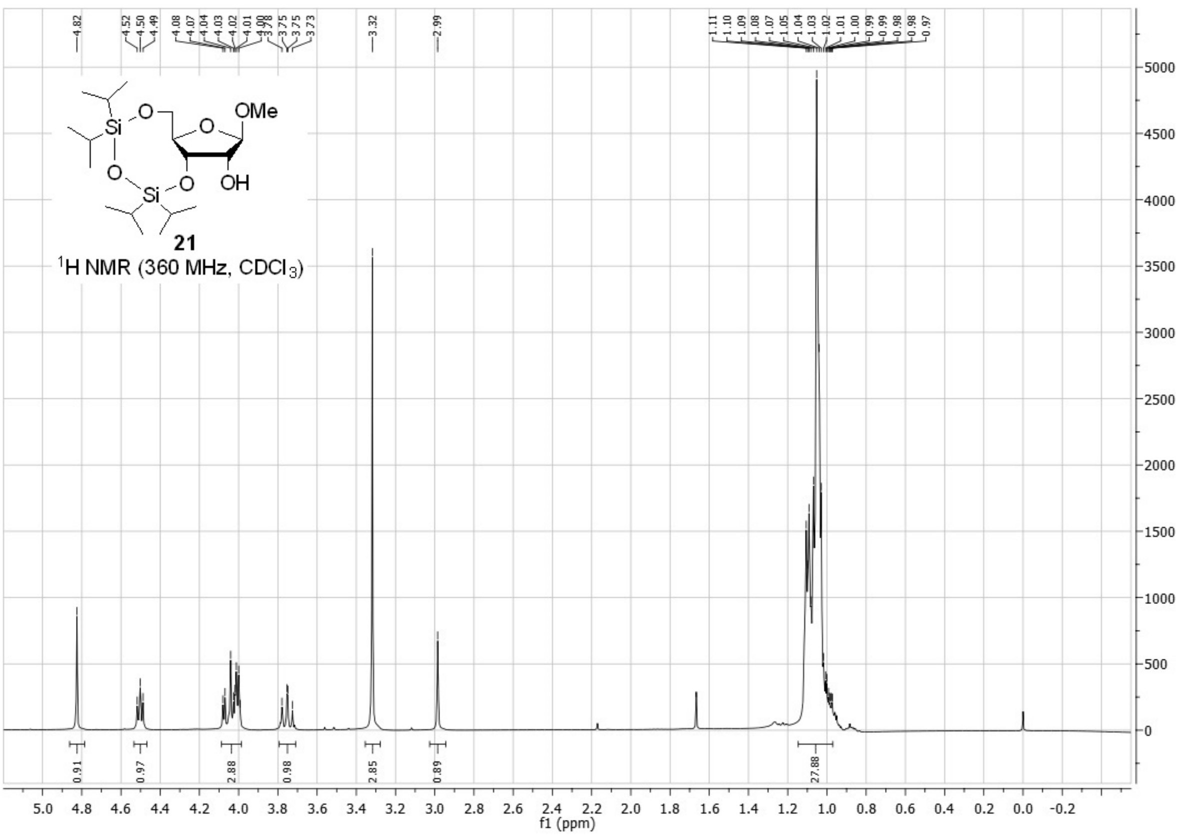

4.82
4.52
4.50
4.49
4.08
4.07
4.04
4.03
4.02
4.01
3.78
3.75
3.75
3.73
3.32
2.99
1.11
1.10
1.09
1.08
1.07
1.05
1.04
1.03
1.02
1.01
1.00
0.99
0.99
0.98
0.98
0.97
OMe
Si
O
OH
21
^{1}H NMR (360 MHz, CDCl$_3$)
0.91
0.97
2.88
0.98
2.85
0.89
27.88
f1 (ppm)

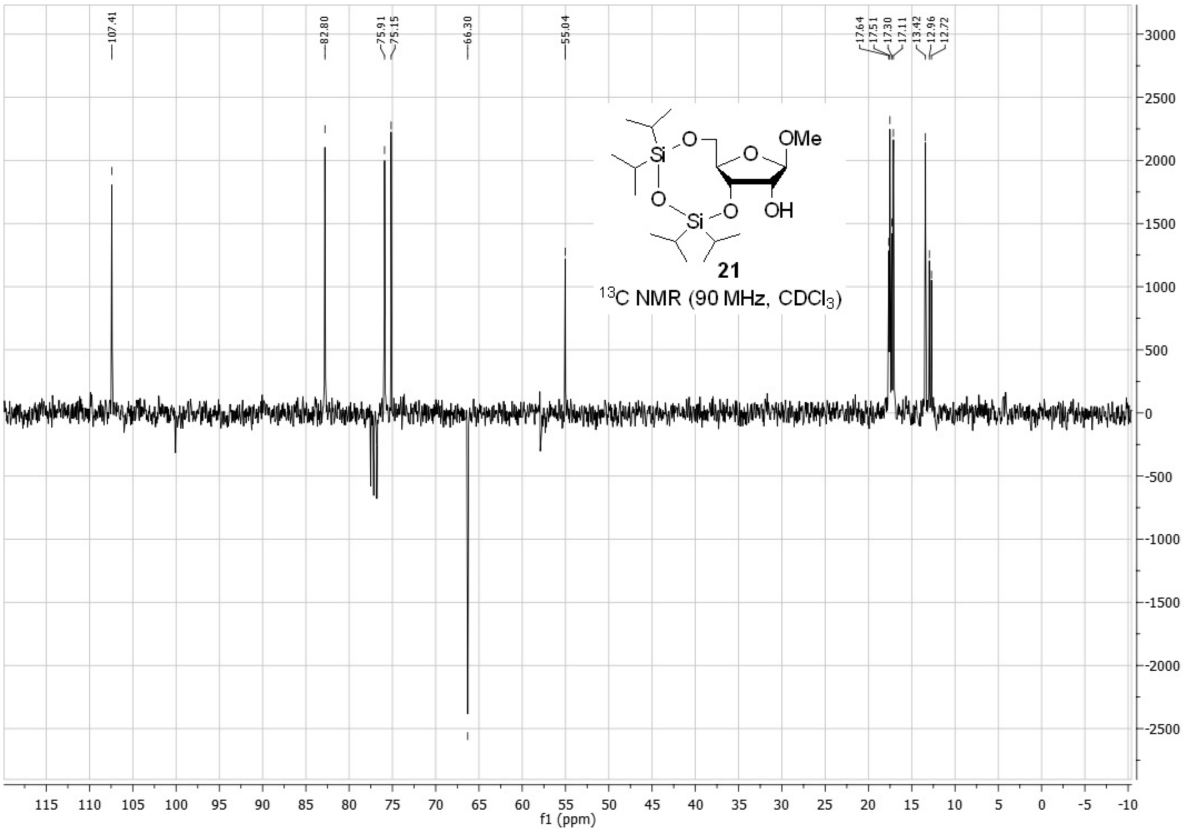

107.41
82.80
75.91
75.15
66.30
55.04
17.64
17.51
17.30
17.11
13.42
12.96
12.72
OMe
Si
O
OH
21
^{13}C NMR (90 MHz, CDCl$_3$)
f1 (ppm)

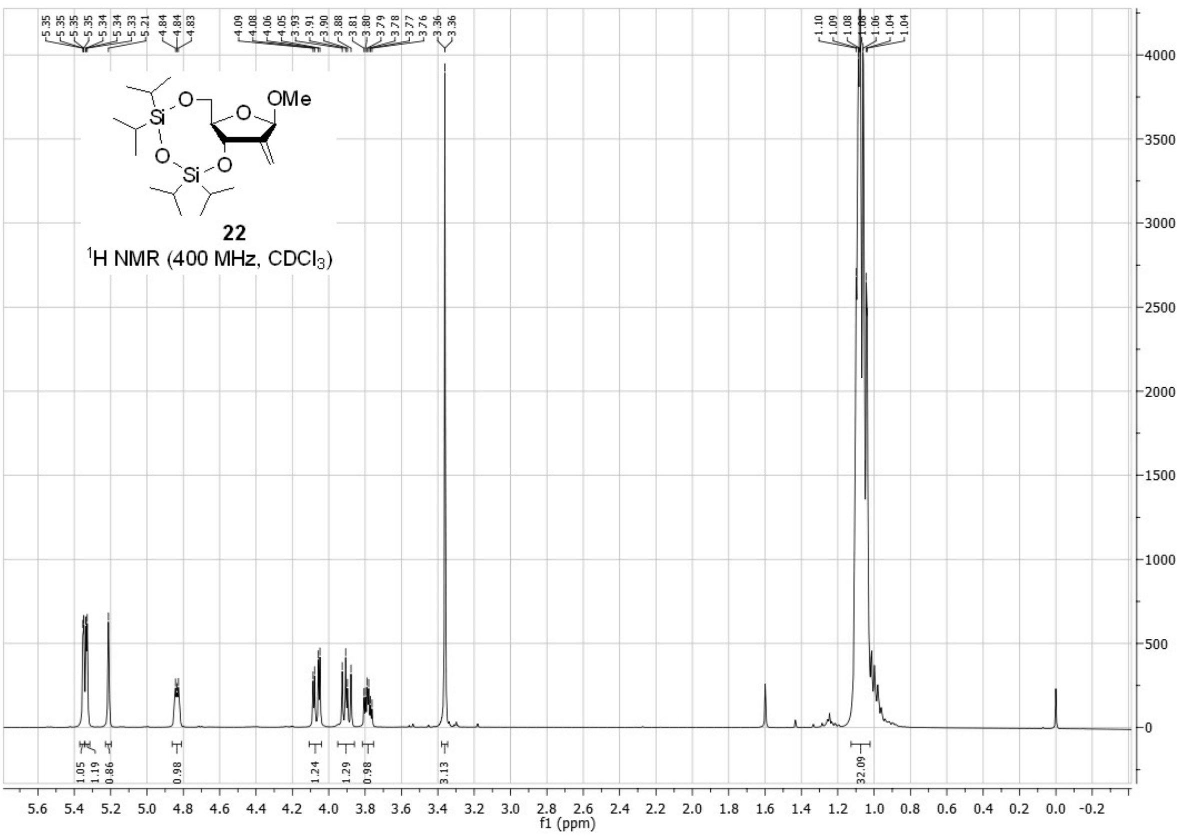

OMe
Si
O
22
^{1}H NMR (400 MHz, $CDCl_3$)
f1 (ppm)

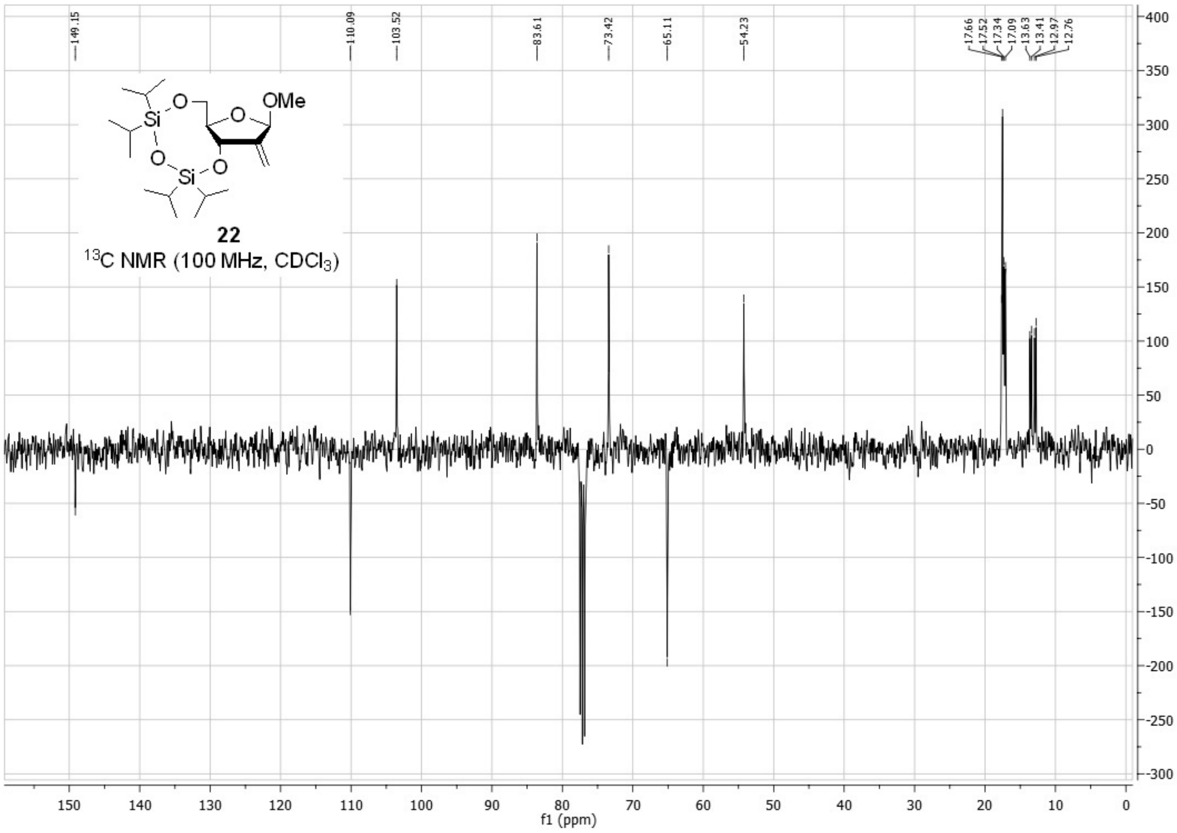

OMe
Si
O
22
^{13}C NMR (100 MHz, $CDCl_3$)
f1 (ppm)

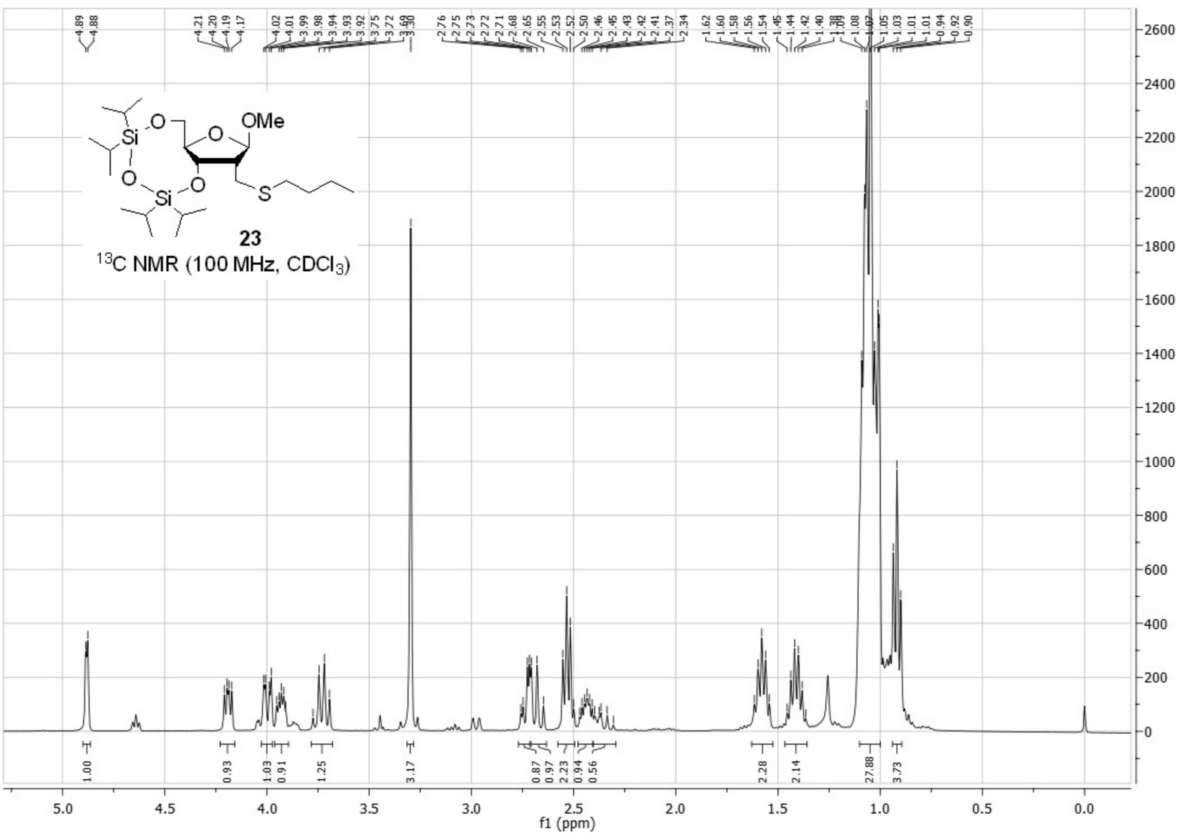

OMe
Si
O
S
23
^{13}C NMR (100 MHz, CDCl$_3$)
4.89 4.88 4.21 4.20 4.19 4.17 4.02 4.01 3.99 3.98 3.94 3.93 3.92 3.75 3.72 3.69 3.30 2.76 2.75 2.73 2.72 2.71 2.68 2.65 2.55 2.53 2.52 2.50 2.46 2.45 2.43 2.42 2.41 2.37 2.34 1.62 1.60 1.58 1.56 1.54 1.45 1.44 1.42 1.40 1.38 1.08 1.07 1.05 1.03 1.01 1.01 0.94 0.92 0.90
1.00 0.93 1.03 0.91 1.25 3.17 0.87 0.97 2.23 0.94 0.56 2.28 2.14 27.88 3.73
f1 (ppm)

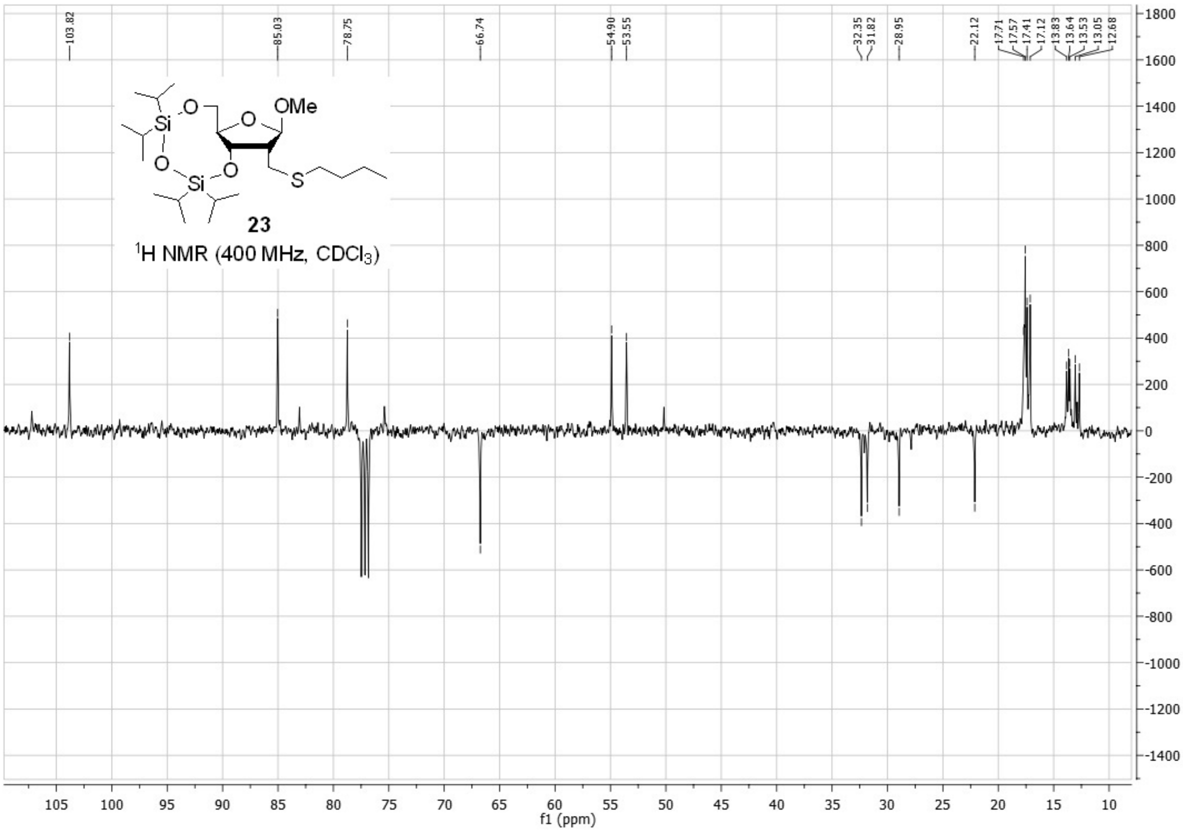

OMe
Si
O
S
23
^{1}H NMR (400 MHz, CDCl$_3$)
103.82 85.03 78.75 66.74 54.90 53.55 32.35 31.82 28.95 22.12 17.71 17.57 17.41 17.12 13.83 13.64 13.53 13.05 12.68
f1 (ppm)

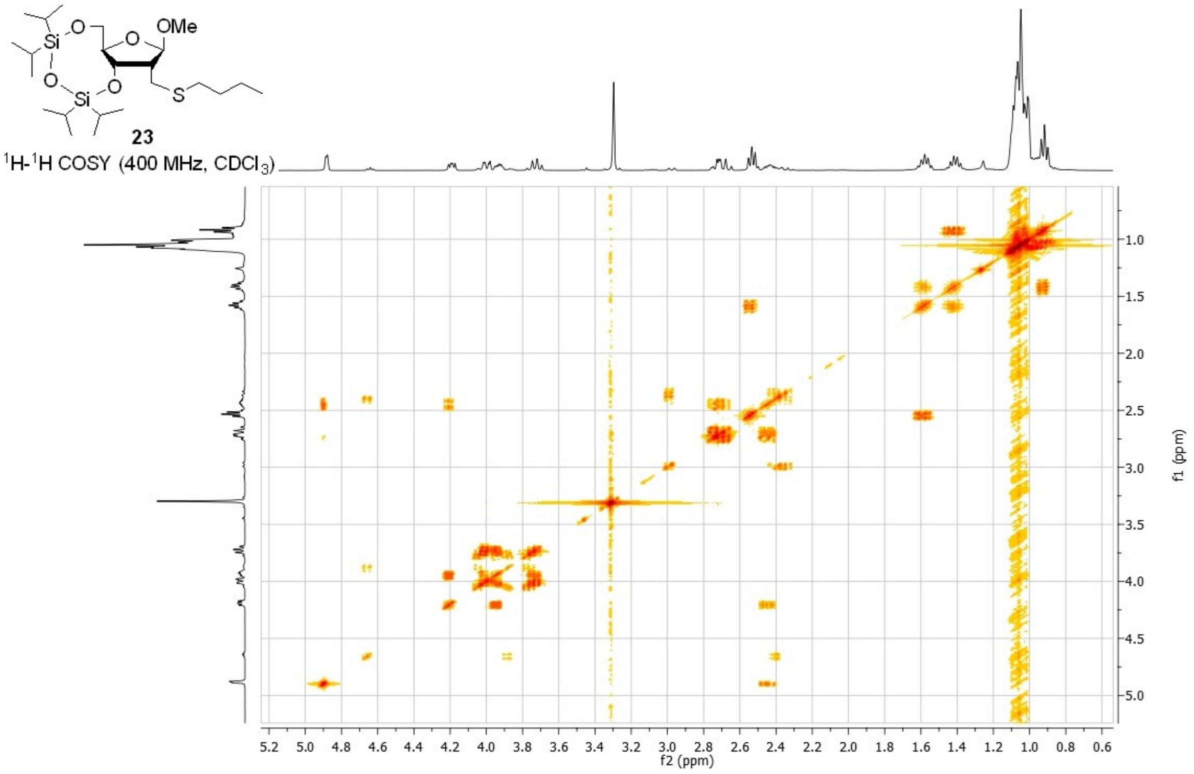

23
^{1}H-^{1}H COSY (400 MHz, CDCl$_3$)

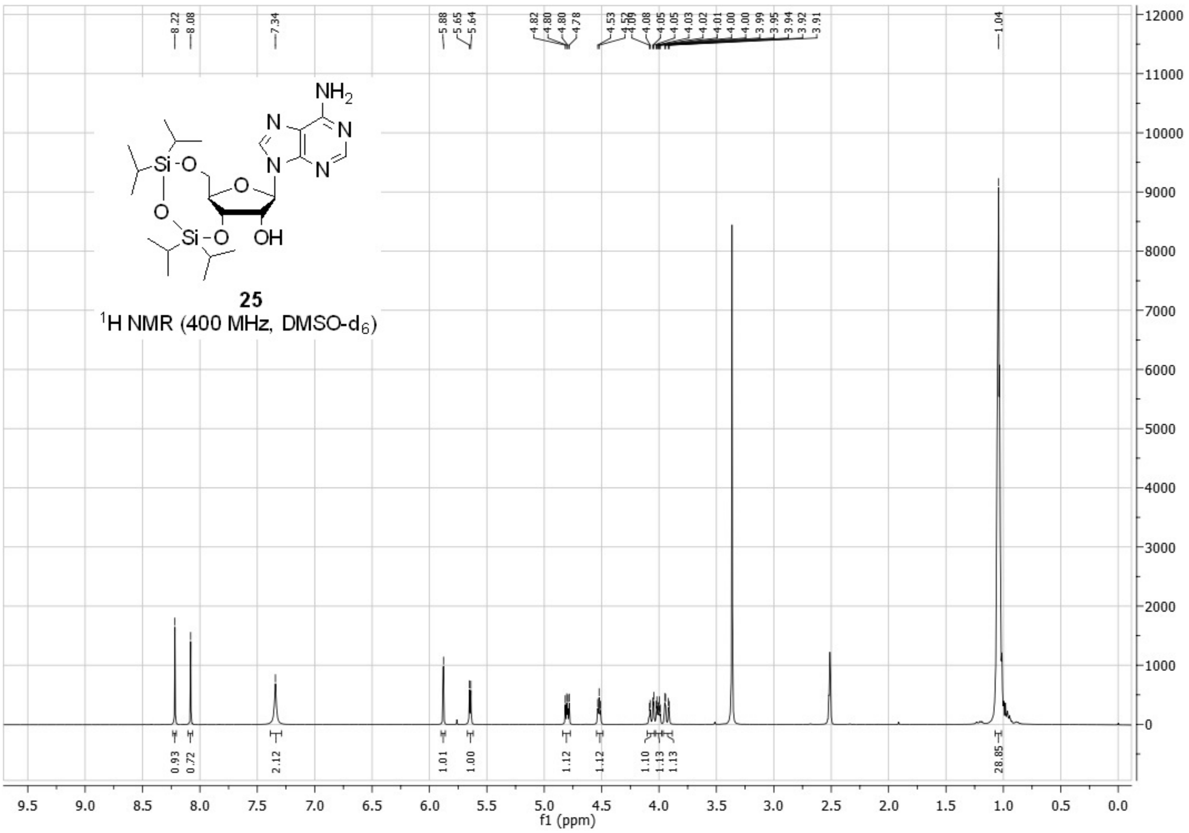

25
^{1}H NMR (400 MHz, DMSO-d$_6$)

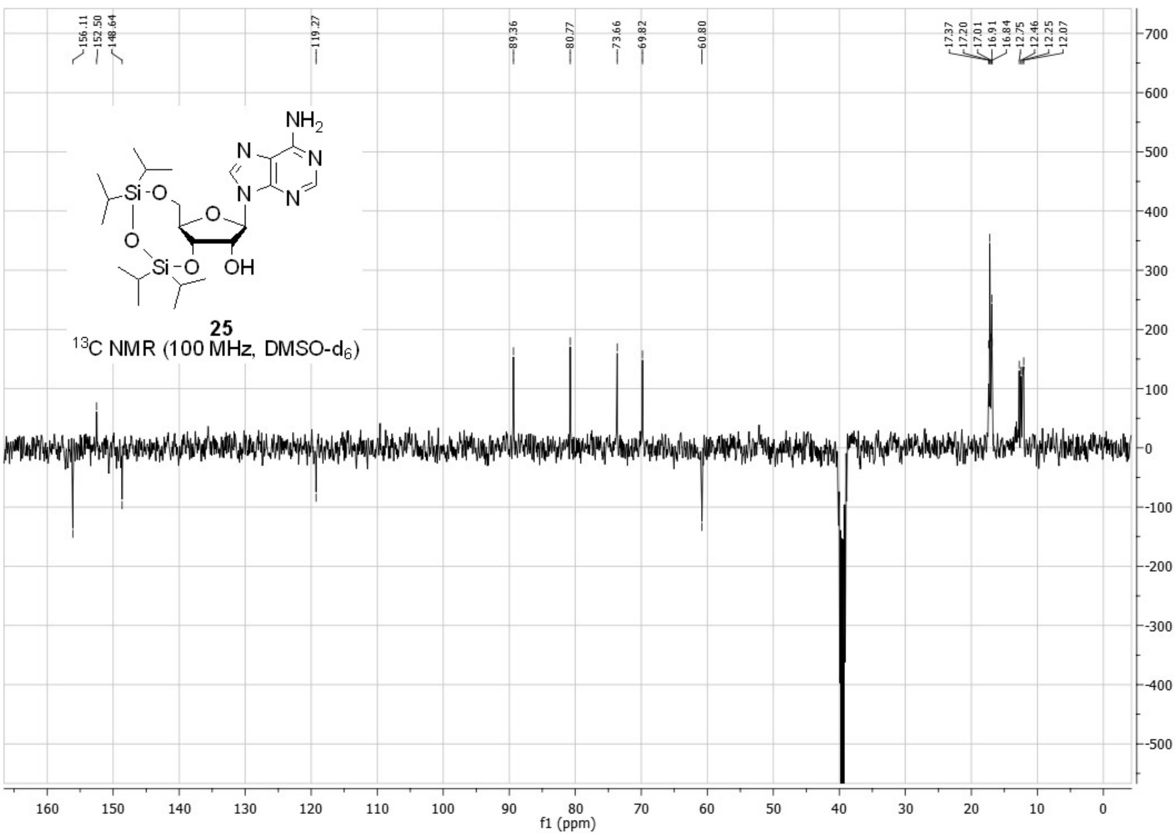
25
^{13}C NMR (100 MHz, DMSO-d$_6$)
f1 (ppm)

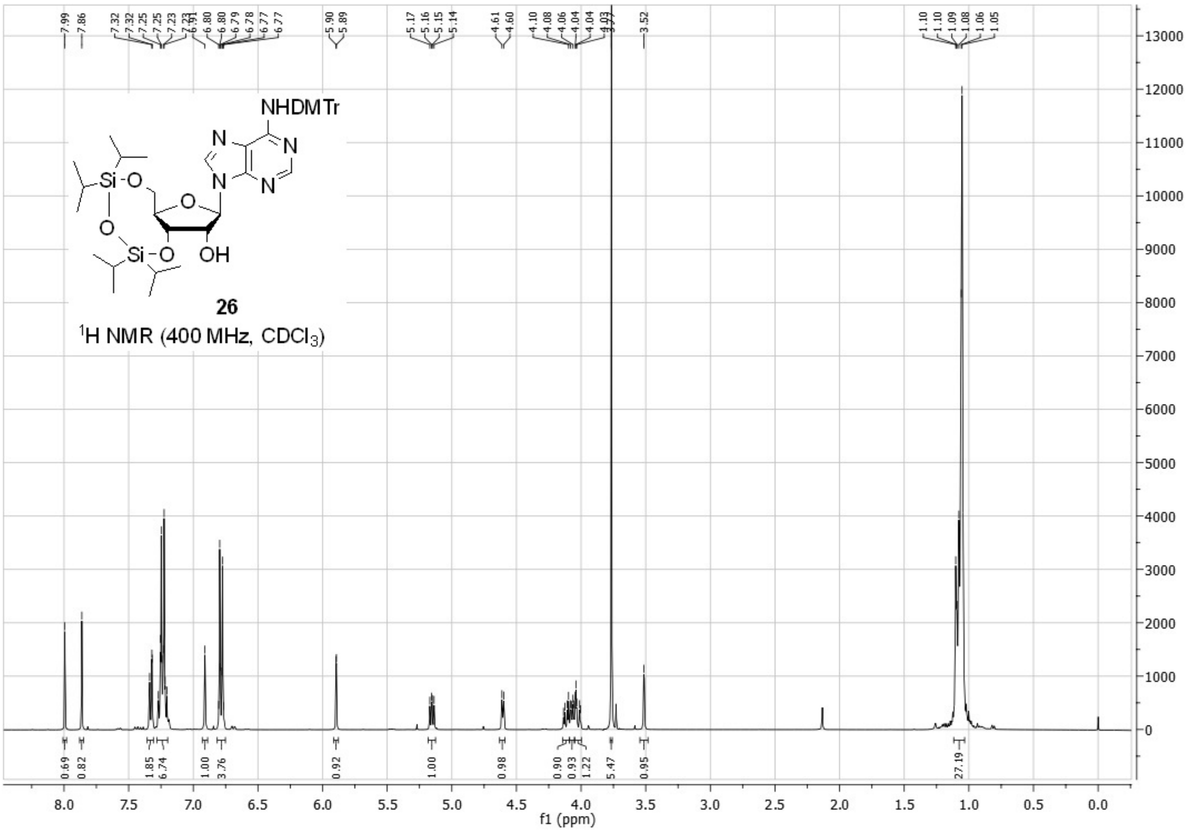
NHDMTr
26
^{1}H NMR (400 MHz, CDCl$_3$)
f1 (ppm)

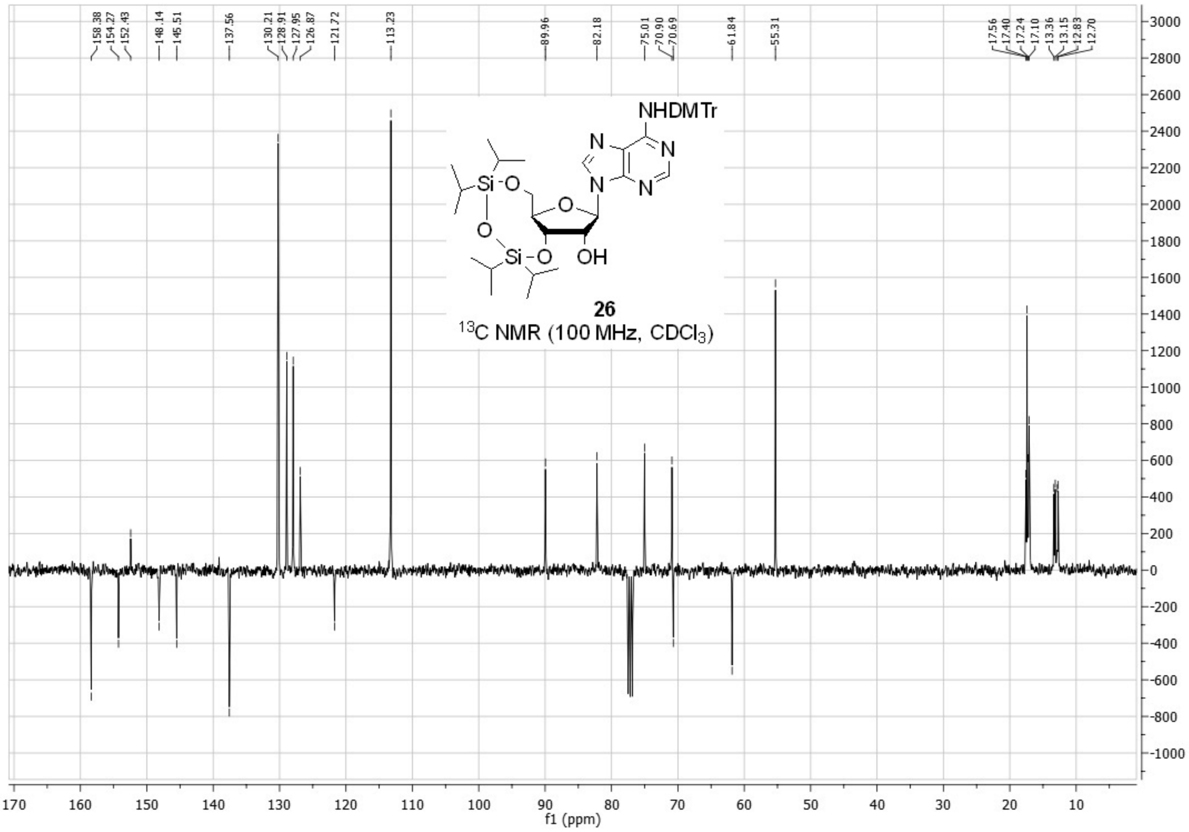
158.38
154.27
152.43
148.14
145.51
137.56
130.21
128.91
127.95
126.87
121.72
113.23
89.96
82.18
75.01
70.90
70.69
61.84
55.31
17.56
17.40
17.24
17.10
13.36
13.15
12.83
12.70
NHDMTr
OH
26
^{13}C NMR (100 MHz, CDCl$_3$)
f1 (ppm)

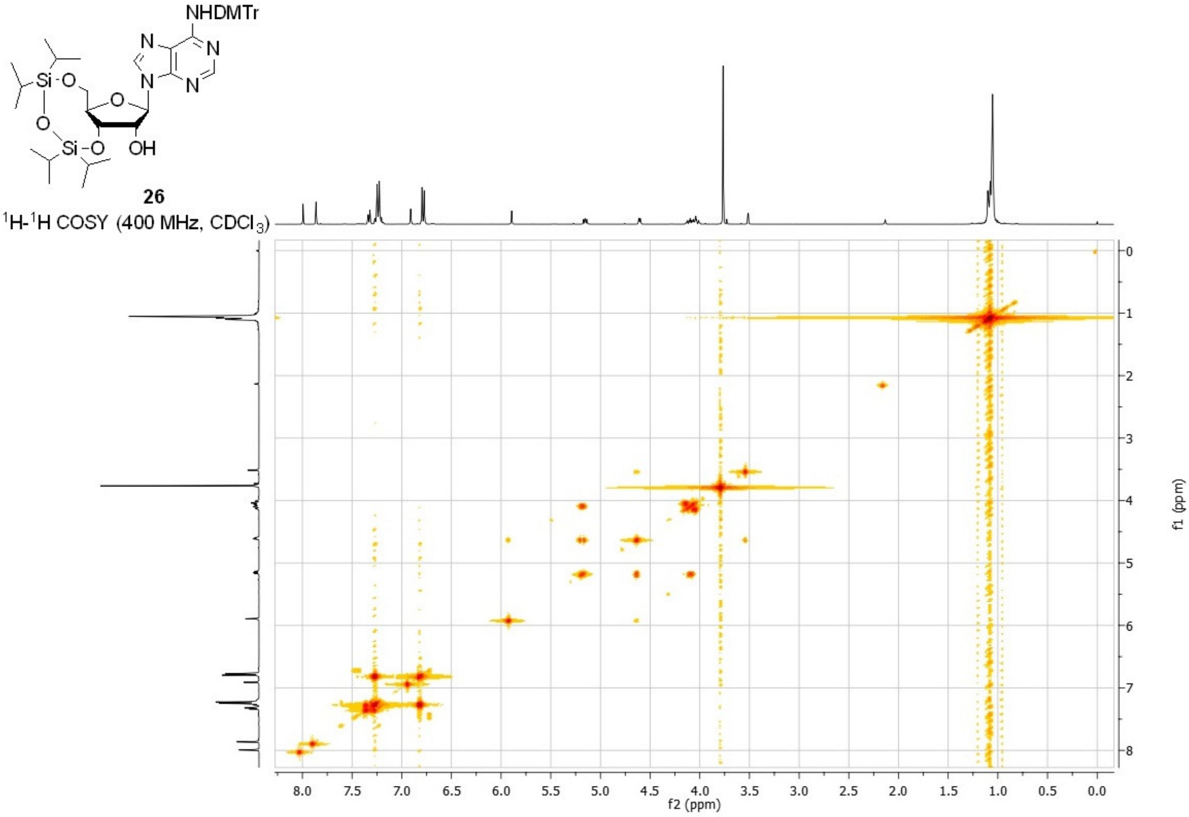
NHDMTr
OH
26
^{1}H-^{1}H COSY (400 MHz, CDCl$_3$)
f2 (ppm)
f1 (ppm)

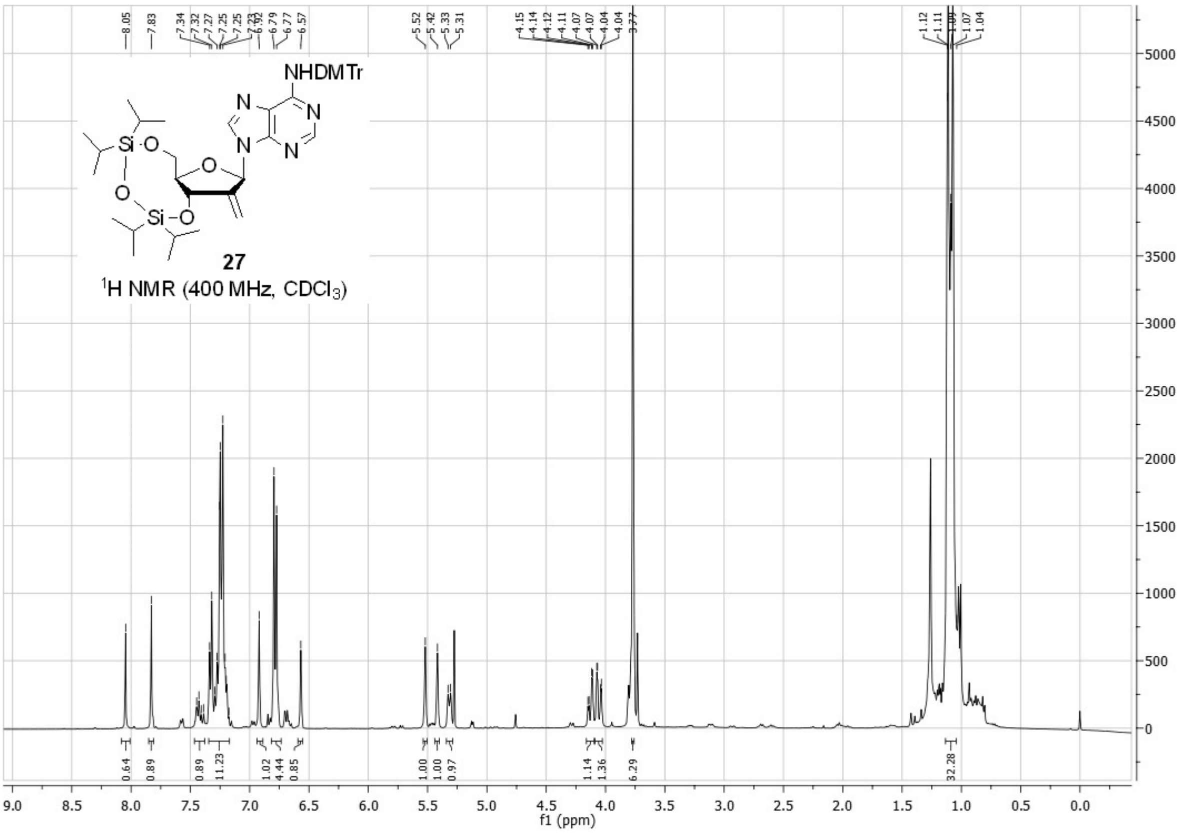

NHDMTr
27
1H NMR (400 MHz, CDCl3)
f1 (ppm)

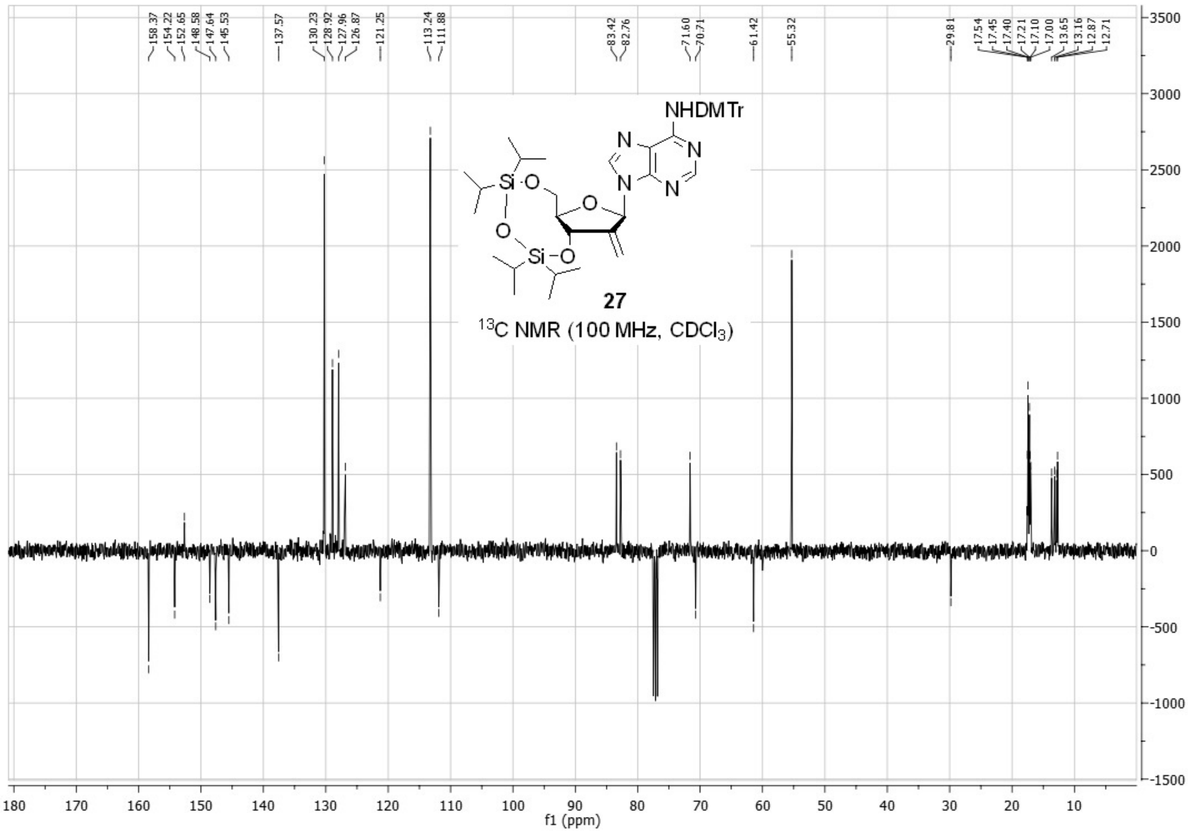

NHDMTr
27
13C NMR (100 MHz, CDCl3)
f1 (ppm)

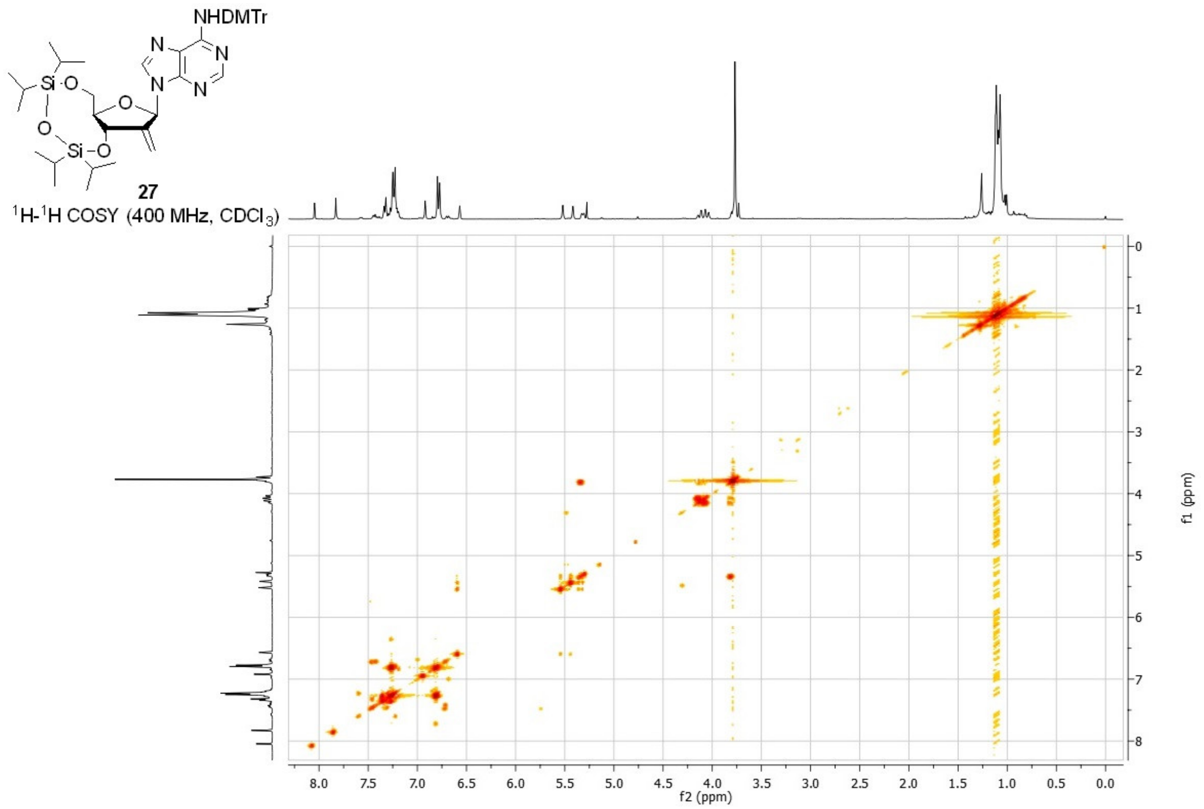
NHDMTr
27
^{1}H-^{1}H COSY (400 MHz, CDCl$_3$)
f2 (ppm)
f1 (ppm)

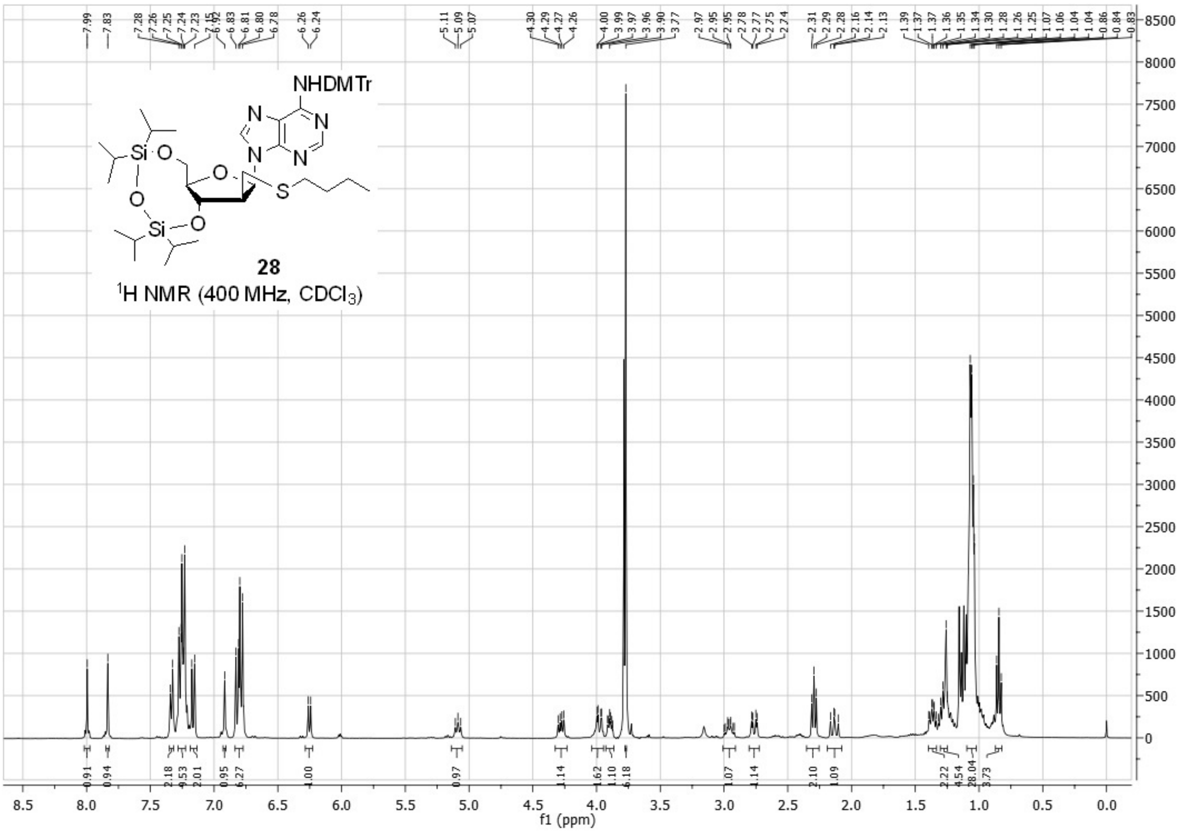
NHDMTr
28
^{1}H NMR (400 MHz, CDCl$_3$)
f1 (ppm)

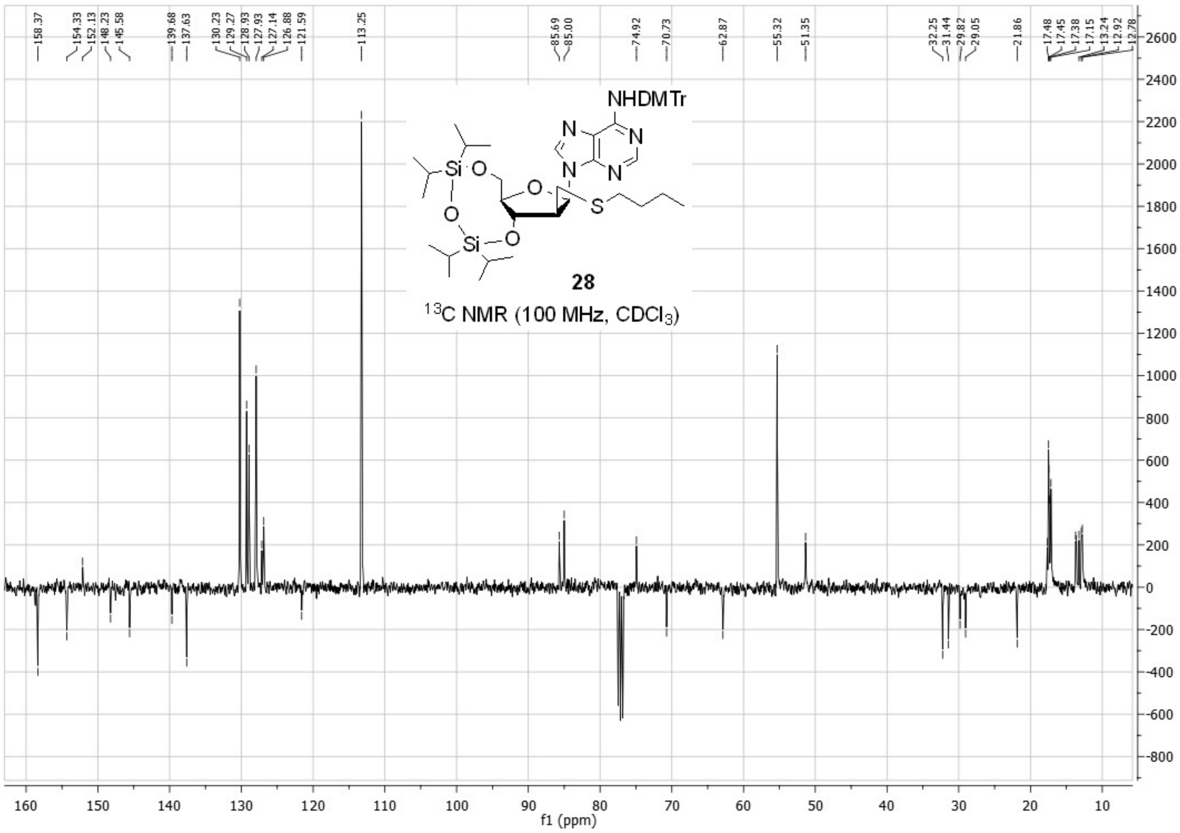
NHDMTr
28
^{13}C NMR (100 MHz, CDCl$_3$)
f1 (ppm)

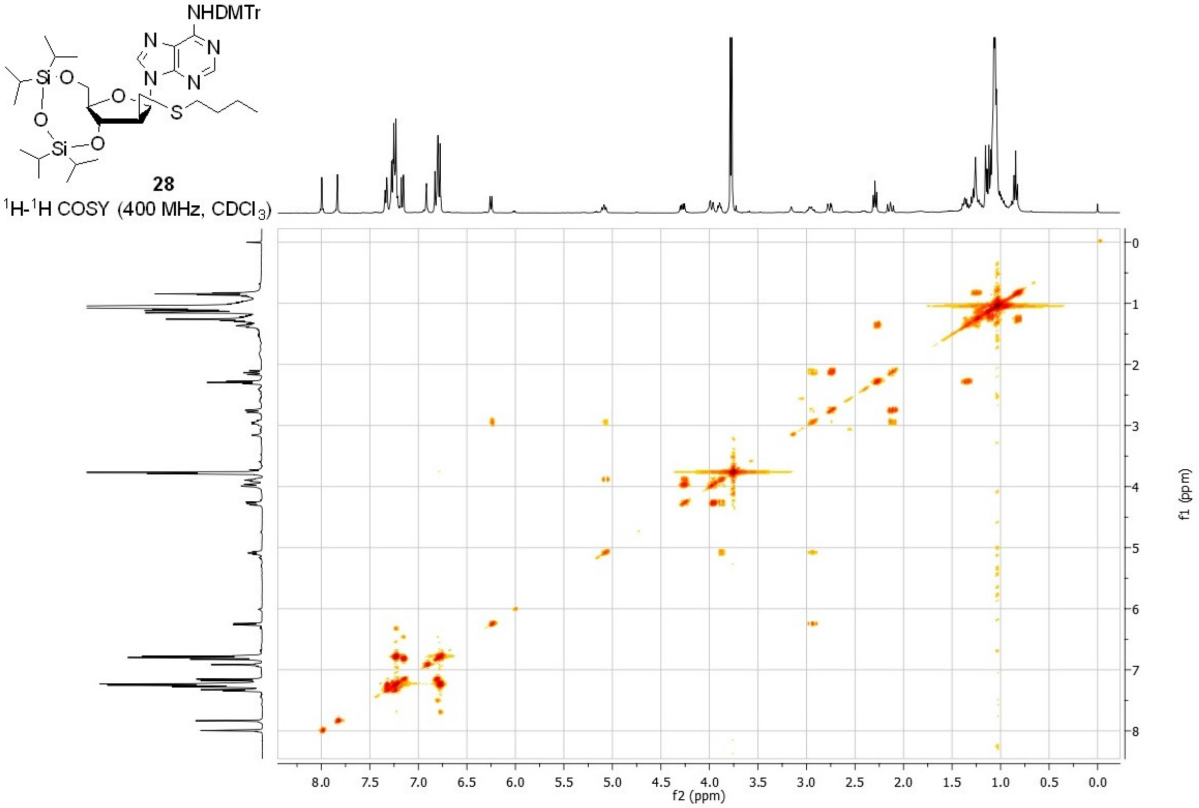
NHDMTr
28
^{1}H-^{1}H COSY (400 MHz, CDCl$_3$)
f2 (ppm)
f1 (ppm)

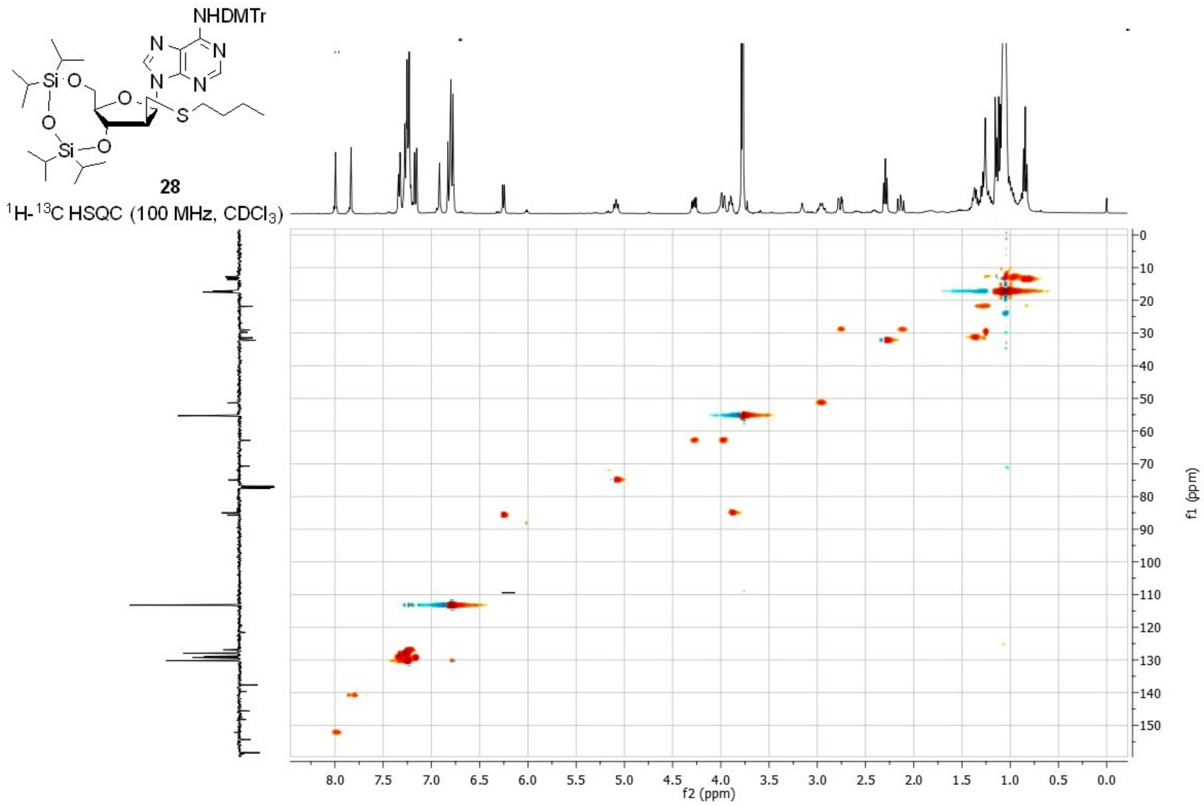

NHDMTr
28
^{1}H-^{13}C HSQC (100 MHz, CDCl$_3$)
f2 (ppm)
f1 (ppm)

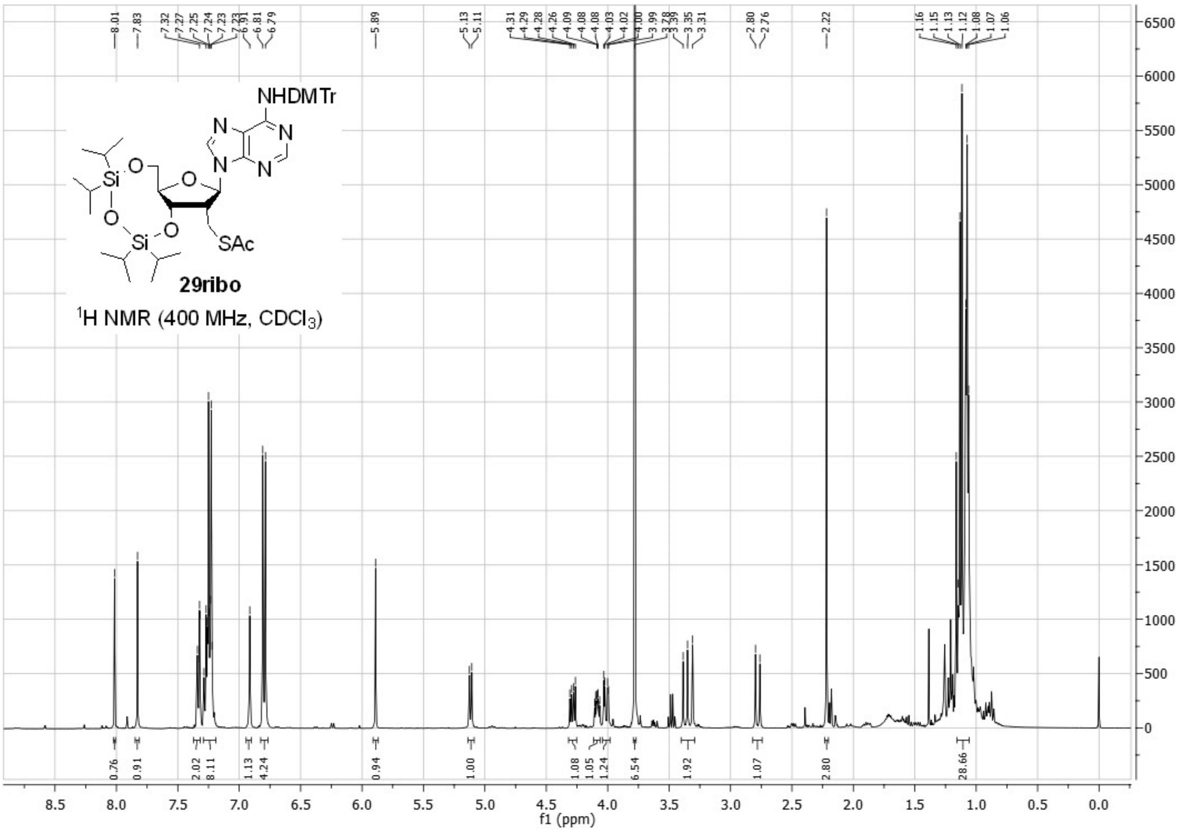

NHDMTr
SAc
29ribo
^{1}H NMR (400 MHz, CDCl$_3$)
f1 (ppm)

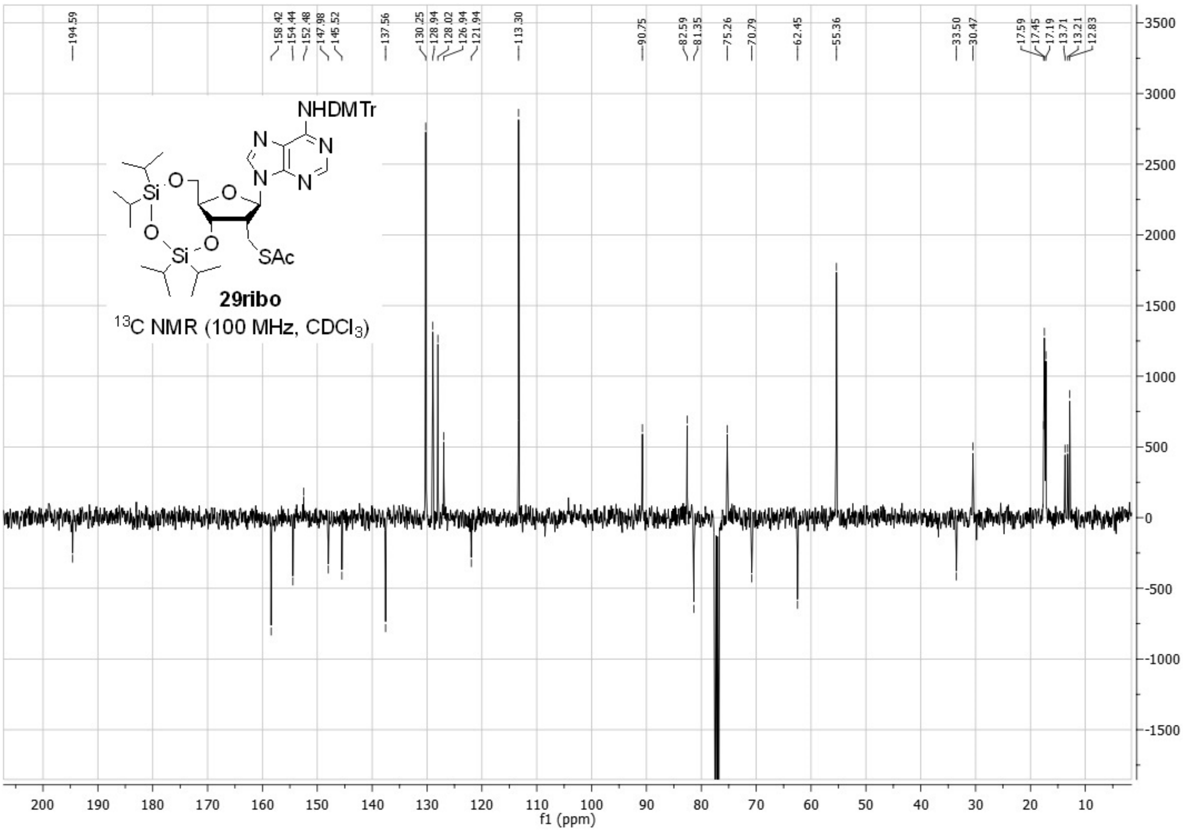
NHDMTr
SAc
29ribo
^{13}C NMR (100 MHz, CDCl$_3$)
f1 (ppm)

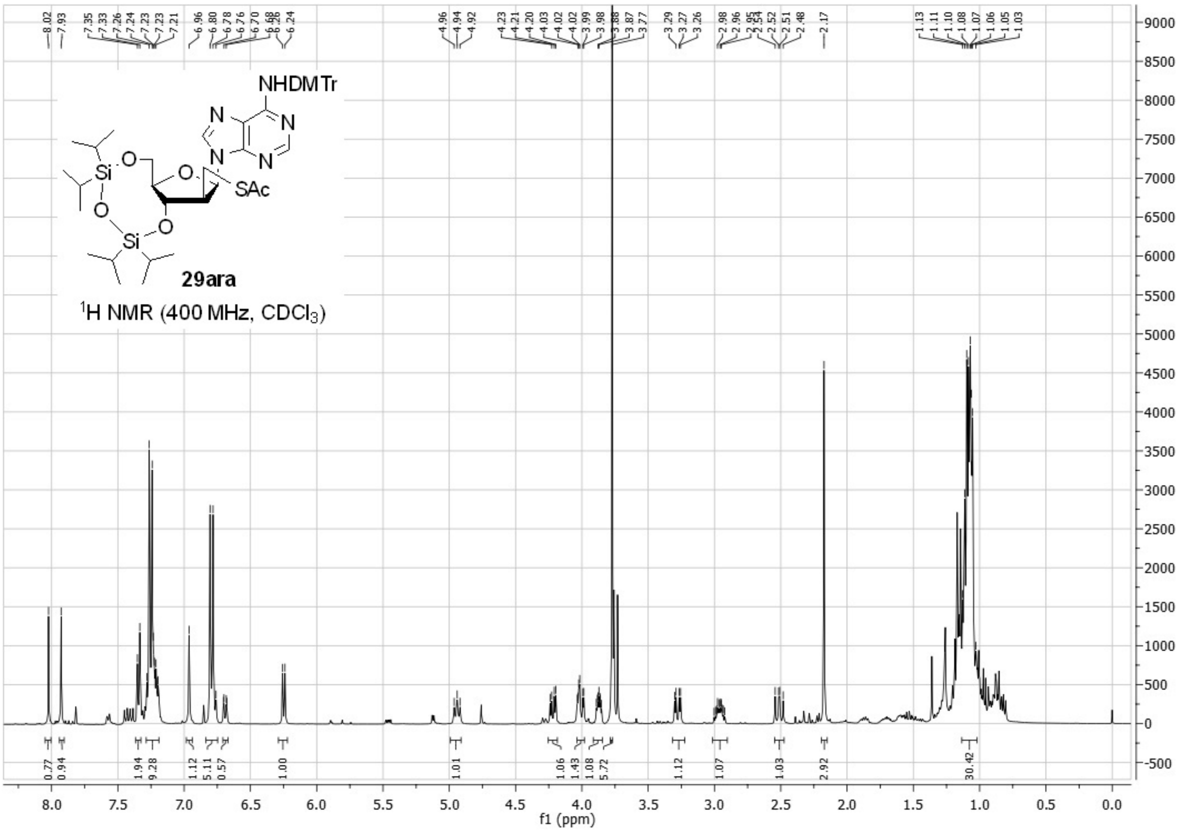
NHDMTr
SAc
29ara
^{1}H NMR (400 MHz, CDCl$_3$)
f1 (ppm)

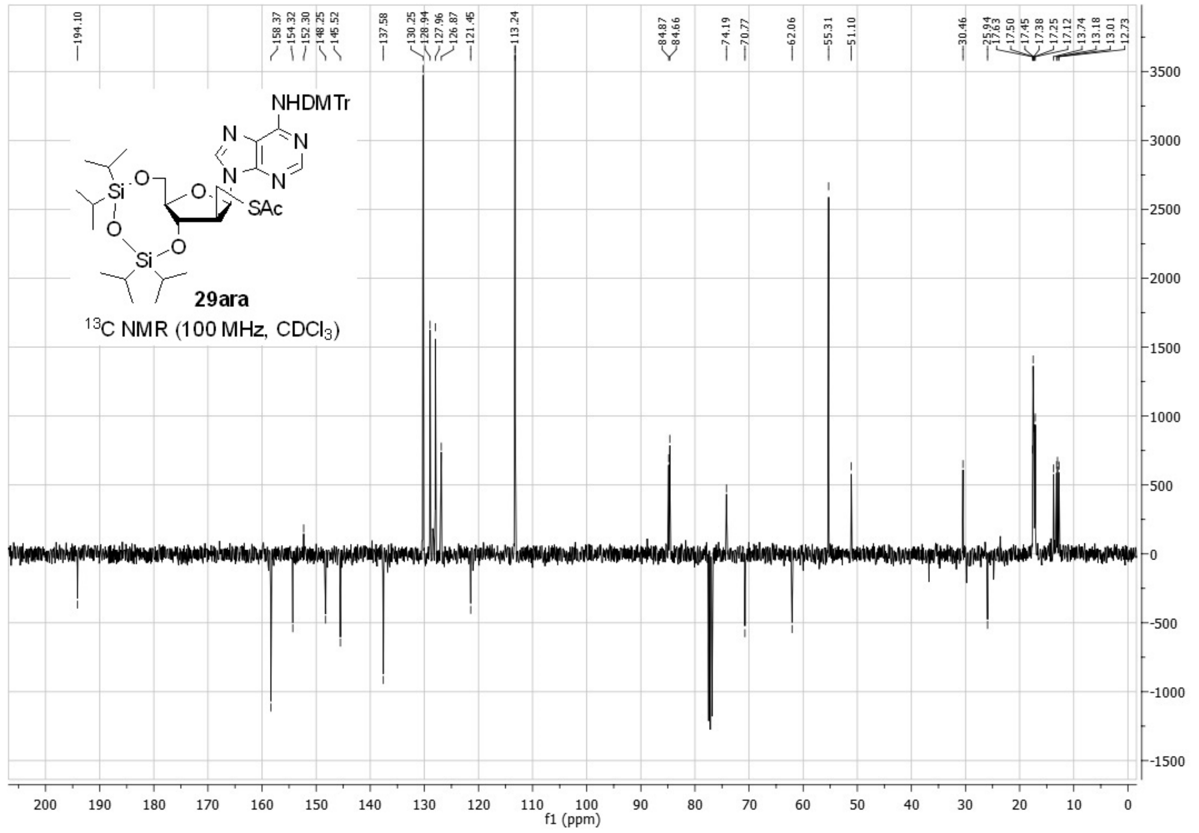
NHDMTr
Si
O
SAc
29ara
13C NMR (100 MHz, CDCl3)
f1 (ppm)

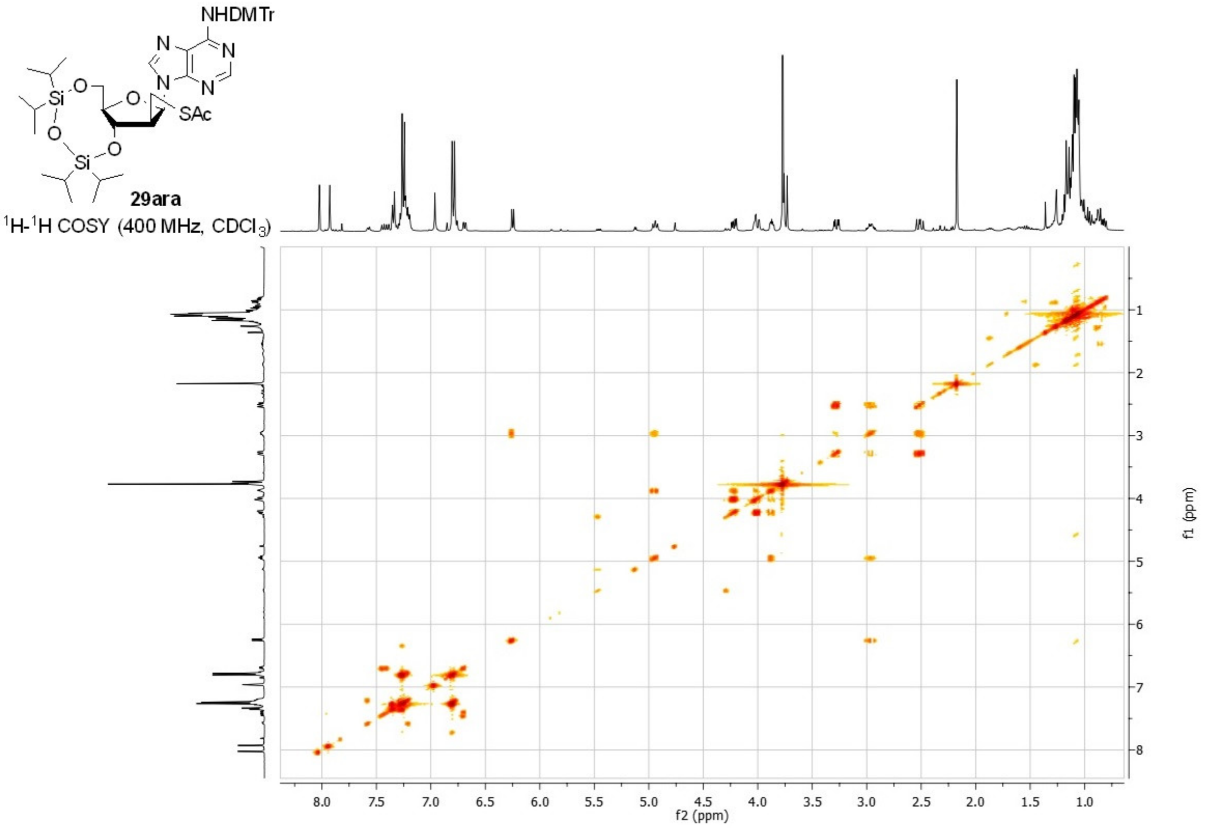
NHDMTr
29ara
1H-1H COSY (400 MHz, CDCl3)
f2 (ppm)
f1 (ppm)

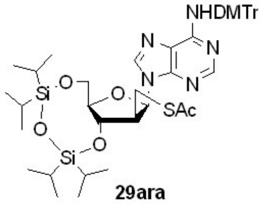

29ara

^{1}H-^{13}C HSQC (100 MHz, $CDCl_3$)

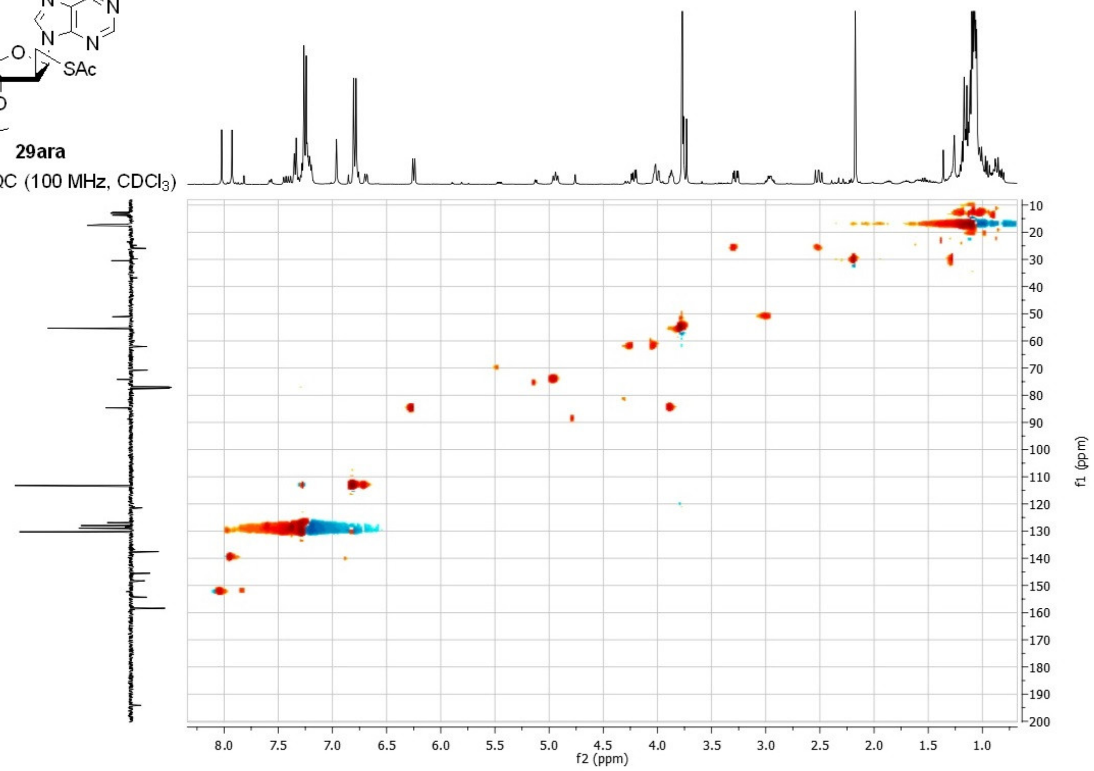

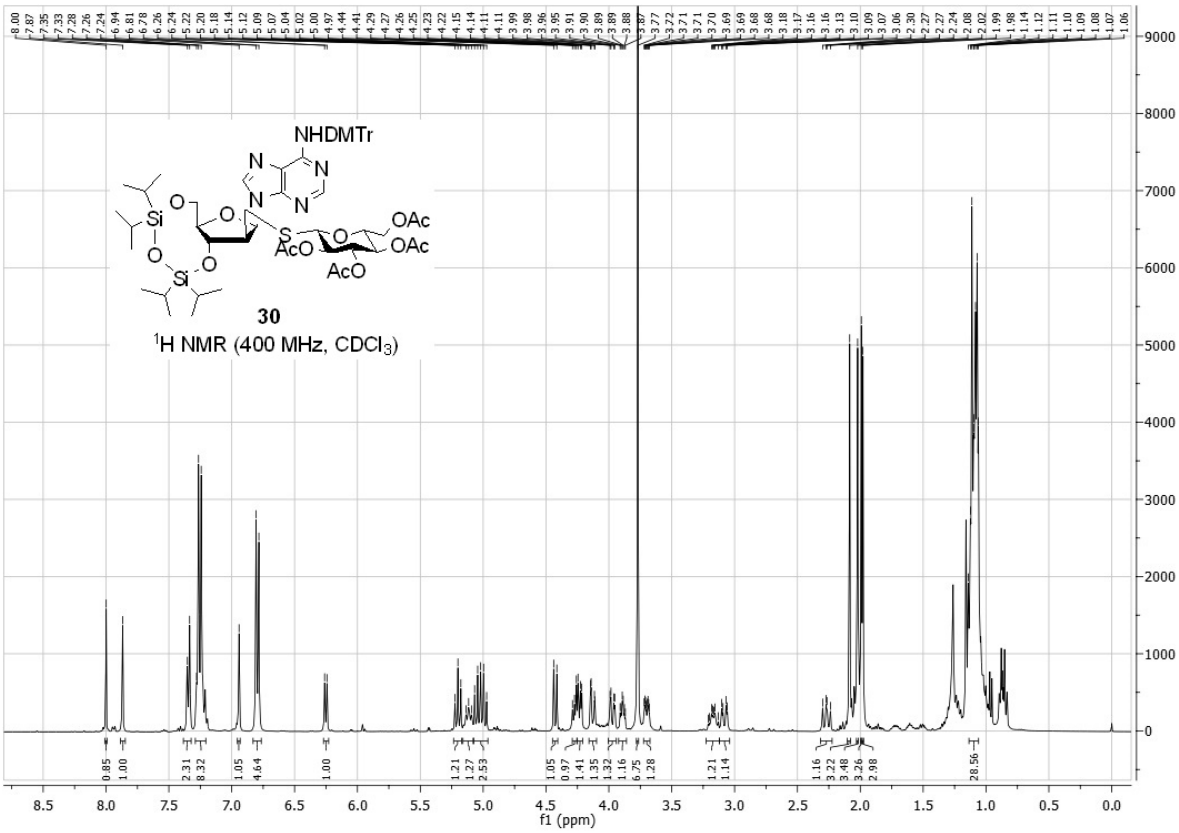

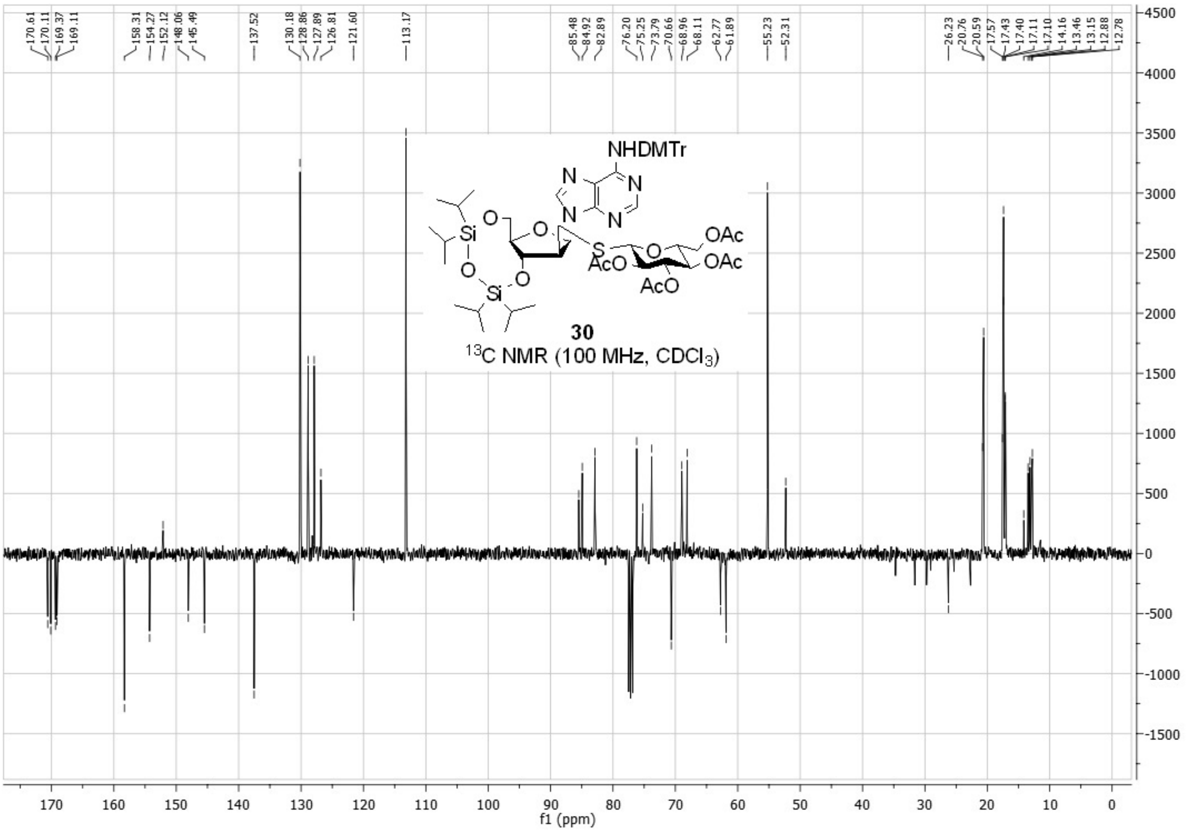

170.61 170.11 169.37 169.11 158.31 154.27 152.12 148.06 145.49 137.52 130.18 128.86 127.89 126.81 121.60 113.17 85.48 84.92 82.89 76.20 75.25 73.79 70.66 68.96 68.11 62.77 61.89 55.23 52.31 26.23 20.76 20.59 17.57 17.43 17.40 17.11 17.10 14.16 13.46 13.15 12.88 12.78
NHDMTr
OAc
OAc
AcO
AcO
30
^{13}C NMR (100 MHz, $CDCl_3$)
f1 (ppm)

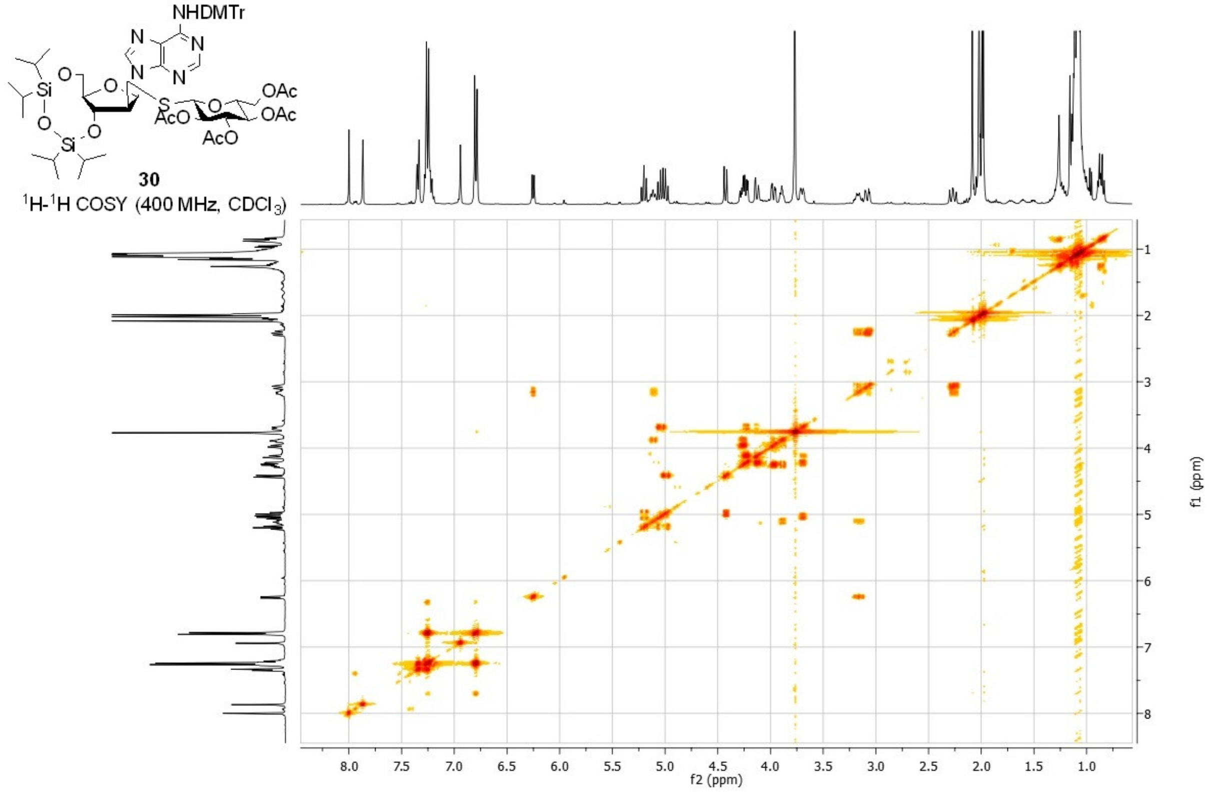

NHDMTr
OAc
OAc
AcO
AcO
30
^{1}H-^{1}H COSY (400 MHz, $CDCl_3$)
f2 (ppm)
f1 (ppm)

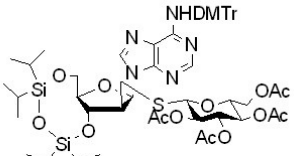

30

^{1}H-^{13}C HSQC (100 MHz, $CDCl_3$)

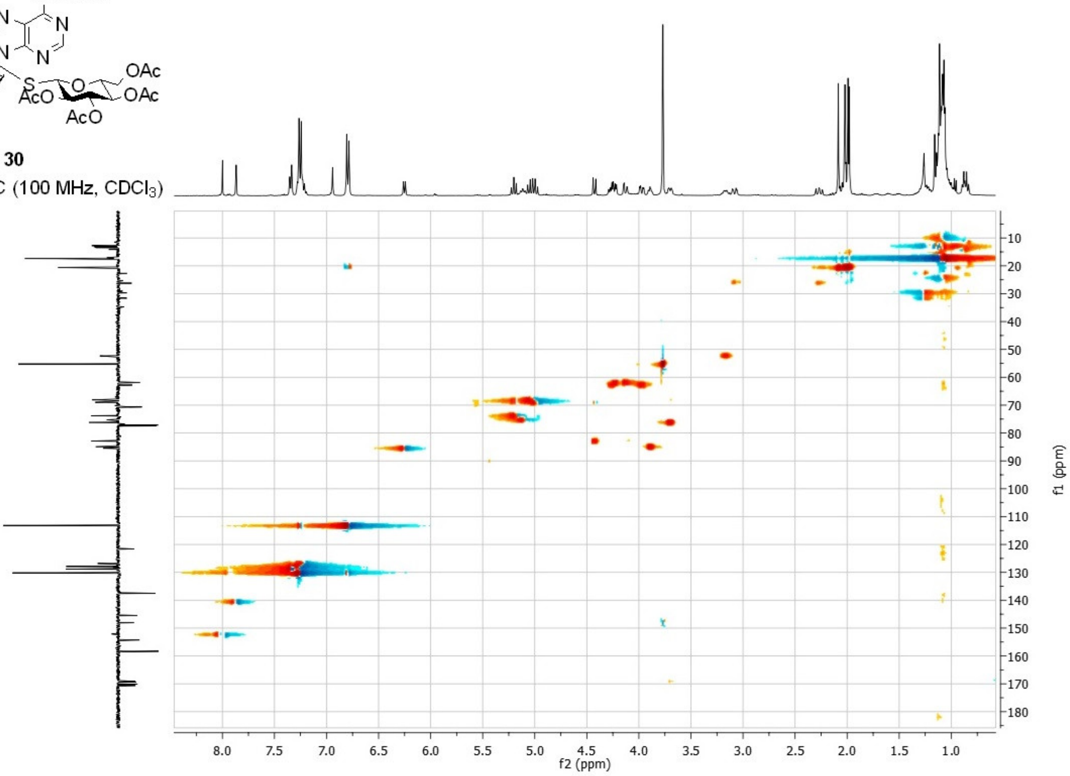

Supplement: Supplementary file 1 [file ijms-23-12566-s001.zip › ijms-1968286-supplementary.pdf]
